# An international validation study of the IL-2 Luc assay for evaluating the potential immunotoxic effects of chemicals on T cells and a proposal for reference data for immunotoxic chemicals

**DOI:** 10.1016/j.tiv.2020.104832

**Published:** 2020-03-18

**Authors:** Yutaka Kimura, Rie Yasuno, Mika Watanabe, Miwako Kobayashi, Tomoko Iwaki, Chizu Fujimura, Yoshihiro Ohmiya, Kohji Yamakage, Yoshihiro Nakajima, Mayumi Kobayashi, Nana Mashimo, Yumi Takagi, Takashi Omori, Emanuela Corsini, Dori Germolec, Tomoaki Inoue, Erwin L. Rogen, Hajime Kojima, Setsuya Aiba

**Affiliations:** a Department of Dermatology, Tohoku University Graduate School of Medicine, Sendai, Japan; b Biomedical Research Institute, National Institute of Advanced Industrial Science and Technology (AIST), Tsukuba, Japan; c Hatano Research Institute, Food and Drug Safety Center, Hadano, Japan; d Health Research Institute, National Institute of Advanced Industrial Science and Technology (AIST), Takamatsu, Japan; e Division of Biostatistics, Department of Social/Community Medicine and Health Science, Kobe University School of Medicine, Kobe, Japan; f Laboratory of Toxicology, Department of Pharmacological Sciences, Università degli Studi di Milano, Italy; g Division of the National Toxicology Program, National Institute of Environmental Health Sciences, USA; h Research Division, Chugai Pharmaceutical Co., Ltd., Japan; i 3RsMC Aps, Denmark; j Japanese Center for the Validation of Alternative Methods, National Institute of Health Sciences, Kawasaki, Japan

**Keywords:** Luciferase assay, IL-2, Validation study, Immunotoxic assay

## Abstract

To evaluate the immunotoxic effects of xenobiotics, we have established the Multi-ImmunoTox assay, in which three stable reporter cell lines are used to evaluate the effects of chemicals on the IL-2, IFN-γ, IL-1β and IL-8 promoters. Here, we report the official validation study of the IL-2 luciferase assay (IL-2 Luc assay). In the Phase I study that evaluated five coded chemicals in three sets of experiments, the average within-laboratory reproducibility was 86.7%. In the Phase II study, 20 coded chemicals were evaluated at multiple laboratories. In the combined results of the Phase I and II studies, the between-laboratory reproducibility was 80.0%. These results suggested that the IL-2 Luc assay was reproducible both between and within laboratories. To determine the predictivity, we collected immunotoxicological information and constructed the reference data by classifying the chemical into immunotoxic compounds targeting T cells or others according to previously reported criteria. When compared with the reference data, the average predictivity of the Phase I and II studies was 75.0%, while that of additional 60 chemicals examined by the lead laboratory was 82.5%. Although the IL-2 Luc assay alone is not sufficient to predict immunotoxicity, it will be a useful tool when combined with other immune tests.

## 1. Introduction

A well-functioning immune system is essential for maintaining the integrity of an organism. Immune dysregulation can have serious adverse health consequences, ranging from reduced resistance to infection and neoplasia to allergic and autoimmune conditions. Environmental contaminants, food additives, and drugs can target the immune system, resulting in immune dysregulation. Accordingly, the potential for immunotoxicity, which is defined as the toxicological effects of xenobiotics on the function of the immune system, has raised serious concerns from the public as well as from regulatory agencies. Currently, the assessment of chemical immunotoxicity relies mainly on animal models and *in vivo* assays, or *ex vivo* assays using cells from animals, to characterize immunosuppression and sensitization. However, animal studies have many drawbacks, such as high cost, ethical concerns, and questionable relevance to risk assessment for humans.

A worldwide vision is currently promoting alternative testing methods and assessment strategies to reduce the use of laboratory animals and, if possible, replace animals used in scientific studies (Adler et al., 2011). The workshop hosted by the European Centre for the Validation of Alternative Methods (ECVAM) in 2003 focused on stateof-the-art *in vitro* systems for evaluating immunotoxicity (Galbiati et al., 2010; Gennari et al., 2005; Lankveld et al., 2010). A tiered approach was proposed during the ECVAM workshop. Since useful information can be obtained from regular 28-day general toxicity tests, the proposed tiered approach would begin pre-screening for direct immunotoxicity by evaluating myelotoxicity, as compounds capable of damaging or destroying bone marrow will likely have immunotoxic effects (Lankveld et al., 2010). If compounds are not myelotoxic, they are tested for leukotoxicity. Compounds are then tested for immunotoxicity using various approaches, such as the T cell–dependent antibody response, lymphocyte proliferation assay, mixed lymphocyte reaction, NK cell assay, dendritic cell maturation assay, human whole-blood cytokine release assay (HWBCRA), and/or fluorescent cell chip (FCP) assay.

Several regulatory guidance or guidelines in immunotoxicology have been published for the pharmaceutical industry and chemical manufacturers. A workshop hosted by the International Life Sciences Institute-Health and Environmental Sciences Institute (ILSI-HESI) was held to share perspectives on immunotoxicity testing, developmental immunotoxicity and integrated and alternative approaches to immunotoxicity testing. The workshop summarized that standard toxicity studies, combined with triggered-based functional immune testing approaches, represent an effective approach to evaluate immunotoxic potential (Boverhof et al., 2014).

Our group established the Multi-ImmunoTox assay (MITA) to evaluate the effects of chemicals on the IL-2, IFN-γ, IL-1β, and IL-8 promoters using three stable reporter cell lines (Kimura et al., 2014, 2018). Of these cell lines, 2H4 derived from Jurkat cells contains stable luciferase green (SLG) regulated by the IL-2 promoter, stable luciferase orange (SLO) regulated by the IFN-γ promoter, and stable luciferase red (SLR) regulated by the G3PDH promoter (Saito et al., 2011). The IL-2 luciferase assay (IL-2 Luc assay) uses 2H4 cells to identify the effects of chemicals on the IL-2 and IFN-γ promoters in the presence of the stimulants phorbol 12-myristate 13-acetate (PMA) and ionomycin (Io).

IL-2 exerts pleiotropic actions on CD4+ T cell differentiation *via* its modulation of cytokine receptor expression. IL-2 promotes Th1 differentiation by inducing IL-12Rb2 (and IL-12Rb1), promotes Th2 differentiation by inducing IL-4Ra, inhibits Th17 differentiation by inhibiting gp130 (and IL-6Ra), and drives Treg differentiation by inducing IL-2Ra. IL-2 also potently represses IL-7Ra, which decreases survival signals that normally promote cell survival and memory cell development (reviewed by Liao et al. (2011)). It is therefore conceivable that chemicals that affect IL-2 release by T cells can significantly impact immune function.

Although our final goal is to officially validate the MITA as a defined approach for the *in vitro* assessment of immunotoxicity, in the current study we conducted a validation study for the IL-2 Luc assay. This validation study was conducted by a validation management team (VMT) composed of the lead laboratory, three independent laboratories, and four international expert members coordinated by the Japanese Center for the Validation of Alternative Methods (JaCVAM). This validation study examined the within- and between-laboratory reproducibility of this assay. In addition, it shed light on the difficulty in determining the predictivity of *in vitro* immunotoxicity tests because of the lack of reference data regarding the targeted effects of immunotoxic chemicals. In this study, we also proposed a procedure to create the reference data for assessing chemical immunotoxicity.

## 2. Materials and methods

### 2.1. The 2H4 IL-2 reporter cell line

We used the previously established 2H4 reporter cell line derived from a specific cell line of Jurkat cells with the ability to produce IL-2, kindly provided by Professor Kazuo Sugamura, Department of Microbiology, Tohoku University School of Medicine. 2H4 cells contain SLG regulated by the IL-2 promoter, SLO regulated by the IFN-γ promoter, and SLR regulated by the G3PDH promoter (Saito et al., 2011). This cell line was cultured in RPMI-1640 (Sigma-Aldrich, St. Louis, MO) containing Antibiotic-Antimycotic (Invitrogen) and 10% Hyclone™ fetal calf serum (Thermo Fisher Scientific, Waltham, MA) (Jurkat growth medium) at 37 °C with 5% CO_2_.

### 2.2. Chemical treatment of 2H4 cells and measurement of luciferase activity

Based on previous reports (Kimura et al., 2014; Saito et al., 2011), 2H4 cells (2 × 10^5^ cells/50 μl/well) in 96-well black plates (Greiner Bio-One GmbH, Frickenhausen, Germany) were pretreated with different concentrations of individual chemicals for 1 h. The 2H4 cells were then stimulated with 25 nM PMA and 1 μM ionomycin (PMA/Io) for 6 h. Three luciferase activities (SLG luciferase activity (SLG-LA), SLO luciferase activity (SLO-LA), and SLR luciferase activity (SLR-LA)) were simultaneously determined using a microplate-type luminometer with a multi-color detection system (Phelios; Atto Co., Tokyo, Japan) and Tripluc luciferase assay reagent (TOYOBO Co., Ltd., Osaka, Japan) according to the manufacturers’ instructions. Use of the 2H4 cell line enabled measurement of SLG-LA driven by the IL-2 promoter (IL2LA), SLO-LA driven by the INF-γ promoter (IFNLA), and SLR-LA driven by G3PDH (GAPLA) in 2H4 cells. In this study we used just the IL2LA and GAPLA. We accounted for variation in cell number and cell viability after chemical treatment by normalizing the data for IL2LA (nIL2LA) or IFNLA (nIFNLA) by dividing IL2LA or IFNLA, respectively, with GAPLA in the 2H4 cells. In addition, we calculated % suppression, % augmentation, and Inh-GAPLA as follows:

%suppression=(nIL2LAof2H4cellstreatedwithchemicals/nIL2LAofnon-treated2H4cells)×100;

%augmentation=(1−(nIL2LAof2H4cellstreatedwithchemicals/nIL2LAofnon-treated2H4cells))×100;

Inh-GAPLA=GAPLA of 2H4 cells treated with chemicals/GAPLA of untreated cells. Definitions of these terms are provided in Table 1.

### 2.3. Validation studies

In the pre-validation study, transferability of this assay was examined using five non-coded chemicals (2-aminoanthracene, citral, chloroquine diphosphate salt, dexamethasone and methylmercury(II) chloride) in four test facilities, including the lead laboratory. These chemicals were selected by the Chemical Selection Committee (CSC).

In the Phase I study, within- and between-laboratory reproducibility of this assay was examined using five coded chemicals in three test facilities. In the Phase II study, between-laboratory reproducibility was examined using 20 coded chemicals in three test facilities. These chemicals were selected by the CSC in the VMT based on the in-house dataset of the lead laboratory and published papers on *in vivo* or *in vitro* immunotoxicity tests. The chemicals were coded by JaCVAM as shown in Appendix Tables 1 and 2, and distributed to the test facilities. The study was conducted based on the Multi-ImmunoTox Assay protocol Ver. 011E (Appendix 1).

### 2.4. Criteria for judgment of chemicals

A positive control examining the induction of nIFNLA in 2H4 cells treated with PMA/Io and measurement of nIFNLA in non-treated 2H4 cells was required for each set of experiments. The fold induction of nIFNLA of PMA/Ionomycin wells without chemicals (= (nIFNLA of 2H4 cells treated with PMA/Ionomycin)/(nIFNLA of non-treated 2H4 cells)) was calculated. If the fold induction for the positive control was less than 3.0, the results obtained from the experiments were rejected.

Experiments for each chemical were repeated until two consistent suppressive (or stimulatory) results or two consistent “no effect results” were obtained. When two consistent results were obtained, the chemicals were classified as indicated by the consistent results according to three criteria. Chemicals which met the following three criteria were judged as positive:

1. The mean % suppression was ≥35 (suppressive) or ≤−35 (stimulatory) with statistical significance. The statistical significance was judged by its 95% confidence interval.
2. The result showed two or more consecutive suppressive (stimulatory) data points with statistical significance or one suppressive (stimulatory) data point with statistical significance and a trend in which at least three consecutive data points increase (or decrease) in a dose-dependent manner. In the latter case, the trend can cross 0, as long as only one data point shows the opposite effect without statistical significance.
3. Only data obtained at the concentration at which Inh-GAPLA is ≥0.05 contribute to the classification of the chemical.

If the three criteria are not met, the chemical would be classified as having ‘no effect’.

It is important to recognize that IL-2 exerts pleiotropic actions on CD4+ T cell differentiation *via* its modulation of cytokine receptor expression. Indeed, IL-2 promotes Th1 and Th2 differentiation, while it also drives Treg differentiation. These findings suggest that the augmentation of IL-2 transcription can lead to either immunostimulation or immunosuppression, depending on the surrounding tissue environment *in vivo.* Therefore, if the results in our assay indicated either augmentation or suppression, the chemical was considered as positive (P) and if not, was classified as having “no effect” (N).

### 2.5. The performance of the IL-2 Luc assay

To determine the performance of the IL-2 Luc assay, it is crucial to understand the immunotoxicological characteristics of the chemicals used in the validation. Since the IL-2 Luc assay evaluates IL-2 transcription by T cells, we attempted to classify the chemicals into two categories: (i) immunotoxic chemicals which target T cells (TTCs), including chemicals that directly affect T cell viability, T cell proliferation or T cell function and (ii) others (NTTCs), which included chemicals that were suggested to not directly affect T cell viability, T cell proliferation or T cell function. To define those chemicals that TTCs, we conducted a literature review focused on the available immunotoxicity data and the following endpoints:

1. Decreased thymus weight.
2. Increased or decreased IL-2, IFN-γ, or IL-4 mRNA expression or production by T cells *ex vivo*.
3. Increased or decreased IL-2, IFN-γ, or IL-4 mRNA expression or production by T cells *in vitro*.
4. Suppressed T cell proliferation.
5. Suppressed cytotoxic T cell response.
6. Other data that clearly indicated that one of the immunotoxic mechanisms of the chemical was attributed to an effect on T cells.

Then, according to the rationale for classifying immunotoxic chemicals reported by Luster et al. (1992b), we defined TTCs as chemicals that satisfied one of the following criteria and then constructed a reference database defining the immunotoxicities of the chemicals.

Criterion 1. Decreased thymus weight with additional one or more findings among endpoints 2 to 5.

Criterion 2. Increased or decreased mRNA expression or protein production in one or more cytokines in Endpoints 2 or 3 in multiple reports.

Criterion 3. Increased or decreased mRNA expression or protein production in two or more cytokines in Endpoints 2 or 3.

Criterion 4. The presence data suggesting that one of the immunotoxic mechanisms of the chemical was attributed to an effect on T cells in Endopoint 6.

Then, by comparing the results of the IL-2 Luc assay (positive or no effect) with the classification of the chemicals (TTC or NTTC), we calculated the accuracy, sensitivity and specificity of the IL-2 Luc assay in the validation study.

To classify the 25 chemicals used in the Phase I and II studies, we used the chemical information kindly provided by the National Toxicology Program (NTP) (Appendix 2). The reference database including the immunotoxicological characteristics of each chemical is shown in Appendix Table 3. The list of references is in Appendix 3.

### 2.6. Acceptance criteria

The within-laboratory reproducibility for all the test facilities was conducted by an independent biostatistical analysis using five coded chemicals and was overseen by the VMT. Based on the tentative acceptance criteria for the Phase I study, the concordance within laboratories was required to be greater than or equal to 80%. Twenty-five coded test items were selected to confirm between-laboratory reproducibility in the Phase I and II studies. At the end of testing, the test facilities submitted a QC-certified copy of the entire study dossier to the trial coordinator (study plan adhering to GLP principles, raw data, records, data analysis, and study report adhering to GLP principles). Based on the tentative acceptance criteria for the Phase I and II studies, the concordance for between-laboratory reproducibility was required to be greater than or equal to 80%.

### 2.7. IL-2 Luc assay dataset for 60 chemicals and for chemicals evaluated by the NTP

Based on the IL-2 Luc assay protocol (version 011E) and the criteria used in the validation study, the lead laboratory reevaluated the data for 60 chemicals reported previously (Kimura et al., 2018) and 31 chemicals of the 51 chemicals evaluated by the NTP (Luster et al., 1992b). Information regarding the immunotoxicity of these chemicals is summarized in Appendix Table 4. The list of references is in Appendix 4.

## 3. Results

### 3.1. Phase 0 study (technical transfer)

The preliminary test trial (Phase 0) was performed by the participating laboratories following the Multi-ImmunoTox Assay protocol Ver. 008.1E established by the lead laboratory, Tohoku University. Four laboratories participated in the Phase 0 study of the IL-2 Luc assay using the five open labeled chemicals 2-aminoantracene, citral, chloroquine diphosphate salt, dexamethasone and methylmercury(II) chloride, and conducted one analysis set (three experiments) for each chemical. The response patterns of the five chemicals were similar among the four laboratories. Based on the results, the VMT judged that technical and protocol transfer of the IL-2 Luc assay was acceptable. After the Phase 0 study, the protocol was modified to optimize assay performance, and refine the acceptance criteria and statistical analyses.

### 3.2. Phase I study (for within- and between-laboratory reproducibility and predictivity)

For the Phase I study, a total of five coded chemicals (four T cell targeting and one non-T cell targeting) were evaluated in three experimental sets consisting of three or more individual experiments for each chemicals using the Multi-ImmunoTox Assay protocol Ver. 011E established by the lead laboratory, Tohoku University.

The complete results of the Phase I study are shown in Table 2. The within-laboratory reproducibility was 80.0% (4/5), 100% (5/5), and 80.0% (4/5) in Lab. A, Lab. B, and Lab. C, respectively. The average was 86.7% (13/15). The between-laboratory reproducibility was 80.0% (4/5).

### 3.3. Phase II study (for between-laboratory reproducibility and predictivity)

The Phase II study for between-laboratory reproducibility and predictivity was conducted with a total of 20 coded chemicals (twelve T cell targeting, seven non-T cell targeting and one undetermined) evaluated in one experimental set using the Multi-ImmunoTox Assay protocol Ver. 011E. The complete results of the Phase II study are shown in Table 3. The between-laboratory reproducibility was 80% (16/20). To further evaluate the between laboratory reproducibility, all of the results from Phases I and II were combined. The reproducibility for the combined results was 80% (20/25), similar to that of the Phase II study alone (Table 4).

### 3.4. The predictivity of the IL-2 Luc assay in the validation studies, in the dataset composed of 60 chemicals, and in the evaluation of 31 chemicals from the NTP database

To examine the predictivity of the IL-2 Luc assay, we surveyed the literature for available *in vivo*, *ex vivo*, *in vitro* and mechanistic data on the immunotoxicity of the chemicals used in this study (Appendix 3 and 4). The *in vivo* data may include alterations in the weight of immune system organs such as spleen and thymus, delayed type hypersensitivity response (DTH), and the susceptibility to infection and resistance to transplanted tumors. The *ex vivo* data contain the effects of chemicals on cytokine production, T cell-dependent antibody response *in vitro*, as well as cytotoxic T cell response, mixed lymphocyte reaction, and T cell mitogen-induced proliferation using immune cells from animals treated with the chemicals *in vivo*. The *in vitro* data demonstrated the effects of the chemicals on cytokine production or on T cell proliferation after mitogen stimulation using cells or tissues from non-treated animals.

Using this information, we determined whether or not the chemicals TTCs by affecting T cell viability, proliferation or function, according to the rationale reported by Luster et al. (1992b) for classifying immunotoxic chemicals. Based on these criteria, the chemicals used in this validation study were classified as 16 TTCs, 8 NTTCs, and 1 chemical could not be classified (Appendix Table 3). Then, by comparing the results of the IL-2 Luc assay (positive or no effect) with the classification of the chemicals (TTC or NTTC), we calculated the accuracy, sensitivity and specificity of the IL-2 Luc assay in the validation study.

In the Phase I study (Table 2), the accuracy was 80.0% (4/5), 100% (5/5), and 100% (5/5) in Lab. A, Lab. B, and Lab. C, respectively. The average was 93.3% (14/15). The sensitivity and specificity were 91.7% (11/12) and 100% (3/3), respectively.

In the Phase II study (Table 3), the accuracy was 73.7 (14/19), 68.4% (13/19), and 68.4% (13/19) in Lab. A, Lab. B, and Lab. C, respectively. The average was 70.2% (40/57). The sensitivity was 75.0% (9/12), 66.7% (8/12), and 66.7% (8/12) in Lab. A, Lab. B, and Lab. C, respectively. The average was 69.4% (25/36). The specificity was 71.4% (5/7), 71.4% (5/7), and 71.4% (5/7) in Lab. A, Lab. B, and Lab. C, respectively. The average was 71.4% (15/21).

In the combined results of the Phase I and Phase II studies (Table 4), the accuracy was 75.0% (18/24), 75.0% (18/24), and 75.0% (18/24) in Lab. A, Lab. B, and Lab. C, respectively. The average was 75.0% (54/ 72). The sensitivity was 75.0% (12/16), 75.0% (12/16), and 75.0% (12/16) in Lab. A, Lab. B, and Lab. C, respectively. The average 75.0% (36/48). The specificity was 75.0% (6/8), 75.0% (6/8), and 75.0% (6/ 8) in Lab. A, Lab. B, and Lab. C, respectively. The average was 75.0% (18/24).

In addition, the lead laboratory reevaluated the data for the 60 chemicals reported previously (Kimura et al., 2018) by the same criteria used in the validation study. The classification of the 60 chemicals and their immunotoxicological information are summarized in Appendix Table 4. The chemicals were classified as 34 TTCs, 6 NTTCs, and 20 chemicals that lacked sufficient information to classify their immunotoxic activity. The performance of the IL-2 Luc assay examining these 60 chemicals for sensitivity, specificity and accuracy was 82.4% (28/34), 83.3% (5/6), and 82.5% (33/40), respectively.

The lead laboratory also examined 31 of the 51 chemicals evaluated by Luster et al. (1992b) and thus we compared the results of the IL-2 Luc assay with their classification of immunotoxic chemicals in Appendix Table 5. Although the results were preliminary because of the limited number of chemicals used, the sensitivity, specificity and accuracy of the IL-2 Luc assay for these chemicals was 59.1% (13/22), 44.4% (4/9), and 54.8% (17/31) (Table 5).

## 4. Discussion

We examined within-laboratory reproducibility in the Phase I study. Lab. A, Lab. B, and Lab. C demonstrated 80%, 100%, and 80% reproducibility, respectively. On the other hand, Lab. A, Lab. B, and Lab. C demonstrated 80% between-laboratory reproducibility in the combined data of the Phase I and Phase II studies. These results satisfied the acceptance criteria for the validation study with a within-laboratory reproducibility of at least 80% and a between-laboratory reproducibility of at least 80%.

Reference data that indicate which chemicals are immunotoxic are indispensable for determining the performance of the IL-2 Luc assay. However, such reference data are lacking for most chemicals and thus we attempted to create reference data for the chemicals used in this study. Although there is no gold standard to date for identifying immunotoxic chemicals, Luster et al. (1992b) proposed a rationale for classifying immunotoxicants based on their ability to produce a significant dose-response effect in a single immune test or significantly alter two or more test results at the highest dose of the chemical tested. They classified chemicals based on the results obtained in immune tests according to this rationale and found a significant correlation between the judgment of immunotoxic chemicals and host resistance (Luster et al., 1993). Therefore, we used this rationale and classified chemicals based on the immunotoxicological information for each chemical published previously

Then, by comparing the results of the IL-2 Luc assay (positive or no effect) with the reference database we created, we calculated the performance of the IL-2 Luc assay. The average of the accuracy of the combined results of the Phase I and Phase II studies was 75.0% (54/72), while the accuracy obtained using the 60 chemicals was 82.5% (33/40).

In our previous study in which only immunosuppressive drugs whose effects on human have been well established were examined by the IL-2 Luc assay (Kimura et al., 2014), we demonstrated that tacrolimus (TAC), cyclosporine A (CyA) and dexamethasone (Dex) significantly suppressed IL-2 luciferase activity (IL-2 LA), although the average Lowest Observed Effect Levels (LOELs) of TAC and CyA were significantly lower that of DEX. The off-label immunosuppressive drugs chloroquine, minocycline, dapsone, and colchicine significantly suppressed IL-2 LA. The anti-cancer drugs actinomycin D and cisplatin and a representative immunosuppressive drug, azathioprine, also significantly suppressed IL-2 LA. No suppressive effects on IL-2 LA were demonstrated by several immunosuppressants whose mechanism of action is dependent on the inhibition of DNA synthesis or anti-proliferative effects on T cells, such as rapamycin, mizoribine, cyclophosphamide, methotrexate and mycophenolic acid. These data suggest that IL-2 LA is an assay most suitable to detect chemicals that affect cytokine production.

The HWBCRA, previously examined in a rigorous prevalidation effort by ECVAM and other groups, is an immune test to examine the effects of chemicals on IL-4 or IL-1β production stimulated by staphylococcal enterotoxin B (SEB) or LPS, respectively (Langezaal et al., 2001, 2002). This assay uses human whole blood cells and examines the production of IL-4 by T cells and of IL-1β by monocytes. This concept is similar to that of the MITA, in which the effects of chemicals on T cells and monocytes are examined using Jurkat cell-derived 2H4 and THP-1derived THP-G1b cells. Interestingly, the evaluation of chemicals by IL-4 production in the HWBCRA was almost identical to the results of the IL-2 Luc assay: both detected strong immunosuppression by TAC, CyA, DEX and actinomycin D, which are more potent than chloroquine and azathioprine. The cardiac glycoside digoxin is classified as an immunotoxic chemical by both assays. Cyclophosphamide and mizoribine require metabolic activation and thus are not judged as immunosuppressive in either assay. In addition, the HWBCRA is also considered to be unsuitable for detecting immunotoxic chemicals whose effects are dependent on suppressing cell proliferation.

The ability to similarly detect known immunosuppressive chemicals suggests that the IL-2 Luc assay may be a useful alternative to the HWBCRA for examining the effects of chemicals on T cells. In addition, the IL-2 Luc assay has a number of advantages over the HWBCRA, including: 1) The IL-2 Luc assay does not require primary cells, 2) it does not require cytokine quantification using ELISA, and 3) the time required for the IL-2 Luc assay is less than 8 h. However, similar to the HWBCRA, the IL-2 Luc assay cannot detect immunosuppression in chemicals whose effects depend on the suppression of cell proliferation or require metabolic activation. Therefore, these chemicals are considered as those out of applicability domain.

Luster et al. (1988) proposed a screening battery using a ‘tier’ approach for detecting potential immunotoxic compounds in mice. Then, they defined criteria to classify immunotoxic chemicals using several parameters comprising the ‘tier approach’ and classified 51 chemicals into immunotoxic and non-immunotoxic compounds (Luster et al., 1992b). Furthermore, they examined the ability of various immune tests to predict increased susceptibility for a number of models of disease resistance (Luster et al., 1992a). Their final results demonstrated that: 1) A number of the immune tests provided a relatively high association with changes in host resistance (*i.e.*, > 70%) such as IgM plaque forming cell (PFC) response to sheep red blood cells, T cell mitogen response, DTH, surface markers, and spleen cellularity. In contrast, several of the tests, such as leukocyte counts and lymphoproliferative response to lipopolysaccharide (LPS) were poor predictors, with concordance values of approximately 50%. 2) The combination of two immune tests compared with the host resistance classification increased the concordance from that obtained using individual tests. Pairwise combinations which included either the PFC response, surface markers, or DHRs gave consistently higher concordances.

When the IL-2 Luc assay examined 31 of the 51 chemicals evaluated by Luster et al. (1992b), its performance was similar to that of mixed lymphocyte reaction (MLR), DHR, and spleen cellularity and better than leukocyte counts or LPS response. Moreover, among 7 chemicals judged as false negative by the IL-2 Luc assay, 5 chemicals was judged as positive by Luster et al. (1992b) based on their suppressive effects on T cell mitogen response. Since our previous study demonstrated the inability of the IL-2 Luc assay to detect immunosuppressive effects of chemicals which are dependent on their suppressive effects on T cell proliferation, these 5 chemicals are out of applicability domain. Taking this into account, the sensitivity, specificity and accuracy of the IL-2 Luc assay was 76.5% (13/17), 44.4% (4/9), and 65.4% (17/26).

Thus, we would like to propose the MITA for *in vitro* testing to detect immunotoxic chemicals in future. The MITA can evaluate the effects of chemicals on IL-2, IFN-γ, IL-1β and IL-8 promoter activities. The induction of these cytokines is mediated by a wide range of signaling pathways, including as a minimum the MAP kinase, NF-kB, and calcium/calmodulin pathways. It is also well known that the induction of different immune-related molecules such as cytokines or chemokines commonly uses at least one of these signaling pathways. Therefore, although the MITA evaluates only the effects of chemicals on the transcription of four cytokines, it may be able to assess the effects of chemicals on a much wider range of immune responses. When combined with other *in vitro* assays, such as assessment of myelotoxicity or T cell mitogen responses, the predictivity of the MITA would be increased. Furthermore, the combination of the MITA with the IL-8 Luc assay (OECD442E) can evaluate the effects of chemicals on T cells and macrophages, and the sensitizing potentials of chemicals. The data obtained from these assays can be used by both industry and regulatory agencies to assess the immunotoxicity risks of chemicals.

## Supplementary Material

1

2

3

4

5

6

7

8

9

## Figures and Tables

**Table 1:** The definition of the parameters in the IL-2 Luc assay.

Abbreviations	Definition

GAPLA	SLR luciferase activity reflecting GAPDH promoter activity
IL2LA	SLO luciferase activity reflecting IL-2 promoter activity of 2H4 cells
nIL2LA	IL2LA/GAPLA of 2H4 cells
% suppression	(nIL2LA of 2H4 cells treated with chemicals/ nIL2LA of non-treated 2H4 cells) × 100
% augmentation	(1-(nIL2LA of 2H4 cells treated with chemicals/ nIL2LA of non-treated 2H4 cells)) × 100
CV05	The lowest concentration of the chemical at which Inh-GAPLA becomes < 0.05.
Inh-GAPLA	GAPLA of 2H4 cells treated with chemicals /GAPLA of untreated cells.

**Table 2:** Results of the Phase I study.

Chemical	CAS	Set	Lab. A	Lab. B	Lab. C	Concordance	T cell targeting	Rationale

Dibutyl phthalate	84-74-2	1st	P	P	P	1	Yes	3, 4
		2nd	P	P	P			
		3rd	P	P	P			
Hydrocortisone	50-23-7	1st	P	P	P	0	Yes	1
		2nd	N	P	P			
		3rd	N	P	N			
Lead(II) acetate	6080-56-4	1st	P	P	P	1	Yes	1
		2nd	P	P	P			
		3rd	P	P	P			
Nickel(II) sulfate	10101-97-0	1st	P	P	P	1	Yes	1
		2nd	P	P	P			
		3rd	P	P	P			
Zinc dimethyldithiocarbamate (DMDTC)	137-30-4	1st	N	N	N	1	No	
		2nd	N	N	N			
		3rd	N	N	N			
Within-laboratory reproducibility (%)			80.0 (4/5) Average 86.7 (13/15)	100 (5/5)	80.0 (4/5)			
Between-laboratory reproducibility (%) (Based on Majority)						80 (4/5)		
Sensitivity (%) (Based on Majority)			75.0 (3/4) Average 91.7 (11/12)	100 (4/4)	100 (4/4)			
Specificity (%) (Based on Majority)			100 (1/1) 100 (3/3)	100 (1/1)	100 (1/1)			
Accuracy (%) (Based on Majority)			80.0 (4/5) Average 93.3 (14/15)	100 (5/5)	100 (5/5)			

P: Positive, N: No effect.

**Table 3:** Results of the Phase II study.

Chemical	CAS	Lab.A	Lab.B	Lab.C	Concordance	T cell targeting	Rationale

2,4-Diaminotoluene	95-80-7	N	N	N	1	No	
Benzo(a)pyrene	50-32-8	P	P	P	1	Yes	2), 3)
Cadmium chloride	10108-64-2	N	N	N	1	Yes	2), 3)
Dibromoacetic acid	631-64-1	P	P	N	0	Yes	1), 4)
Diethylstilbestol	56-53-1	P	P	P	1	Yes	1), 2), 4)
Diphenylhydantoin	630-93-3	N	N	N	1	Yes	2), 3), 4)
Ethylene dibromide	106-93-4	N	N	N	1	Yes	1)
Glycidol	556-52-5	P	P	P	1	No	
Indomethacin	53-86-1	P	P	P	1	Yes	3), 4)
Isonicotinic acid Hydrazide	54-85-3	P	N	P	0	Yes	2)
Nitrobenzene	98-95-3	N	P	N	0	Undetermined	
Urethane, Ethyl carbamate	51-79-6	P	P	P	1	Yes	1)
Tributyltin chloride	1461-22-9	P	P	P	1	Yes	1)
Perfluorooctanoic acid	335-67-1	P	P	P	1	Yes	1)
Dichloracetic acid	79-43-6	P	P	P	1	Yes	2), 3)
Toluene	108-88-3	N	N	N	1	No	
Acetonitril	75-05-8	N	N	N	1	No	
Mannitol	69-65-8	N	N	N	1	No	
Vanadium pentoxide	1314-62-1	N	N	N	1	No	
o-Benzyl-p-chorolophenol	120-32-1	P	P	P	1	No	
Between-laboratory reproducibility (%)					80 (16/20)		
Sensitivity (%)		75.0 (9/12)	66.7 (8/12)	66.7 (8/12)			
Specificity (%)		71.4 (5/7)	71.4 (5/7)	71.4 (5/7)			
Accuracy (%)		73.7 (14/19)	68.4 (13/19)	68.4 (13/19)			

P: Positive, N: No effect.

**Table 4:** The combined results of the Phase I and Phase II studies.

Within-laboratory reproducibilities (%)	80 (4/5) Average 86.7 (13/15)	100 (5/5)	80 (4/5)	
Between-laboratory reproducibilities (%) (Based on majority for Phase I)				80 (20/25)
Sensitivity (%)	75.0 (12/16) Average 75.0 (36/48)	75.0 (12/16)	75.0 (12/16)	
Specificity (%)	75.0 (6/8) Average 75.0 (18/24)	75.0 (6/8)	75.0 (6/8)	
Accuracy (%)	75.0 (18/24) Average 75.0 (54/72)	75.0 (18/24)	75.0 (18/24)	

**Table 5:** Data set for the IL-2 Luc assay.

Chemical name	Immunatoxicity classification		IL-2 Luc assay	Ave.LOEL(35%)	Ave.LOEL(−35%)
	Classification	Rationale^#^			
FK506	TTC	1,3	P	0.0002	
Cyclosporine A	TTC	1,3	P	0.0041	
Actinomycin D	TTC	3	P	0.0156	
Digoxin	TTC	2,3	P	0.0686	
Colchicine	TTC	2,3	P	0.2743	
FR167653	Undetermined	2,3	P	1.3021	
Benzethonium chloride	Undetermined	1	P	1.6276	
Mercuric chloride	TTC	1,3	P	1.9531	
Chlorpromazine	TTC	1,3	P	1.9531	
Amphotericin B	Undetermined	1	P	2.6042	
Dibutyl phthalate	TTC	3	P	2.6042	
2-Aminoanthracene	Undetermined		P	5.8594	
Formaldehyde	TTC	2,3	P	7.8125	
Pyrimethamine	Undetermined		P	7.8125	
Isophorone diisocyanate	Undetermined		P	15.6250	
Cisplatin	TTC	1,2,3	P	16.9271	
Cobalt chloride	TTC	1,3	P	16.9271	
Chloroquine	TTC	1,3	P	17.8326	
Minocycline	TTC	3	P	18.5185	
Mitomycin C	Undetermined		P	20.0000	
Hydrogen peroxide	TTC	3	P	23.4375	
Citral	Undetermined	1	P	25.0000	
Dexamethasone	TTC	1,3	P	41.1692	
Pentamidine isethionate	TTC	3	P	52.0833	
Lead(ll)acetate	TTC	1,3	P	57.2917	
Azathioprine	TTC	1,2,3	P	58.4778	
Diesel exhaust particle	TTC	1,3	P	62.5000	
Sodium dodecyl sulfate	TTC	3	P	62.5000	
Dapsone	TTC	3	P	72.9167	
Nitrofurazone	NTTC		P	83.3333	
p-Nitroaniline	TTC	1,3	P	83.3333	
Sulfasalazine	TTC	1,3	P	92.9444	
Aluminium chloride	TTC	1,3	P	104.1667	
Nickel sulfate	TTC	1,3	P	104.1667	
Hydrocortisone	TTC	1,3	P	125	
Diethanolamine	Undetermined	1	P	250.0000	
Chloroplatinicacid	Undetermined		P	250.0000	
Sodium bromate	Undetermined	1	P	500.0000	
Histamine	TTC	3	P	750.0000	
Isoniazid	NTTC	1	N		
Triethanolamine	Undetermined		N		
Magnesium sulfate	Undetermined		N		
Rapamycin	TTC	1,3	N		
Mizoribine	Undetermined		N		
Warfarin	TTC	3	N		
2,4-Diaminotoluene	NTTC		N		
Cyclophosphamide	TTC	1	N*		
Dibenzopyrene	Undetermined		N		
Ethanol	TTC	1, 3	N		
Hexachloro benzene	Undetermined		N		
Lithium carbonate	TTC	1,3	N		
Methanol	NTTC		N		
Methotrexate	TTC	3	N		
Dimethyl sulfoxide	NTTC		N		
Trichloroethylene	NTTC		N		
Mycophenolicacid	Undetermined		P		0.395061728
2-Mercaptobenzothiazole	Undetermined		P		16.11328125
Ribavirin	TTC	1,3	P		26.04166667
Nicotinamide	Undetermined		P		288.0658436
Acetaminophen	Undetermined		P		288.0658436

TTC: Immunotoxic chemicals targeting T cells, NTTC (Others), Undetermined: Undetermined because of insufficient or inconsistent reported data, P: Positive, N: No effect, Blue color: accurate, Red color: false, yellow color: cannot be judged by undetermined classification of chemicals.

#: The criterion number used to define immunotoxicity.

*: Cyclophosphamide needs metabolic activity to demonstrate the activity.
