# Supplementary material for: An international validation study of the IL-2 Luc assay for evaluating the potential immunotoxic effects of chemicals on T cells and a proposal for reference data for immunotoxic chemicals
Source: Toxicol In Vitro. Author manuscript; Available in PMC 2022 Oct 11. (PMC9552337; doi:10.1016/j.tiv.2020.104832)
Supplement: 1 [file NIHMS1660497-supplement-1.docx]

Appendix 1. Multi-ImmunoTox Assay protocol ver. 011E

May. 10th, 2018

Department of Dermatology, Tohoku University Graduate School of Medicine

Yutaka Kimura, M.D., Ph.D.

Setsuya Aiba, M.D., Ph.D.

1. Introduction 5

2. Materials 6

2-1 Cells 6

2-2 Reagents and equipment 6

2-2-1 For maintenance of the 2H4 cells 6

2-2-2 For chemical exposure, stimulation and solvents 6

2-2-3 For measurement of the luciferase activity 6

2-2-4 Expendable supplies 6

2-2-5 Equipment for measurement of luciferase activity 7

2-2-6　Others 7

2-3 Culture medium 8

2-3-1 A medium: for maintenance of 2H4 cells (500 mL, stored at 2-8°C) 8

2-3-2 B medium: for luciferase assay (30 mL, stored at 2-8°C) 8

2-3-3 C medium: for thawing 2H4 cells (30 mL, stored at 2-8°C) 8

2-4 Preparation of the stimulant of 2H4 9

2-4-1 Phorbol 12-myristate 13-acetate (PMA) 9

2-4-2 Ionomycin 9

3. Cell culture 10

3-1 Thawing of 2H4 cells 10

3-2 Maintenance of 2H4 cells 10

4. Preparation of cells for assay 11

5. Preparation of chemicals and cell treatment with chemicals 12

5-1 Dissolution by vehicle (cf. Figure 3) 12

5-2 When the chemical is prepared in distilled water 15

5-2-1 Arrangement of chemicals and vehicle 15

5-2-2 Serial dilution 15

5-2-3 2 step dilution 16

5-3 When the chemical is prepared as DMSO solution 18

5-3-1 Arrangement of chemicals and vehicle 18

5-3-2 Serial dilution 18

5-3-3 Dilution of DMSO solution with the B medium 19

5-3-4 2 step dilution 20

6. Preparation of the stimulant (PMA/ionomycin) and addition to 2H4 22

6-1 Material 22

6-2 Preparation of 100 μM PMA 22

6-3 Preparation of control and x10 PMA/ionomycin solution 22

6-4 Addition of PMA/ionomycin to 2H4 23

7. Control 24

7-1 Preparing control chemical (dexamethasone, cyclosporine A) 24

7-1-1 Preparing dexamethasone stock 24

7-1-2 Preparing cyclosporine A stock 24

7-2 Preparation of cells for assay 25

7-3 Arrangement of chemicals and vehicle 26

7-4 Dilution with the B medium 26

7-5 2 step dilution 27

7-6 Addition of PMA/ionomycin to 2H4 29

8. Calculation of the transmittance factors 30

8-1 Reagents 30

8-2 Preparation of luminescence reaction solution 30

8-3 Bioluminescence measurement 30

9. Measurement 34

10. Data analysis 37

11. Criteria 37

11-1 Acceptance criteria 37

11-2 Criterion 37

12. Update record 39

Appendix 1 Principle of measurement of luciferase activity 41

Appendix 2 Validation of reagents and equipment 43

# 1. Introduction

This protocol describes how to maintain the cells, how to prepare the test chemicals, and how to measure the luciferase activity of 2H4 cells transfected with 3 luciferase genes, stable luciferase green (SLG), stable luciferase orange (SLO) and stable luciferase red (SLR), under the control of IL-2, IFNγ and G3PDH promoters, respectively, for the Multi-ImmunoTox Assay.

(Kimura Y. et al. Evaluation of the Multi-ImmunoTox Assay composed of 3 human cytokine reporter cells by examining immunological effects of drugs *Toxicol in Vitro*, 28, 759-768, 2014)

Figure 1

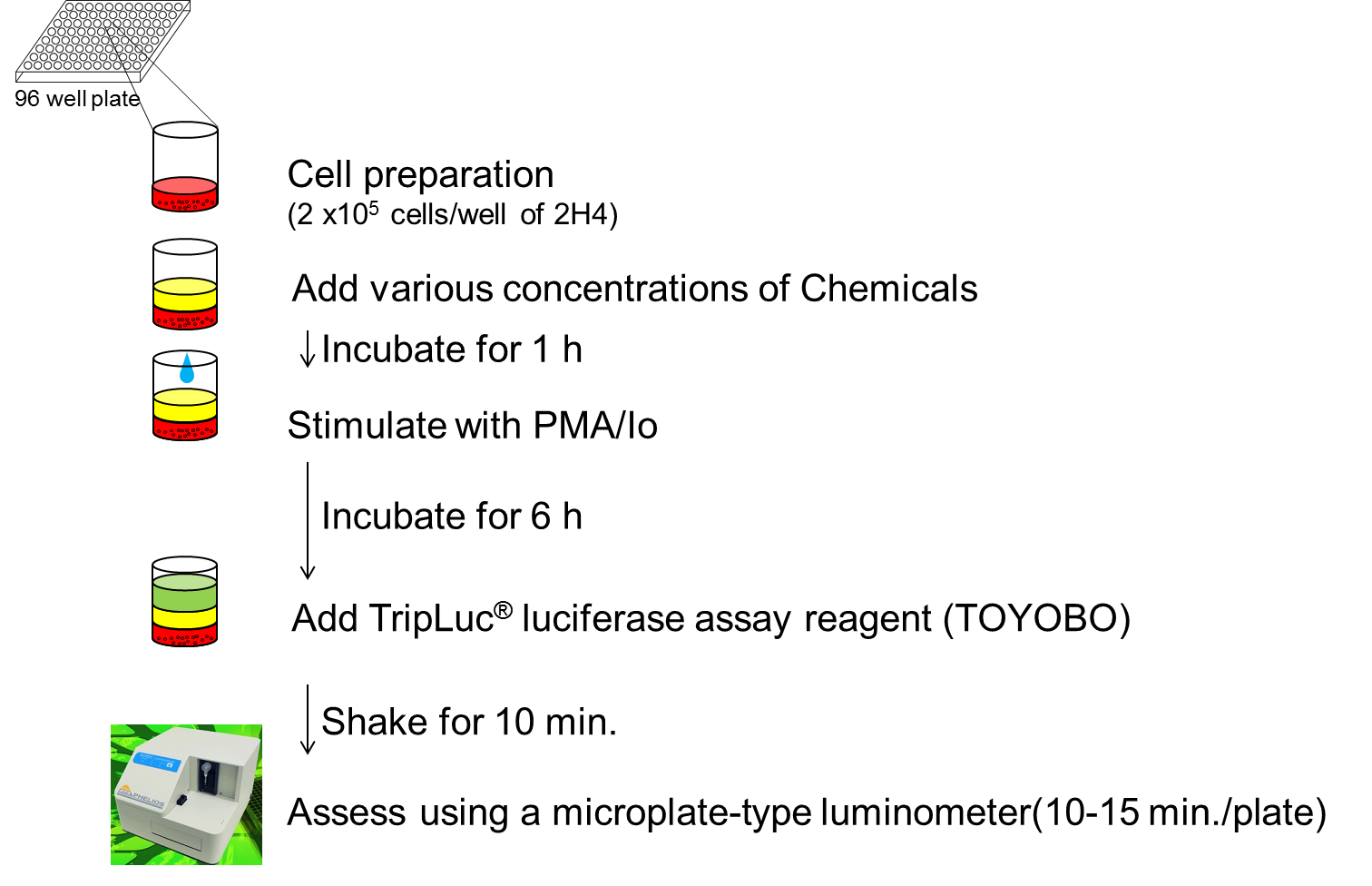


# 2. Materials

## 2-1 Cells

- 2H4 (IL2-SLG、IFNγ-SLO、G3PDH-SLR)

The human acute T lymphoblastic leukemia cell line Jurkat was obtained from the American Type Culture Collection (Manassas, VA, USA). A Jurkat-derived IL-2 and IFNγ reporter cell line, 2H4, that harbors the SLG, SLO and SLR luciferase genes under the control of the IL-2, IFNγ and GAPDH promoters, respectively, was established by Tsuruga Institute of Biotechnology, TOYOBO Co. Ltd.

(Saito R. et al. Nickel differentially regulates NFAT and NF-κB activation in T cell signaling *Toxicology and Applied Pharmacology,* 254, 245–255, 2011)

## 2-2 Reagents and equipment

### 2-2-1 For maintenance of the 2H4 cells

- RPMI-1640 (GIBCO Cat#11875-093, 500 mL)
- FBS (Biological Industries Cat#04-001-1E Lot: 715004)
- Antibiotic-Antimycotic (GIBCO Cat#15240-062)
- HygromycinB (CAS:31282-04-9, Invitrogen Cat#10687-010)
- G418 (CAS:108321-42-2, Nacalai Tesque Cat#16513-84)
- Puromycin (CAS:58-58-2, InvivoGen Cat#ant-pr-1)

### 2-2-2 For chemical exposure, stimulation and solvents

- Ionomycin (CAS:56092-82-1, Sigma Cat#I0634)
- Phorbol 12-myristate 13-acetate (PMA) (CAS:16561-29-8, Sigma Cat#P8139)
- Ethanol (e.g., Wako Cat#057-00456)
- Dimethyl sulfoxide (DMSO) (CAS:67-68-5, Sigma Cat#D5879)
- Distilled water (GIBCO Cat#10977-015)

### 2-2-3 For measurement of the luciferase activity

- Tripluc^®^ Luciferase assay reagent (TOYOBO Cat#MRA-301)

### 2-2-4 Expendable supplies

- T-75 flask tissue culture treated (e.g., Corning Cat#353136)
- 96 well μclear black plate (flat-bottom, for measurement of the luciferase activity, e.g. Greiner Bio-one Cat#655090)
- 96 well clear plate (round-bottom, for preparation of chemicals and stimulants)
- 96 well assay block, 2 mL (e.g., Costar Cat#3960)
- Seal for 96 well plate (e.g., Perkin Elmer TopSeal-A PLUS Cat#6050185, EXCEL Scientific SealMate Cat#SM-KIT-SP)
- Reservoir
- Pipette

### 2-2-5 Equipment for measurement of luciferase activity

- Measuring device: a microplate-type luminometer with a multi-color detection system that can accept two optical filter

e.g. Phelios AB-2350 (ATTO), ARVO (PerkinElmer), Tristar LB941 (Berthold)

- Optical filter: 560 nm long-pass filter and 600 nm long-pass filter
- Measuring time: set at 1～5 sec/well measuring time

### 2-2-6　Others

- Pipetman
- 8 channel or 12 channel pipetman (optimized for 10~100 μL)
- Plate shaker (for 96 well plate)
- CO_2_ incubator (37°C, 5% CO_2_)
- Water bath
- Cell counter: hemocytometer, trypan blue

## 2-3 Culture medium

### 2-3-1 A medium: for maintenance of 2H4 cells (500 mL, stored at 2-8°C)

| Reagent | Company | Concentration | Final concentration in medium | Required  amount |
| --- | --- | --- | --- | --- |
| RPMI-1640 | GIBCO #11875-093 | - | - | 440 mL |
| FBS | Biological Industries Cat#04-001-1E  Lot: 715004 | - | 10 % | 50 mL |
| Antibiotic-Antimycotic | GIBCO #15240-062 | 100× | 1× | 5 mL |
| Puromycin | InvivoGen # ant-pr-1 | 10 mg/mL | 0.15 μg/mL | 7.5 μL |
| G418 | Nacalai Tesque #16513-84 | 50 mg/mL | 300 μg/mL | 3 mL |
| HygromycinB | Invitrogen #10687-010 | 50 mg/mL | 200 μg/mL | 2 mL |

### 2-3-2 B medium: for luciferase assay (30 mL, stored at 2-8°C)

| Reagent | Company | Concentration | Final concentration in medium | Required  amount |
| --- | --- | --- | --- | --- |
| RPMI-1640 | GIBCO #11875-093 | - | - | 27 mL |
| FBS | Biological Industries Cat#04-001-1E  Lot: 715004 | - | 10 % | 3 mL |

### 2-3-3 C medium: for thawing 2H4 cells (30 mL, stored at 2-8°C)

| Reagent | Company | Concentration | Final concentration in medium | Required  amount |
| --- | --- | --- | --- | --- |
| RPMI-1640 | GIBCO #11875-093 | - | - | 26.7 mL |
| FBS | Biological Industries Cat#04-001-1E  Lot: 715004 | - | 10 % | 3 mL |
| Antibiotic-Antimycotic | GIBCO #15240-062 | 100× | 1× | 0.3 mL |

## 2-4 Preparation of the stimulant of 2H4

### 2-4-1 Phorbol 12-myristate 13-acetate (PMA)

| Reagent | Company | Concentration of the stock solution | Final concentration |
| --- | --- | --- | --- |
| Phorbol 12-myristate 13-acetate (PMA) | Sigma #P8139 | 2 mM | 25 nM |
| DMSO | Sigma #D5789 |  |  |

Dissolve 1 mg PMA using DMSO 811 μL, dispend at 5 μL/tube and store at freezer at -30°C. Use these stocks within 6 month after dissolution.

### 2-4-2 Ionomycin

| Reagent | Company | Concentration of the stock solution | Final concentration |
| --- | --- | --- | --- |
| Ionomycin | Sigma # I0634 | 2 mM | 1 μM |
| Ethanol | Wako #057-00456 |  |  |

Dissolve 1mg Ionomycin using ethanol 669.3 μL, dispend at 30 μL/tube and store at freezer at -30°C. Use these stocks within 6 month after dissolution.

# 3. Cell culture

## 3-1 Thawing of 2H4 cells

Pre-warm 9 mL of C medium in a 15 mL polypropylene conical tube in a 37°C water bath (for centrifugation) and 15 mL of C medium in a T-75 Flask at 37°C in a 5% CO_2_ incubator (for culture).

Thaw frozen cells (2x10^6^ cells / 0.5 mL of freezing medium) in a 37°C water bath, then add to a 15 mL polypropylene conical tube containing 9 mL of pre-warmed C medium. Centrifuge the tube at 120-350 x *g* at room temperature for 5 min, discard the supernatant, and resuspend in 15 mL of pre-warmed C medium in a T-75 Flask. Cells are incubated at 37°C, 5% CO_2_.

## 3-2 Maintenance of 2H4 cells

Pre-warm the A medium in a T-75 Flask at 37°C in a 5% CO_2_ incubator. The culture medium should be changed to the A medium 3 or 4 days after thawing. At that time, count the number of cells, centrifuge the tube at 120-350 x *g* at room temperature for 5 min, discard the supernatant, and resuspend in pre-warmed the A medium in a T-75 Flask. Cells are passaged at 3x10^5^/mL and incubated at 37°C, 5% CO_2_.

The interval between subcultures should be 3~4 days. Cells can be used between one and six weeks after thawing.

# 4. Preparation of cells for assay

A cell passage should be done 2-4 days before the assay.

Use cells between 1 and 6 weeks after thawing.

Pre-warm the B medium in a 37°C water bath. Count the number of cells and collect the number of cells needed (2.0 x 10^7^ cells for two chemicals are required, but to have some leeway, 3.0 x 10^7^ cells for two chemicals should be prepared), centrifuge the tube at 120-350 x *g*, 5 min. Resuspend in pre-warmed the B medium at a cell density of 4×10^6^/mL. Transfer the cell suspension to a reservoir, and add 50 μL of cell suspension to each well of a 96 well μclear black plate (flat bottom) using an 8 channel or 12 channel pipetman. (cf. Figure 2)

Figure 2

# 5. Preparation of chemicals and cell treatment with chemicals

## 5-1 Dissolution by vehicle (cf. Figure 3)

　Dissolve the chemical first in distilled water. Namely, weigh 0.025 g of the test chemical in a volumetric flask and add distilled water up to 1 mL. If the chemical is soluble at 25 mg/mL, weigh 0.050 g of the test chemical in a volumetric flask and add distilled water up to 1 mL. If the chemical is not soluble at 50 mg/mL, 25 mg/mL is the highest soluble concentration. If the chemical is soluble at 50 mg/mL, weigh 0.100 g of the test chemical in a volumetric flask and add distilled water up to 1 mL. If the chemical is not soluble at 100 mg/mL, 50 mg/mL is the highest soluble concentration. If the chemical is soluble at 100 mg/mL, 100 mg/mL is the highest soluble concentration.

If the chemical is not soluble in water, the chemical should be dissolved in DMSO at 500 mg/mL. Namely, weigh 0.5 g of the test chemical in volumetric flask and add DMSO up to 1 mL.

If the chemical is not soluble at 500 mg/mL, the highest soluble concentration should be determined by diluting the solution from 500 mg/mL at a common ratio of two (250 mg/mL 🡪 125 mg/mL 🡪 continued if needed) with DMSO.

Sonication and vortex may be used if needed，and attempt to dissolve the chemical for at least 5 minutes. Being soluble should be confirmed by centrifugation at 15,000 rpm (≈20,000 x g) for 5 min and absence of precipitation. The chemical should be used within 4 hours after being dissolved in distilled water or DMSO.

Figure 3


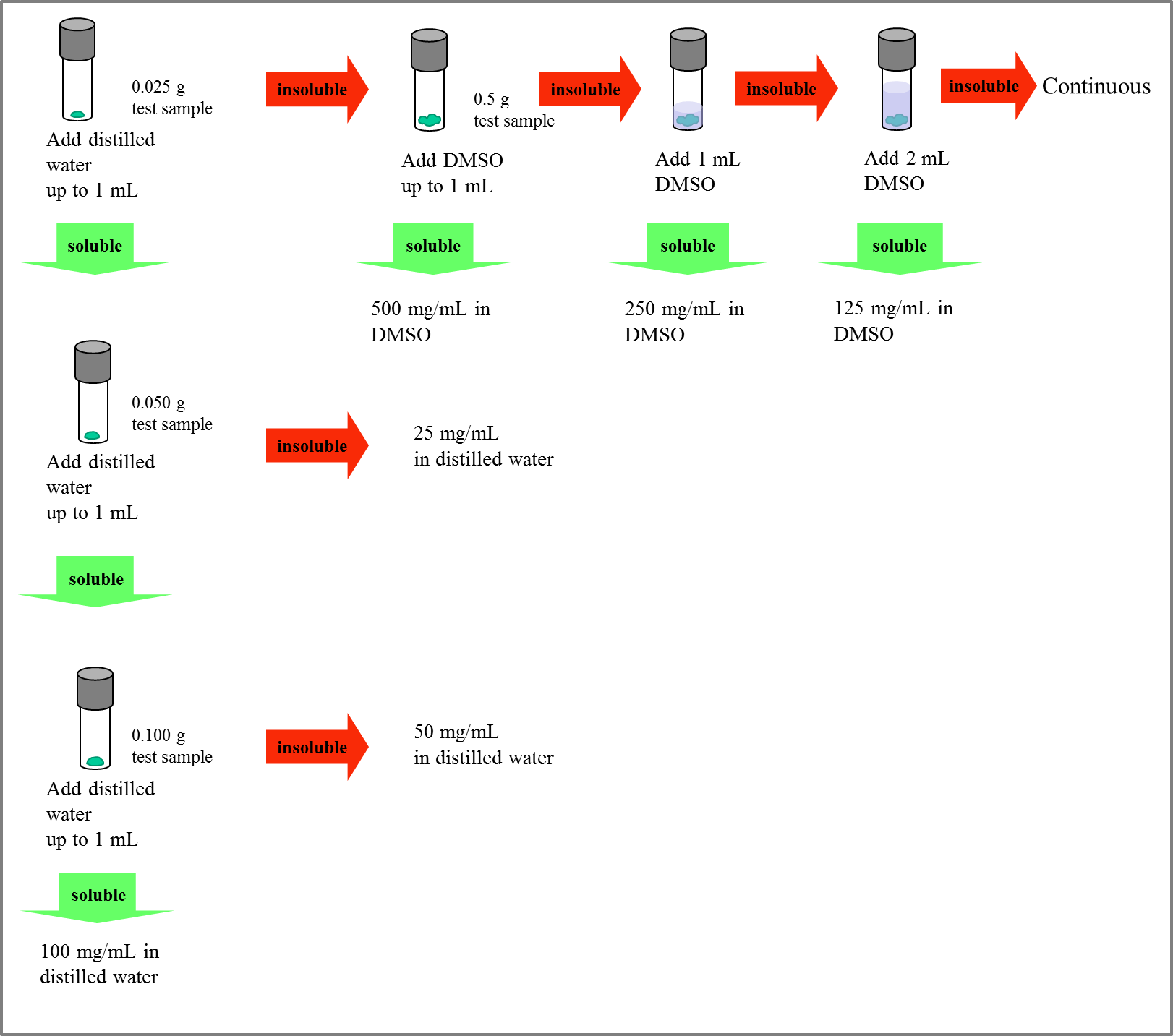


In the first experiment (1^st^ experiment), when the chemical is prepared in distilled water, conduct 10 serial dilutions at a common ratio of 2 from the highest concentration using distilled water. When the chemical is prepared as a DMSO solution, conduct 10 serial dilutions at a common ratio of 2 from the highest concentration using DMSO.

In the second to fourth experiment (2^nd^ to 4^th^ experiment), determine the minimum concentration at which I.I.-SLR-LA (mentioned later in **10**) became lower than 0.05 in the 1^st^ experiment, use the concentration one step (2-times) higher than this determined concentration as the highest concentration of the chemical to examine, and conduct 10 serial dilutions at a common ratio of 2 from the highest concentration. If I.I.-SLR-LA did not become lower than 0.05 or became lower than 0.05 at the highest concentration in the 1^st^ experiment, conduct 10 serial dilutions at a common ratio of 2 from the highest concentration in the 1^st^ experiment.

For example, in Figure 3 below, the minimum concentration at which I.I.-SLR-LA became lower than 0.05 is 1.95 μg/ml. The highest concentration of the chemical to examine is the concentration one step (2-times) higher than 1.95 μg/ml, which is 3.91 μg/ml.

In Figure 4 below, I.I.-SLR-LA did not become lower than 0.05. In such a case, the highest concentration of the chemical to examine is the highest concentration in the 1^st^ experiment, namely 125 μg/ml.

Figure 3.

Figure 4

## 5-2 When the chemical is prepared in distilled water

If the chemical is prepared at a lower concentration, use the prepared concentration instead of the 100 mg/mL distilled water solution.

### 5-2-1 Arrangement of chemicals and vehicle

Add 100 μL of the 100 mg/mL distilled water solution of the chemical to well #A12, and 50 μL of the distilled water to wells #A1-#A11 of the 96 well clear plate (round bottom).

### 5-2-2 Serial dilution

Conduct 9 serial dilutions at a common ratio of 2 as indicated in Figure 4 from well #A11 to well #A3. Transfer 50 μL to the next (left) well. (cf. Figure 4)

Figure 4

### 5-2-3 2 step dilution

Add 20 μL of the diluted chemical to 480 μL of the B medium prepared in the assay block. And add 50 μL to 2H4 in a 96 well plate using an 8 channel or 12 channel pipetman after pipetting 20 times. Seal the plate, shake the plate with a plateshaker and incubate in a CO_2_ incubator for 1 hour (37°C, CO_2_, 5%) (cf. Figure 5-7).

Figure 5

Figure 6

Figure 7 Final constituents of each well of the plate

## 5-3 When the chemical is prepared as a DMSO solution

If the chemical is prepared at a lower concentration, use the prepared concentration instead of 500 mg/mL DMSO solution.

### 5-3-1 Arrangement of chemicals and vehicle

Add 100 μL of the 500 mg/mL DMSO solution of the chemical to well #A12, 50 μL of DMSO to wells #A1-#A11, and 90 μL of the B medium to wells #B1-#B12 of the 96 well clear plate (round bottom)

### 5-3-2 Serial dilution

Conduct 9 serial dilutions at a common ratio of 2 as indicated in Figure 8 from well #A11 to well #A3. Transfer 50 μL to the next (left) well. (cf. Figure 8)

Figure 8

### 5-3-3 Dilution of DMSO solution with the B medium

Dilute 10 μL of the DMSO solution of the chemical in wells #A1-#A12 with 90 μL of the B medium using an 8-12 channel pipetman. (cf. Figure 9)

Figure 9

### 5-3-4 2 step dilution

Add 10 μL of the diluted chemical to 490 μL of the B medium prepared in the assay block. And add 50 μL to 2H4 in a 96 well plate using an 8 channel or 12 channel pipetman after pipetting 20 times. Manipulate the procedures from 5-3-3 to 5-3-4 as quickly as you can, and do not leave a long time at step after 5-3-3 or Figure 10. Seal the plate, shake the plate with a plateshaker and incubate in a CO_2_ incubator for 1 hour (37°C, CO_2_, 5%) (cf. Figure 10-12).

Figure 10

Figure 11

Figure 12 Final constituents of each well of the plate

# 6. Preparation of the stimulant (PMA/ionomycin) and addition to 2H4

## 6-1 Material

・2 mM PMA stock

・2 mM Ionomycin stock

・B medium

・Ethanol

## 6-2 Preparation of 100 μM PMA

Dilute 2 mM PMA stock with the B medium as follows (20 times, final concentration is 100 μM).

| 2 mM PMA | B medium | Total | final concentration |
| --- | --- | --- | --- |
| 5 μL | 95 μL | 100 μL | 100 μM |

## 6-3 Preparation of control and x10 PMA/ionomycin solution

Dilute ethanol, 2 mM ionomycin and 100 μM PMA with the B medium to prepare control or x10 PMA/ionomycin solution. Add the control to well #A1-#H1 of the 96 well clear plate (round bottom), and add x10 PMA/ionomycin solution to wells #A2-#H2 of the 96 well clear plate (round bottom).

|  | B medium | 2 mM Ionomycin | 100 μM PMA | Ethanol | Total |
| --- | --- | --- | --- | --- | --- |
| Control | 995 μL | - |  | 5 μL | 1000 μL |
| x10 PMA/ionomycin solution | 2382 μL | 12 μL | 6 μL | - | 2400 μL |

## 6-4 Addition of PMA/ionomycin to 2H4

One hour after the addition of chemicals, add 10 μL of control or PMA/ionomycin solution to the cells (#A1-#H1 or #A2-#H12, respectively) using an 8 channel or 12 channel pipetman after pipetting 20 times. Make sure that the apex of the tip is dipped into the medium. Change tips every line you add. Seal the plate, shake the plate with a plateshaker and incubate in a CO_2_ incubator for 6 hour (37°C, CO_2_, 5%). (cf. Figure 13)

Figure 13

# 7. Control

## 7-1 Preparing control chemical (dexamethasone, cyclosporine A)

### 7-1-1 Preparing dexamethasone stock

| Reagent | Company | Concentration of the stock solution | Preparing concentration | Final concentration |
| --- | --- | --- | --- | --- |
| Dexamethasone-water soluble | Sigma #D2915-100MG | 2.5 mg/mL | 2.5 mg/mL | 50 μg/mL |
| Distilled water | GIBCO Cat#10977-015 |  |  |  |

　Dissolve 100 mg of Dexamethasone-water soluble with distilled water 40 mL, dispend at 50 μL/tube and store a freezer at -30°C.

### 7-1-2 Preparing cyclosporine A stock

| Reagent | Company | Concentration of the stock solution | Preparing concentration | Final concentration |
| --- | --- | --- | --- | --- |
| Cyclosporine A | Sigma #C1832-5MG | 100 μg/mL | 100 μg/mL | 100 μg/mL |
| DMSO | Sigma #D5789 |  |  |  |

Dissolve 5 mg of cyclosporine A with DMSO 50 mL, dispend at 50 μL/tube and store a freezer at -30°C.

## 7-2 Preparation of cells for assay

A cell passage should be done 2-4 days before the assay.

Use cells between 1 and 6 weeks after thawing.

Pre-warm the B medium in a 37°C water bath. Count the number of cells and collect the number of cells needed (5.0 x 10^6^ cells are required, but to have some leeway, 7.5 x 10^6^ cells should be prepared), centrifuge the tube at 120-350 x *g*, 5 min. Resuspend in pre-warmed the B medium at a cell density of 4×10^6^/mL. Transfer the cell suspension to a reservoir, and add 50 μL of cell suspension to each well of a 96 well μclear black plate (flat bottom) using an 8 channel or 12 channel pipetman. (cf. Figure 14)

Figure 14

## 7-3 Arrangement of chemicals and vehicle

Add DMSO 50 μL to #A4, 100 μg/mL cyclosporine A stock 50 μl to #A5, distilled water 50 μl to #B1 and #B2, 2.5 mg/ml dexamethasone stock 50 μl to #B3 and the B medium 180 μl to #B4 and #B5 of the 96 well clear plate (round bottom). (cf. Figure 15)

## 7-4 Dilution with the B medium

Dilute DMSO in #A4 and cyclosporine A DMSO solution in #A5 by adding 20 μL to the B medium in #B4 and #B5, respectively. (cf. Figure 15)

Figure 15

## 7-5 2 step dilution

Add 20 μL of the diluted chemical or vehicle to 480 μL (1-3 lanes) or 980 μL (4-5 lanes) of the B medium prepared in the assay block. And add 50 μL to 2H4 in a 96 well plate using an 8 channel or 12 channel pipetman after pipetting 20 times. Manipulate the procedures from 7-4 to 7-5 as quickly as you can, and do not leave a long time at step after 7-4 or Figure 16. Seal the plate, shake the plate with a plateshaker and incubate in a CO_2_ incubator for 1 hour (37°C, CO_2_, 5%). (cf. Figure 16-18)

Figure 16

Figure 17

Figure 18 Final constituents of each well of the plate

## 7-6 Addition of PMA/ionomycin to 2H4

One hour after the addition of dexamethasone and cyclosporine A, add 10 μL of control or PMA/ionomycin solution prepared in §6-3 to the cells (#A1-#D1 or #A2-#D5, respectively) using an 8 channel or 12 channel pipetman after pipetting 20 times. Make sure that the apex of the tip is dipped into the medium. Change tips every line you add. Seal the plate, shake the plate with a plateshaker and incubate in a CO_2_ incubator for 6 hour (37°C, CO_2_, 5%). (cf. Figure 19)

Figure 19

# 8. Calculation of the transmittance factors

Color discrimination in multi-color reporter assays is generally achieved using detectors (luminometer and plate reader) equipped with optical filters, such as sharp-cut (long-pass) filters and band-pass filters. The transmittance factors of these filters for each bio-luminescence signal color must be calibrated prior to all experiments by following the protocols below.

## 8-1 Reagents

- Single reference samples:

Lyophilized luciferase enzyme reagent for stable luciferase green (SLG)

Lyophilized luciferase enzyme reagent for stable luciferase orange (SLO)

Lyophilized luciferase enzyme reagent for stable luciferase red (SLR)

- Assay reagent:

Tripluc^®^ Luciferase assay reagent（TOYOBO Cat#MRA-301）

- B medium: for luciferase assay (30 mL, stored at 2- 8°C)

| Reagent | Company | Conc. | Final conc. in medium | Required amount |
| --- | --- | --- | --- | --- |
| RPMI-1640 | GIBCO #11875-093 | - | - | 27 mL |
| FBS | Biological Industries Cat#04-001-1E  Lot: 715004 | - | 10 % | 3 mL |

## 8-2 Preparation of luminescence reaction solution

Thaw Tripluc^®^ Luciferase assay reagent (Tripluc) and keep it at room temperature either in a water bath or at ambient air temperature. Power on the luminometer 30 min before starting the measurement to allow the photomultiplier to stabilize.

Add 200 μL of 100 mM Tris-HCl (pH8.0) contains 10 % glycerol to each tube of lyophilized reference sample to dissolve the enzymes, divide into 10 μL aliquots in 1.5 mL disposable tubes and store in a freezer at -80°C. The stored frozen solution of the reference samples can be used for up to 6 months.

Add 1 mL of the B medium to each tube of frozen reference sample (10 μL sample per tube). Keep the reference samples on ice to prevent deactivation.

## 8-3 Bioluminescence measurement

Transfer 100 μL of the diluted reference samples to a black 96 well plate (flat bottom) as shown below.

Figure 20

Transfer 100 μL of pre-warmed Tripluc to each well of the plate containing the reference sample using a pipetman. Shake the plate for 10 min at room temperature (about 25°C) using a plate shaker. Remove bubbles in the solutions in wells if they appear. Place the plate in the luminometer to measure the luciferase activity. Bioluminescence is measured for 3 sec each in the absence (F0) and presence (F1, F2) of the optical filters. An example of the raw output data is shown below.

Figure 22

Six transmittance factors of the optical filters were calculated as follow:

Transmittance factor (κG_R56_)=$\frac{\#B1 of F1+ \#B2 of F1+ \#B3 of F1}{\#B1 of F0+ \#B2 of F0+ \#B3 of F0}$

Transmittance factor (κO_R56_)=$\frac{\#D1 of F1+\#D2 of F1+\#D3 of F1}{\#D1 of F0+\#D2 of F0+\#D3 of F0}$

Transmittance factor (κR_R56_)= $\frac{\#F1 of F1+\#F2 of F1+\#F3 of F1}{\#F1 of F0+\#F2 of F0+\#F3 of F0}$

Transmittance factor (κG_R60_)=$\frac{\#B1 of F2+ \#B2 of F2+ \#B3 of F2}{\#B1 of F0+ \#B2 of F0+ \#B3 of F0}$

Transmittance factor (κO_R60_)=$\frac{\#D1 of F2+\#D2 of F2+\#D3 of F2}{\#D1 of F0+\#D2 of F0+\#D3 of F0}$

Transmittance factor (κR_R60_)=$\frac{\#F1 of F2+\#F2 of F2+\#F3 of F2}{\#F1 of F0+\#F2 of F0+\#F3 of F0}$

In the case shown above,

Transmittance factors (κG_R56_)=$\frac{1269550+1257268+1289562}{3757015+3716611+3810382}$=0.338

Transmittance factors (κO_R56_)=$\frac{808550+813160+754174}{1202691+1210208+1122295}$=0.672

Transmittance factors (κR_R56_)=$\frac{2193723+1968240+1853873}{2465453+2207572+2077689}$=0.891

Transmittance factors (κG_R60_)=$\frac{236478+234079+240876}{3757015+3716611+3810382}$=0.06

Transmittance factors (κO_R60_)=$\frac{235121+235878+217432}{1202691+1210208+1122295}$=0.195

Transmittance factors (κR_R60_)=$\frac{1585258+1420099+1339265}{2465453+2207572+2077689}$=0.644

Calculated transmittance factors are used for all the measurements executed using the same luminometer.

Input the transmittance factors to #C6-#E7 of the “Data Input” sheet of the Data sheet as follow.

Figure 23


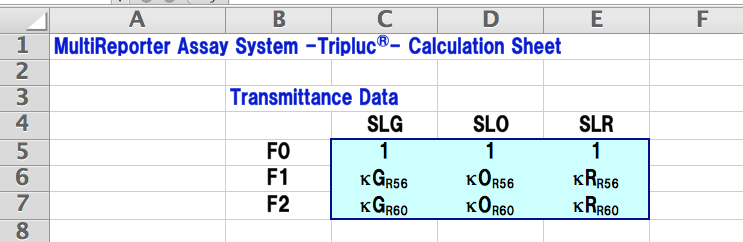


# 9. Measurement

Please refer Appendix 1 for the principle of measurement of luciferase activity.

Thaw Tripluc^®^ Luciferase assay reagent (Tripluc) and keep it at room temperature either in a water bath or at ambient air temperature. Power on the luminometer 30 min before starting the measurement to allow the photomultiplier to stabilize.

Transfer 100 μL of pre-warmed Tripluc from the reservoir to each well of the plate containing the reference sample using an 8 channel or 12 channel pipetman. Shake the plate for 10 min at room temperature (about 25°C) on a plate shaker. Remove bubbles in the solutions in the wells if they appear. Place the plate in the luminometer to measure the luciferase activity. Bioluminescence is measured for 3 sec each in the absence of (F0) and presence (F1, F2) of the optical filters.

1^st^. Add the information regarding the name of laboratory, the round of experiments if multiple experimental sets are performed, the experiment number, date, the operator, chemical codes, dissolved in distilled water or DMSO, the prepared concentration, molecular weight of the chemicals and comments if any to Face Sheet of the data sheet.

Figure 24 “Face Sheet” of the data sheet

2^nd^. Copy the results of the F0, F1 and F2 measurements (values are expressed as counts) and paste them into the appropriate area in the “Data Input” sheet of the data sheet shown below. In addition, input the transmittance factors calculated in "§5. Calculation of the transmittance factors" to #C6-#E7 of the “Data Input” sheet.

Figure 25 “Data Input” sheet of the data sheet


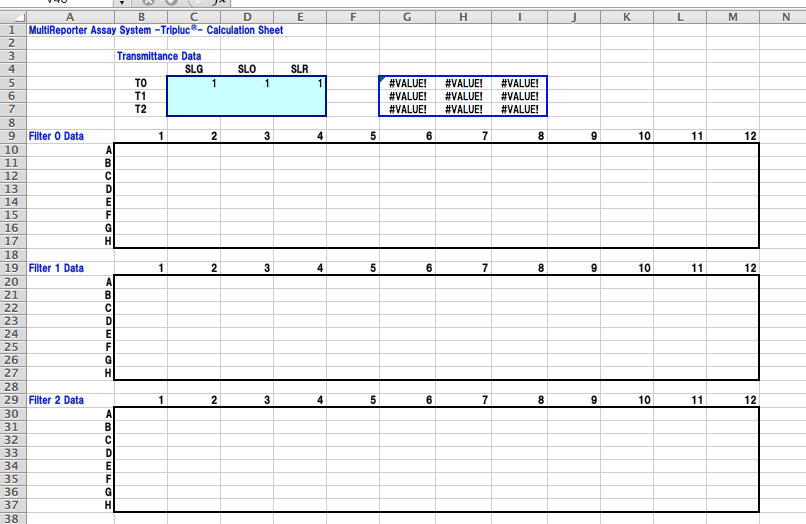


Next, the calculated results for the parameters of the Multi-Immuno Tox assay for each concentration, e.g., SLG-LA, SLO-LA, SLR-LA, nSLG-LA, nSLO-LA, the mean ± SD of SLG-LA, the mean ± SD of SLO-LA, the mean ± SD of SLR-LA %suppression and graphs will automatically appear on the “Result Format” sheet of the data sheet.

Figure 26 “Result Format” sheet of the data sheet

# 10. Data analysis

Definition of the parameters used in the Multi-Immuno Tox assay

- SLG-luciferase activity (SLG-LA)：Luciferase activity of stable luciferase green

(Under the control of IL-2 promoter)

- SLO-luciferase activity (SLO-LA)：Luciferase activity of stable luciferase orange

(Under the control of IFN-γ promoter)

- SLR-luciferase activity (SLR-LA)：Luciferase activity of stable luciferase red

(Under the control of G3PDH promoter)

- Normalized SLG-LA (nSLG-LA)：=(SLG-LA)/(SLR-LA)
- Normalized SLO-LA (nSLO-LA)：=(SLO-LA)/(SLR-LA)
- Inhibition index of SLR-LA (I.I.-SLR-LA)：The cytotoxic effect of chemicals

　　=(SLR-LA of 2H4 treated with chemicals)/(SLR-LA of untreated 2H4)

- %suppression：The effect of chemicals on IL-2 or IFN-γ promoter

=(1-(nSLG-LA or nSLO-LA of 2H4 treated with chemicals)

/(nSLG-LA or nSLO-LA of non-treated 2H4)) x 100

# 11. Criteria

## 11-1 Acceptance criteria

The following acceptance criteria should be satisfied when using the Multi-Immuno Tox Assay method.

- If Fold induction of nSLO-LA of PMA/Ionomycin wells without chemicals (=(nSLO-LA of 2H4 cells treated with PMA/Ionomycin) / (nSLO-LA of non-treated 2H4 cells)) demonstrate less than 3.0, the results obtained from the plate containing the control wells should be rejected.

## 11-2 Criterion

The experiments are repeated until two consistent positive (negative) results or two consistent “no effect results” are obtained. When two consistent results are obtained, the chemicals are judged as the obtained consistent results.

Identification of immunotoxicant is evaluated by the mean of %suppression and its 95% simultaneous confidence interval.

In each experiment, when the chemicals clear the following 3 criteria, they are judged as suppressive or stimulatory. Otherwise, they are judged as no effect chemicals.

1. The mean of %suppression is > 35 (suppressive) or < -35 (stimulatory) with statistical significance. The statistical significance is judged by its 95% confidence interval.

2. The result shows two or more consecutive statistically significant positive (negative) data or one statistically significant positive (negative) data with a trend in which at least 3 consecutive data increase (decrease) in a dose dependent manner. In the latter case, the trend can cross 0, as long as only one data point shows the opposite effect without statistical significance.

3. The results are judged using only data obtained in the concentration at which I.I.-SLR-LA is > 0.05

# 12. Update record

Ver. 0011.0E 2018.5.10

Change the criteria

Ver. 0010.0E 2018.1.15 distribution

Change the criteria

Ver. 009.1E 2017.5.8 distribution

Change the criteria

Ver. 009.0E 2017.4.7 distribution

Change the preparation of chemicals

Change the acceptance criteria

Change the criteria

Ver. 008.5E 2016.9.14 distribution

Change the criteria

Ver. 008.4E 2016.9.9 distribution

Change the criteria

Ver. 008.3E 2016.8.1 distribution

Correction of the preparation of PMA and ionomycin

Change the preparation of PMA and ionomycin

Change the preparation of controls

Addition of Acceptance criteria

Ver. 008.1E 2016.2.2 distribution

Changes after the VMT meeting

Ver. 008.0E 2016.1.19

Translation to English

Addition of appendix

Ver. 006.0J 2015.8.17

Change the preparation of chemicals (same method to the IL-8 Luc assay)

Delete the alteration in Ver. 005.0J

Ver. 005.0J 2015.1.9　distribution

Change to use SLR-LA of THP-G8 at the calculation of nSLG-LA of TGCHAC-A4

Ver. 004.1J 2014.12.10　distribution

Change the cellar concentration at cell passage

Modify figure 16, 17

Ver. 004.0J 2014.11.17　distribution

For the validation study at AIST, FDSC and Tohoku university (chemicals: Sodium Bromate (NaBrO_3_), Nickel (II) sulfate (NiSO_4_), Dibutyl phthalate (DP), 2-Mercaptobenzothiazole (2-MBT))

Change THP-G1b cells to TGCHAC-A4 cells

Change cell number of THP-G8 and TGCHAC-A4 5x10^4/well to 1x10^5/well

Change concentration of chemicals 11 steps to 10 steps

Change final concentration of LPS (THP-G8 : 25 ng/mL, TGCHAC-A4 : 1 ng/mL)

Change the way of addition of LPS (2 mL/well to 10 mL/well)

Change the criteria

Ver. 002.0J 2013.08.19 distribution

For the validation study at AIST and FDSC (chemicals: CoCl_2_, NiSO_4_, Isophorone diisocyanate, 2-Mercaptobenzothiazole）

Change the common ratio 3 to 2

Change the concentration of LPS 100 ng/mL to 25 ng/mL

Add description about the control (dexamethasone)

Delete the appendix about THP-G8 cell

Ver. 001.1J 2012. Nov. 13 distribution

Add the appendix about THP-G8 cell

Ver. 001J 2012. Nov. 09 distribution

# Appendix 1 Principle of measurement of luciferase activity

MultiReporter Assay System -Tripluc- can be used with a microplate-type luminometer with a multi-color detection system, which can equip two optical filters (e.g. Phelios AB-2350 (ATTO), ARVO (PerkinElmer), Tristar LB941 (Berthold)). The optical filters used in measurement are a 560 nm long-pass filter and a 600 nm long-pass filter.

(1) Measurement of three-color luciferase with two optical filters.

This is an example using Phelios AB-2350 (ATTO). This luminometer equips a 560 nm long-pass filter (560 nm LP, Filter 1) and a 600 nm long pass filter (600 nm LP, Filter 2) for optical isolation.

First, using luciferase enzyme reagent of SLG (λmax = 550 nm), SLO (λmax = 580 nm) and SLR (λmax = 630 nm), measure i) the intensity of light without filter (all optical), ii) the intensity of 560 nm LP (Filter 1) transmitted light iii) the intensity of 600 nm LP (Filter 2) transmitted light, and calculate the coefficient factor listed below.

| Coefficient factor | | Abbreviation | Definition |
| --- | --- | --- | --- |
| SLG | Filter 1 transmittance factor | κG_R56_ | The intensity of 560 nm LP (Filter 1) transmitted SLG / the intensity of SLG without filter (all optical) |
|  | Filter 2 transmittance factor | κG_R60_ | The intensity of 600 nm LP (Filter 2) transmitted SLG / the intensity of SLG without filter (all optical) |
| SLO | Filter 1 transmittance factor | κO_R56_ | The intensity of 560 nm LP (Filter 1) transmitted SLO / the intensity of SLO without filter (all optical) |
|  | Filter 2 transmittance factor | κO_R60_ | The intensity of 600 nm LP (Filter 2) transmitted SLO / the intensity of SLO without filter (all optical) |
| SLR | Filter 1 transmittance factor | κR_R56_ | The intensity of 560 nm LP (Filter 1) transmitted SLR / the intensity of SLR without filter (all optical) |
|  | Filter 2 transmittance factor | κR_R60_ | The intensity of 600 nm LP (Filter 2) transmitted SLR / the intensity of SLR without filter (all optical) |

When the intensity of SLG, SLO and SLR in test sample are defined as G, O and R, respectively, i) the intensity of light without filter (all optical): F0, ii) the intensity of 560 nm LP (Filter 1) transmitted light and iii) the intensity of 600 nm LP (Filter 2) transmitted light are described as below.

F0=G+O+R

F1=κG_R56_ x G + κO_R56_ x O + κR_R56_ x R

F2=κG_R60_ x G + κO_R60_ x O + κR_R60_ x R

These formulas can be rephrased as follows

$$\left( \begin{aligned} F0 \\ F1 \\ F2 \end{aligned} \right)=\left( \begin{aligned} 1 1 1 \\ \kappa G_{R56} \kappa O_{R56} \kappa R_{R56} \\ \kappa G_{R60} \kappa O_{R60} \kappa R_{R60} \end{aligned} \right)\left( \begin{aligned} G \\ O \\ R \end{aligned} \right)$$

Then using calculated coefficient factors and measured F0, F1 and F2, you can calculate G, O and R-value as follows.

$$\left( \begin{aligned} G \\ O \\ R \end{aligned} \right)=\left( \begin{aligned} 1 1 1 \\ \kappa G_{R56} \kappa O_{R56} \kappa R_{R56} \\ \kappa G_{R60} \kappa O_{R60} \kappa R_{R60} \end{aligned} \right)^{-1}\left( \begin{aligned} F0 \\ F1 \\ F2 \end{aligned} \right)$$

This calculation can be performed using the functions "MININVERSE" and "MMULT" in Microsoft Excel. These calculations are integrated in the Data Sheet.

# Appendix 2 Validation of reagents and equipment

5-1 Measurement of transmittance of optical filter for multicolor measurement

For color discriminations in the multi-color reporter assay, detectors (luminometer and plate reader) are usually equipped with optical filters, such as sharp-cut (long-pass) filters and band-pass filters. The transmittance factors of these filters for each bioluminescence signal color have to be calibrated prior to all experiments by following the protocols below.

5-1-1 Reagents

・Single reference samples:

Lyophilized luciferase enzyme reagent of SLG

Lyophilized luciferase enzyme reagent of SLO

Lyophilized luciferase enzyme reagent of SLR

・Assay reagent:

Tripluc^®^ Luciferase assay reagent（TOYOBO Cat#MRA-301）

・B medium: for luciferase assay (30 mL, stored at 2 – 8°C)

| Reagent | Company | Conc. | Final conc. in medium | Required amount |
| --- | --- | --- | --- | --- |
| RPMI-1640 | GIBCO #11875-093 | - | - | 27 mL |
| FBS | Biological Industries Cat#04-001-1E  Lot: 715004 | - | 10 % | 3 mL |

5-1-2 Calibration

5-1-2-1 Preparation of luminescence reaction solution

Thaw Tripluc^®^ Luciferase assay reagent (Tripluc) and keep it at room temperature by bathing in water or ambient air. Start the luminometer 30 min before starting the measurement for stabilization of the photomultiplier.

Add 200 μL of 100 mM Tris-HCl (pH8.0) contains 10% glycerol to each tube of the lyophilized reference samples to dissolve the enzymes, followed by separating them into 1.5 mL disposable tubes at 10 μL each and storing in a freezer at -80°C. The stored frozen solution of the reference samples can be used for one half year.

Add 1 mL of the B medium to each tube of the frozen reference sample (10 μL in a tube) and label them as SLG1/1, SLO1/1 and SLR1/1. Keep the reference samples on ice to prevent deactivation.

Prepare dilution series of the single reference samples of SLG, SLO and SLR as follows. Dilute 0.3 mL of each 1/1 solution with 0.9 mL of the B medium to make SLG1/4, SLO1/4 and SLR1/4. In the same manner, prepare 1/16 and 1/64 solution of each. Keep diluted reference samples on ice.

5-1-2-2 Bioluminescence measurement

Transfer 100 μL of the diluted reference samples to a black 96 well plate (flat bottom) as shown below.

Figure 27

Transfer 100 μL of pre-warmed Tripluc to each well containing the reference samples of the plate using a pipetman. Shake the plate for 10 min at room temperature (about 25°C) with a plate shaker. Remove bubbles on the solutions in wells if they appear. Place the plate into the luminometer to measure the luciferase activity. Bioluminescence is measured for 3 sec each in the absence (F0) and presence (F1, F2) of the optical filters.

Copy the results of the F0, F1 and F2 measurement (values are expressed as counts) and paste it to the appropriate area in the “Data Input” sheet of the data sheet for data analyses shown below.

Figure 28


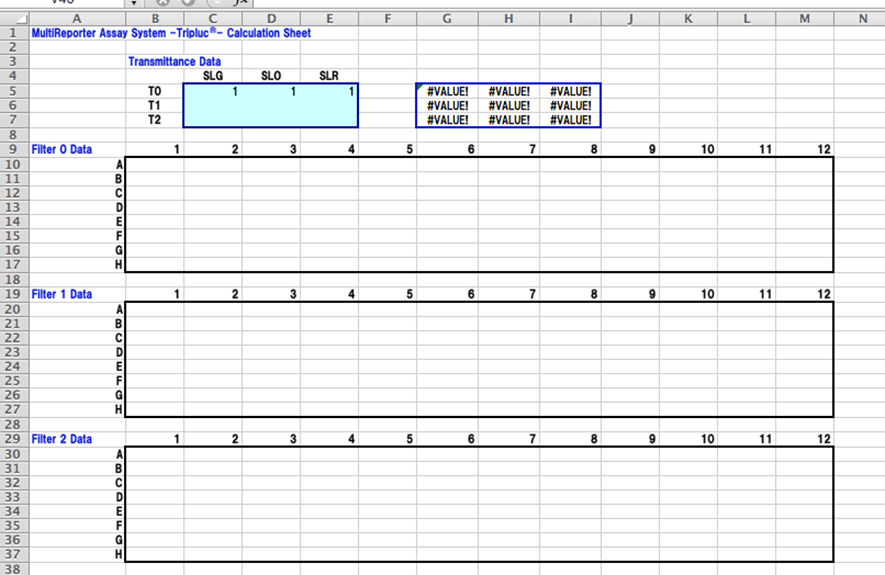
 Record all the results for quality control.

5-2 Quality control of equipment

In order to confirm the detector stability as the quality control, the reference luciferase sample, optical property, the protocol described here should be performed at the beginning of the experiments every day.

5-2-1 Light source

LED Plate: Reference LED light source plates equipped with stabilized red, green, and blue LEDs are commercially available. For example,

TRIANT® (wSL-0001) by ATTO (Tokyo, Japan)

L12367 by Hamamatsu Photonics (Shizuoka, Japan)

5-2-2 Data collection (an example using TRIANT® by ATTO)

1) Start luminometer 30 min before starting the measurement for stabilization of the photomultiplier.

2) Start LED plate and select “PMT” mode.

3) Select three-color (BRG) mode and adjust light intensity to 1/10 (10E-1).

4) Place the LED plate into the luminometer. Light intensity is measured for 3 sec each in the absence (F0) and presence (F2) of the optical filter.

5) Blue, green, and red LEDs are located at the position of #F6, #E6, and #D6, respectively. Copy the collected data of each position to the appropriate area on Sheet “LED” in the excel file of the data sheet.

6) Check the photo-detector performance by comparing with old data of the LED plate. For quality control purpose, every collected data should be recorded.

7) LED plate data typically fluctuates up to 1.5% (σ). Disagreement to the old data should be less than 3×σ (= 4.5%).
