# Supplementary material for: An international validation study of the IL-2 Luc assay for evaluating the potential immunotoxic effects of chemicals on T cells and a proposal for reference data for immunotoxic chemicals
Source: Toxicol In Vitro. Author manuscript; Available in PMC 2022 Oct 11. (PMC9552337; doi:10.1016/j.tiv.2020.104832)
Supplement: 2 [file NIHMS1660497-supplement-2.pdf]

Appendix 2. Immunotoxicological information of 25 chemicals used in the validation study

MITA Literature Reports

August 8, 2018

Table of Contents

2,4-Diaminotoluene (DAT) [CASRN 95-80-7] 1
5,5-Diphenylhydantoin (DPH) [CASRN 57-41-0] 3
Acetonitrile [CASRN 75-05-8] 7
Benzo(a)pyrene [B(a)P] [CASRN 50-32-8] 9
Cadmium Chloride [CASRN 10108-64-2] 14
Dibromoacetic Acid (DBAA) [CASRN 631-61-1] 17
Dibutyl phthalate (DBP) [CASRN 84-74-2] 21
Dichloroacetic Acid (DCAA) [CASRN 79-43-6] 25
Diethylstilbestrol (DES) [CASRN 56-53-1] 27
Ethylene Dibromide (EDB) [CASRN 106-93-4] 32
Glycidol [CASRN 556-52-5] 34
Hydrocortisone (HC) [CASRN 50-23-7] 37
Indomethacin [CASRN 53-86-1] 40
Isonicotinic Acid Hydrazide (IAH) [CASRN 54-85-3] 45
Lead (II) Acetate Trihydrate [CASRN 6080-56-4] 49
Mannitol [CASRN 69-65-8] 53
Nickel (II) Sulfate Hexahydrate ($NiSO_4$) [CASRN 10101-97-0] 56
Nitrobenzene [CASRN 98-95-3] 60
o-Benzyl-p-chlorophenol (BCP) [CASRN 120-32-1] 62
Perflouorooctanoic Acid (PFOA) [CASRN 335-67-1] 63
Toluene [CASRN 108-88-3] 68
Tributyltin Chloride (TBTC) [CASRN 1461-22-9] 71
Urethane [CASRN 51-79-6] 76
Vanadium Pentoxide [CASRN 1314-62-1] 80
Zinc Dimethyldithiocarbamate (ZDMDC) [CASRN 137-30-4] 84

2,4-Diaminotoluene (DAT) [CASRN 95-80-7]

Human Data

Data from epidemiology studies

No data were located.

In vitro data with cells or cell lines

No data were located.

Mode of action information

No data were located.

Rodent Data

Data from in vivo immunotoxicology or toxicology studies

Female B6C3F1 mice were orally dosed with 25, 50, or 100 mg/kg DAT for 14 days. Absolute and relative (to body weight and to brain weight) liver weights (LOAEL = 100 mg/kg) were increased compared to controls. No effect on absolute or relative spleen weights were reported. However, trend analyses indicated significant changes in relative spleen weight and spleen/brain ratio in treated mice. Leukocyte and lymphocyte numbers also were increased (LOAEL = 100 mg/kg). The percentage of lymphocytes and polymorphonuclear leukocytes also were increased (LOAEL = 50 mg/kg). No changes in serum chemistry parameters (e.g., ALT levels) and bone marrow parameters (e.g., number of cells in the femur) were noted. The number of spleen cells, and percentage of T- and B-cells (LOAELs = 100 and 25 mg/kg, respectively) were altered in treated animals. While the number of spleen cells was decreased 18% at the highest dose tested, the percentage of T-cells and B-cells were increased 75% and 15%, respectively. Peak IgM and IgG responses (in response to sheep erythrocytes) were observed on days 4 and 5 after immunization, respectively. DAT produced a dose-dependent decrease in IgM (46% at 100 mg/kg) and IgG (56% at 100 mg/kg) AFC responses based on total spleen activity. DAT exposure also produced a dose-dependent increase in delayed hypersensitivity response to keyhole limpet hemocyanin (2.2-fold increase at 100 mg/kg). Serum CH50 and C3 levels were not significantly affected in mice treated with DAT. The activity of the reticuloendothelial system was increased in the liver (LOAEL = 100 mg/kg), decreased in the spleen (LOAEL = 50 mg/kg) and kidney (LOAEL = 100 mg/kg), and not affected in the lung or thymus of treated mice. Decreased host resistance (LOAEL = 100 mg/kg) to *Streptococcus pneumoniae* and *Listeria monocytogenes*. However host resistance to B16F10 fibrosarcoma and PYB6 melanoma were not affected (Burns et al. 1994).

In vitro data with cells or cell lines

Spleen cells from female B6C3F1 cells treated with 25, 50, or 100 mg/kg DAT for 14 days were evaluated for response to mitogens and DBA/2 spleen cells. DAT exposure did not affect cell

responses to T-cell mitogens PHA and ConA. An increase in responsiveness to LPS was reported in cells obtained from mice treated with 25 or 50 mg/kg, but not those treated with 100 mg/kg. Spleen cellularity was decreased 20% and 15% at 50 and 100 mg/kg DAT. In response to DBA/2 cells, an enhanced response was observed in responder cells (LOAEL = 100 mg/kg) while no mixed lymphocyte response was noted (Burns et al. 1994).

Peritoneal exudate cells from female B6C3F1 cells treated with 25, 50, or 100 mg/kg DAT for 14 days were allowed to adhere to plastic and the percentage of cells phagocytizing fluorescent Covaspheres or chicken erythrocytes was measured. No significant change in the percentage of phagocytosis was noted at any of the doses (Burns et al. 1994).

Splenic NK cell activity was decreased in cells obtained from mice exposure to DAT for 14 days. A dose-dependent decrease was observed at all effector/target ratios tested (100/1, 50/1, and 25/1). The LOAEL was 50 mg/kg (Burns et al. 1994).

Spleen cell suspensions from female NMRI mice were evaluated to determine whether DAT could modulate luminol-dependent chemiluminescence of phagocytotic cells. Cells were treated with 0.01, 0.1, 1.0, 10, or 100 mg/L DAT. At concentrations greater than 1 mg/L, a dose-dependent decrease in response was observed. When compared to control levels, chemiluminescence was decreased 43%, 90%, and 100% at 1.0, 10, and 100 mg/kg, respectively (Thierfelder and Masihi 1995).

Mode of action information

Based on the combined effects, Burns and colleagues (1994) proposed that DAT affects differentiation and maturation of leukocytes.

References

Burns LA, Bradley SG, White KL, McCay JA, Fuchs BA, Stern M, et al. 1994. Immunotoxicity of 2,4-diaminotoluene in female B6C3F1 mice. Drug and chemical toxicology 17:401–36; doi:10.3109/01480549409017865.

Thierfelder W, Masihi KN. 1995. Effects of trinitrotoluene (TNT) metabolites on chemiluminescence response of phagocytic cells. International journal of immunopharmacology 17: 453–6.

5,5-Diphenylhydantoin (DPH) [CASRN 57-41-0]

Human Data

Data from epidemiology studies

In a study of 51 epileptic patients, 20 of whom had not received anticonvulsant treatment for at least two years and 31 of whom had received DPH at 300 mg/24 hours for at least 4 months, the DPH treated group had decreased serum levels of IgA (156 ± 65 mg/100 mL) and IgM (121 ± 43 mg/100 mL) as compared to untreated epileptics (IgA, 179 ± 70 mg/100 mL; IgM, 133 ± 50 mg/100 mL) or control subjects (n= 15; IgA, 223 ± 49 mg/100 mL; IgM, 163 ± 48 mg/100 mL). Serum IgG levels were not statistically significantly different among the groups. The authors concluded that DPH treatment suppresses the normal function of the humoral immune response and that epilepsy may be a contributing factor (Badawy et al. 1991).

Peripheral blood lymphocytes, serum immunoglobulins and complement (C3 and C4) protein concentrations were determined in 20 patients with idiopathic epilepsy who were receiving 200-300 mg DPH treatment and 30 healthy controls. A significant decrease in T-suppressor cells (28%) and subsequently higher T-helper to T-suppresor lymphocyte ratio (36%) were observed in DPH treated patients. A significant increase in B-lymphocytes (39%) and in serum IgM levels (data in graph) was also observed in DPH treated patients as compared to controls. No significant changes in serum concentrations of IgG, IgA or complement proteins was observed (Basaran et al. 1989).

Peripheral blood lymphocyte subsets, serum immunoglobulins and complement (C3 and C4) protein concentrations were determined in 40 healthy subjects, 30 DPH treated patients (200-300 mg/day), 22 carbamazepine treated patients, and 38 untreated epilepsy patients. Subjects receiving drug therapy had been taking the drug for 3 months up to 20 years. The DPH treated group had decreased IgA (19% and 24%, respectively) and IgG (16% and 14%, respectively) as compared to both healthy subjects and untreated epileptic patients. Significantly lower T-suppressor lymphocyte counts (23% decrease) was observed when compared to healthy controls. Significantly higher T-helper to T-suppressor lymphocyte ratio was observed when compared to healthy subjects and untreated epileptic patients. No significant differences in C3 or C4 protein levels were observed in DPH treated patients as compared to controls (Basaran et al. 1994).

Serum IgA values were determined in 191 patients taking DPH (dosage not provided). A reduction in serum IgA levels was observed in up to 20% of the patients. Cellular immune status was assessed in the 11% of patients with IgA values lower than two standard deviations below the mean and included: lymphocyte counts, lymphocyte population studies and responses to *in vitro* mitogen stimulation. No significant variations from control values were observed in any of the evaluated endpoints (Burks et al. 1989).

In vitro data with cells or cell lines

No data were located.

Mode of action information

DPH (20 µg/mL) induced IL-1 activity and potentiated LPS-induced IL-1 production in human PMBC and in U-937 cells, a stable monocytic cell line (Modeer et al. 1989).

DPH treatment can lead to a decrease of suppressor T cells and a reversible IgA deficiency in patients with epilepsy. Gingival overgrowth, which often develops in patients taking DPH, is hypothesized to be due to increased production of both IL-6 and IL-8, combined with elevations of basic fibroblast growth factor as observed *in vitro* using human gingival fibroblasts (Beghi and Shorvon 2011; Godhwani and Bahna 2016).

Rodent Data

Data from in vivo immunotoxicology or toxicology studies

Male Balb/C mice were given DPH at doses of 0, 25, 50 or 100 mg/kg via oral gavage for 7 days. DPH significantly increased cellularity in the spleen (LOAEL = 25 mg/kg), however, both the direct and indirect plaque-forming cells responses following intraperitoneal injection with sheep erythrocytes, were significantly depressed (LOAELs = 25 mg/kg). A significant decrease in the delayed type hypersensitivity in response to sheep erythrocytes was also observed (LOAEL = 25 mg/kg) (Andrade-Mena et al. 1994).

Pregnant Balb/C mice were treated with DPH at doses of 0, 20, 40, and 60 mg/kg via oral gavage on days 9 through 18 of gestation. A dose-related suppression of humoral immune function (measured as the antibody response to type III pneumococcal polysaccharide) was observed in male and female offspring at 25 days, but not at 15 weeks of age (NOAEL = 20 mg/kg). Female offspring of dams treated with 20 or 60 mg/kg DPH had greater antibody levels than controls. No difference was noted in female offspring of dams treated with 40 mg/kg DPH when compared to controls. Cell-mediated immune function (as measured by delayed-type hypersensitivity response to oxazolone) was not affected in offspring of treated dams. Immunosuppressive effects also were greater in offspring born with an open eye defect, also attributed to DPH treatment (Chapman and Roberts 1984).

Female B10.s, B10.d2 and DBA/2 mice were injected with 2 mg DPH and received a single injection of 10 µg TNP-OVA subcutaneously into the right hind footpad. Popliteal lymph nodes (PLN) were isolated 7 days after injection. DPH increased the number of cells in all three strains (B10.s>B10.d2>DBA/2) (data in graph). IgG1 production to TNP-OVA was increased in all three mouse strains (in B10.d2 about 850-fold; and in B10.s and DBA/2 about 120-fold). DPH treatment did not facilitate immune complex deposition in any of the mouse strains, six days after challenge (Albers et al. 1999).

DPH (administered subcutaneously) produced a significant, dose-dependent response in the PLN assay at 0.5 mg (mean PLN index =1.60 ± 0.18) and 1.0 mg (mean PLN index = 2.79 ± 0.30) as compared to control (mean PLN index = 1.11 ± 0.24) in C3Hf mice. The maximal response occurred at 6-8 days post treatment and returned to normal after 3-4 weeks. The observed response was proposed to be T-lymphocyte dependent since only heterozygous C3H +/nu mice developed PLN enlargement whereas their congenitally athymic C3H nu/nu counterparts did not.

The PLN response to DPH was significantly amplified in thymectomized C57BL/10 mice (PLN index = 6.73 ± 0.83 vs. control PLN index = 2.93 ± 0.53). Proliferation of B lymphocytes was considered a major contributor to the PLN enlargement. A marked increase in IgM and IgG secreting cells was observed following inoculation of BALB/c mice with 1 mg DPH. A maximal increase was observed 10 days after treatment (Gleichmann et al. 1982).

Male C3H/HeN mice were given intraperitoneal injections of DPH (10 mg/mL, once per day) for 28 days and immunized with 100 µg KLH on day 14 and 21. Serum levels of anti-KLH IgG and IgE antibodies were determined on day 28. The KLH-specific IgE response was significantly increased compared to control (data in graph); the IgG response was not changed. Plasma ACTH and corticosterone were significantly higher in DPH-treated mice as compared to controls (data not provided) (Okada et al. 2001).

In vitro data with cells or cell lines

Splenocytes from DPH-treated mice (10 mg/mL for 28 days) immunized with KLH were cultured for 3 days with 50 or 100 µg/mL KLH. No effect on proliferation was noted in splenocytes from DPH-treated mice at either concentration of KLH. Comparatively, splenocytes from control mice immunized with KLH showed a potent proliferative response to stimulation with 50 or 100 µg/mL KLH. T cell function was also impaired in splenocytes from DPH-treated mice, in response to nonspecific mitogens (ConA and LPS) and in response to cross-linking of CD3. The accessory cell function (e.g. macrophages) was also impaired in spleen cells from DPH-treated mice. IL-4 production was significantly enhanced, while IFN-γ and IL-2 production, and NK cell activity were significantly reduced in spleen cells from DPH-treated mice (data in graphs or not provided). IL-1α production was decreased in spleen adherent cells from DPH-treated mice stimulated with *S. aureus*. No effect on IL-6 or IL-12 levels was reported (Okada et al. 2001).

The offspring of female C3H Orleans mice treated with 25 mg/kg diphenylhydantoin 2 times/day throughout gestation, exhibited a reduced thymic cortex and low mitotic activity in the lymphoid population. The reticuloepithelial tissue was enlarged. In the spleen, the white pulp was enlarged due to lymphocyte accumulation. The dams did not exhibit any changes in the thymus or spleen following treatment (Kohler et al. 1987).

Mode of action information

Heat shock proteins were not induced in the PLNs in female BALB/c mice injected subcutaneously with 2 mg DPH (Albers et al. 1996).

Male ICR mice injected intraperitoneally with 60 mg diphenylhydantoin for 3, 8 and 30 days exhibited elevated levels of serum glucocorticoids and thymic atrophy throughout the experiment (Hirai and Ichikawa 1991).

References

Albers R, van der Pijl A, Bol M, Bleumink R, Seinen W, Pieters R. 1999. Distinct immunomodulation by autoimmunogenic xenobiotics in susceptible and resistant mice. Toxicology and applied pharmacology 160:156–62; doi:10.1006/taap.1999.8761.

Albers R, van der Pijl A, Bol M, Seinen W, Pieters R. 1996. Stress proteins (HSP) and chemical-induced autoimmunity. Toxicology and applied pharmacology 140: 70–6.

Andrade-Mena CE, Sardo-Olmedo JA, Ramirez-Lizardo EJ. 1994. Effects of phenytoin administration on murine immune function. Journal of neuroimmunology 50: 3–7.

Badawy AH, Shalaby SA, Abdel Aal SF. 1991. Hydantoin immunosuppression clinical study. Journal of the Egyptian Society of Parasitology 21: 257–62.

Basaran N, Hincal F, Kansu E, Ciger A. 1994. Humoral and cellular immune parameters in untreated and phenytoin-or carbamazepine-treated epileptic patients. International journal of immunopharmacology 16: 1071–7.

Basaran N, Kansu E, Hincal F. 1989. Serum immunoglobulins, complement levels and lymphocyte subpopulations in phenytoin-treated epileptic patients. Immunopharmacology and immunotoxicology 11:335–46; doi:10.3109/08923978909005374.

Beghi E, Shorvon S. 2011. Antiepileptic drugs and the immune system. Epilepsia 52 Suppl 3:40–4; doi:10.1111/j.1528-1167.2011.03035.x.

Burks AW, Charlton R, Casey P, Poindexter A, Steele R. 1989. Immune function in patients treated with phenytoin. Journal of child neurology 4:25–9; doi:10.1177/088307388900400104.

Chapman JR, Roberts DW. 1984. Humoral immune dysfunction as a result of prenatal exposure to diphenylhydantoin: correlation with the occurrence of physical defects. Teratology 30:107–17; doi:10.1002/tera.1420300115.

Gleichmann H, Pals S, Radaszkiewicz T, Wasser M. 1982. T cell-dependent B cell lymphoproliferation and activation induced by the drug diphenylhydantoin. Advances in experimental medicine and biology 149: 617–22.

Godhwani N, Bahna SL. 2016. Antiepilepsy drugs and the immune system. Annals of allergy, asthma & immunology : official publication of the American College of Allergy, Asthma, & Immunology 117:634–640; doi:10.1016/j.anai.2016.09.443.

Hirai M, Ichikawa M. 1991. Changes in serum glucocorticoid levels and thymic atrophy induced by phenytoin administration in mice. Toxicology letters 56: 1–6.

Kohler C, Jeanvoine G, Pierrez J, Olive D, Gerard H. 1987. Modifications of the thymus and splenic thymic dependent zones after in utero exposure to phenytoin: qualitative and quantitative analysis in C3H mice. Developmental pharmacology and therapeutics 10: 405–12.

Modeer T, Karsten J, Weintraub A, Gidlund M, Sundqvist KG. 1989. Phenytoin induces interleukin-1 production in vitro. Life sciences 44: 35–40.

Okada K, Sugiura T, Kuroda E, Tsuji S, Yamashita U. 2001. Phenytoin promotes Th2 type immune response in mice. Clinical and experimental immunology 124: 406–13.

Acetonitrile [CASRN 75-05-8]

Human Data

Data from epidemiology studies

No data were located.

In vitro data with cells or cell lines

No data were located.

Mode of action information

No data were located.

Rodent Data

Data from in vivo immunotoxicology or toxicology studies

In F344/N rats exposed to acetonitrile by inhalation for 13 weeks, gross and histophathologic changes were evaluated in males (800 and 1600 ppm) and females (1600 ppm) that died during the study. Changes reported included thymic atrophy and splenic lymphoid depletion. Decreased absolute and relative thymus weights also were reported in male and female rats (LOAEL = 800 ppm). In F344/N rats exposed to 100, 200, or 400 ppm acetonitrile for 2 years, no immune related effects were reported (National Toxicology Program 1996)

In B6C3F1 mice exposed to acetonitrile by inhalation for 13 weeks, lymphoid depletion and lymphocytolysis in the thymus, spleen and bone marrow was reported in animals that died. A lack of immune effects were reported in mice exposed to acetonitrile for 2 years (NOAEL = 200 ppm) (National Toxicology Program 1996).

Based on a 14-day inhalation study in B6C3F1 mice (doses not provided), acetonitrile was not identified as an immunotoxicant (Luster et al. 1992).

Male Wistar rats were subcutaneously injected with acetonitrile at a dose of 0.8 LD50 (dose not provided). Antibody titer to sheep erythrocytes was decreased by 43%. Additionally, the number of antibody producing cells against sheep erythrocytes and Vi-Ag (no further information provided in article) were decreased by 52% and 27%, respectively. Thymus T-cell count, percentage of natural cytotoxicity (used as a surrogate for NK cell activity), and antibody-dependent cell cytotoxicity also were significantly decreased after acetonitrile exposure. The percentage decreases were calculated as 31%, 52%, and 41%, respectively (Zabrodskii et al. 2002).

In vitro data with cells or cell lines

No data were located.

Mode of action information

No data were located.

References

Luster MI, Portier C, Pait DG, White KL, Gennings C, Munson AE, et al. 1992. Risk assessment in immunotoxicology. I. Sensitivity and predictability of immune tests. Fundam Appl Toxicol 18: 200–210.

National Toxicology Program. 1996. Toxicology and carcinogenesis studies of acetonitrile (CAS NO. 75-05-8) in F44/N rats and B6C3F1 mice (inhalation studies).

Zabrodskii PF, Germanchuk VG, Kirichuk VF, Birbin VS, Chuev AN. 2002. Combined effects of toxicants with various mechanisms of action and mechanical trauma on the immune system. Bulletin of experimental biology and medicine 133: 594–6.

Benzo(a)pyrene [B(a)P] [CASRN 50-32-8]

Human Data

Data from epidemiology studies

No data were located

In vitro data with cells or cell lines

B(a)P (1 µM) and related metabolites significantly increased IgE-mediated histamine release from human basophils, but did not induce cell death. Additionally, a B(a)P metabolite significantly increased IgE-mediated IL-4 production in human basophils (Kepley et al. 2003). In primary human macrophages, 10 µM B(a)P increased expression of TNF-α and IL-1β and produced no effect on IFNγ, IL-6, or IL-12 expression (Lecureur et al. 2005). Comparatively, B(a)P did not modulate IL-6 or IL-8 production in BEAS-2B cells at concentrations ranging from 0.1 to 10 µM (Chowdhury et al. 2017).

B(a)P inhibited anti-CD3 antibody stimulation of human lymphocyte proliferation (IC50 = 12.82 µM) (Carfi et al. 2007).

Six breast epithelial cell strains were incubated with 4µM B(a)P for 24 hours. Gene expression studies (using Hu-Gene 133A arrays) showed that signal log ratio (SLR) was altered by ≥1.5 for 5 immune-related genes in at least one of the tested cell strains. Four genes were upregulated, while one was down regulated. Up regulated genes were IL1B, MAL, HTLF, and SECTM1. CXCL14 gene expression was down-regulated (John et al. 2009).

PBMCs were exposed to ConA and B(a)P and assessed after 3 days. B(a)P dose-dependently decreased DNA synthesis and cell viability in treated cells (LOAELs = 0.01 and 0.1 µM, respectively). The number of cells recovered during the same period also was decreased (LOAEL = 0.01 µM). B(a)P did not affect IL-2 activity or expression of CD25 on small cells or blasts at concentrations up to 1 µM. B(a)P decreased the percentage of blasts that were CD71+ by 13% at 1 µM. Cell cycle analysis indicated that B(a)P increased the percentage of cells in S-phase and decreased the percentage in G0/G1 phase (Mudzinski 1993).

Mode of action information

Calcium mobilization in human T-cells is a proposed mode of action for B(a)P (Krieger et al. 1994). Additionally, Ah receptor activation by B(a)P is proposed to inhibit differentiation of monocytes to macrophages and cell growth of B-cells which may contribute to immunotoxic effects (Allan and Sherr 2005, 2010; van Grevenynghe et al. 2003).

Rodent Data

Data from in vivo immunotoxicology or toxicology studies

Lactating C3H/HeJ dams were dosed with 0.25, 5.0, or 100 pmol/week B(a)P via oral gavage on PND 1, 8, and 15. Pups (5-weeks old) were treated with OVA via intratracheal instillation every

2 weeks for 6 weeks. B(a)P had no effect on the number of macrophages or lymphocytes in BAL from male or female offspring not treated with OVA. Additionally, no effect was noted on the number of macrophages or lymphocytes in B(a)P-treated offspring that were immunized with OVA (when compared with offspring only treated with OVA). IL-4, IL-5, IL-13, IL-33, and IFN-γ levels in the BAL were not affected in offspring not treated with OVA. Increased IL-33 and IFN-γ levels were observed in OVA-sensitized female offspring lactationally exposed to 5.0 and 100 pmol/week B(a)P, respectively. Lactational exposure to 0.25 B(a)P increased the total number of mediastinal lymph node cells in males. Lactational 100 pmol/week B(a)P increased numbers of TCRβ+ and CD86+ cells compared with vehicle in non-sensitized male offspring. In non-sensitized female offspring, lactational exposure to 100 pmol/week B(a)P increased numbers of CD11c+ PDCA-1-, CD28+, TCRβ+CD28+, MHC Class II+, and MHC Class II+CD86+ cells. In OVA-sensitized female offspring, a significant increase in CD11c+PDCA-1+ and CD11c+PDCA-1- cells was observed after exposure to 0.25 and 5.0 pmol/week, respectively (Yanagisawa et al. 2018).

Pregnant C3H/HeB mice were administered 150 mg/kg B(a)P via intraperitoneal injection on GD 11; immune effects were assessed at parturition and again one week after parturition. A significant reduction in newborn CD4+CD8+ (46%), CD4+CD8+Vγ2+ (60%), and CD4+CD8+Vβ2+ (53%) thymocytes were noted. Additionally, CD4+ splenocytes from 1-week- old offspring were significantly reduced (50%) (Rodriguez et al. 1999).

B6C3F1 mice were administered 0.4, 4.0, or 40 mg/kg B(a)P by intratracheal instillation for seven days and immunized with sheep erythrocytes after the last B(a)P exposure. Decreased formation of antigen-specific AFC (by 60%) was observed at 40 mg/kg B(a)P in LALN. When sheep erythrocytes were administered by intraperitoneal injection, an increase in antigen-specific AFC was observed at 40 mg/kg B(a)P in LALN. However, the levels of AFC in the spleen were decreased (Schnizlein et al. 1987).

B6C3F1 mice (3-6 months, 13-16 months, and 23-26 months) were administered 40 mg/kg B(a)P for 8 days by intraperitoneal injection. Mice also were immunized with sheep erythrocytes after day 4 of the B(a)P treatment. Spleens were removed and splenocytes assessed for formation of AFCs. Decreased formation of AFCs was noted in splenocytes from all three age groups. In two sets of experiments, the observed decreases were 23%-43% in mice ages 3-6 months, 63%- 84% in mice ages 16-18 months, and 93% in mice ages 23-26 months (Lyte and Bick 1985).

B6C3F1 mice were administered 5, 20, or 40 mg/kg B(a)P for 14 days by subcutaneous injection. Spleens were removed and ConA-induced production of IL-2 and IL-3 were assessed. While splenocyte IL-2 production was decreased in a dose dependent manner, no effect on splenocyte IL-3 production was noted. As shown in other studies, B(a)P decreased responses to sheep erythrocytes (>95% inhibition). Addition of exogenous IL-2 to the treated splenocytes, reversed the B(a)P-induced inhibition of responses to sheep erythrocytes (Lyte et al. 1987; Lyte and Bick 1986).

Female B6C3F1 mice were administered 10 subcutaneous injections of B(a)P over a 14-day period at doses of 5, 20, or 40 μg/g. KLH-sensitization did not affect delayed hypersensitivity

responses at the tested doses. Additionally, B(a)P treatment did not induce rejection to DBA mice skin that was grafted onto mice. Proliferative responses to PHA were dose-dependently decreased (LOAEL = 20 µg/g B(a)P). Spontaneous and LPS-induced proliferative responses were increased at 5 µg/g B(a)P and significantly decreased at 40 µg/g B(a)P. MLC responses, and the percentage of spleen cells with T- and B-cell surface markers were not significantly affected at any of the tested doses. Additionally, NK cell activity against YAC- I target cells was not impacted in mice treated with 40 µg/g B(a)P (data not provided). Serum IgG levels were dose-dependently decreased in treated mice (18-24%). A reduction in the number of antibody plaque forming cells to sheep erythrocytes and LPS were noted (LOAELs = 20 and 5 µg/g B(a)P, respectively). B(a)P exposure decreased response to TNP-Ficoll without effects on TNP-LPS response. Host resistance studies showed that B(a)P had no effect on PYB6 tumor incidence or susceptibility to *L. monocytogenes* (Dean et al. 1983).

In vitro data with cells or cell lines

Rat and mouse spleen cells were treated with B(a)P for 24 hours and then LPS or PHA for 48 hours. Then, mitogen stimulation was assessed by 5-bromo-2'-deoxyuridine incorporation. B(a)P inhibited cellular proliferation for both species at similar concentrations (data in graphs). B(a)P also inhibited rat spleen proliferation that was stimulated by ConA (data provided in graph). B(a)P inhibited anti-CD3 antibody stimulation of mouse lymphocyte proliferation (IC50 > 160 µM) (Carfi et al. 2007).

B(a)P decreased viability of mouse antigen presenting cells (APC) and increased expression of CD86 expression on APC (LOAELs = 0.1 µM). In murine splenocytes, B(a)P decreased cell viability and proliferation (LOAELs = 0.1 and 1.0 µM, respectively). B(a)P did not modulate the expression of T-cell receptors or CD19 at any of the tested concentrations in murine splenocytes (Chowdhury et al. 2017).

B(a)P decreased ConA induced cellular proliferation of mouse splenic T-cells in a dose-dependent manner (LOAEL = 0.1 µg/mL). Inhibition of IL-2, IL-4, and IFN-γ also was observed in ConA-stimulated splenic T-cells (LOAELs = 0.1, 0.2, and 0.1 µg/mL, respectively) (Guan et al. 2017).

B(a)P inhibited spleen cell response to sheep erythrocytes in a concentration dependent manner (LOAEL = 0.01 µM). B(a)P also inhibited one-way mixed lymphocyte response with a maximal inhibition of 19% (Urso et al. 1986). Similar response of murine spleen cell response to sheep erythrocytes was reported by Kawabata and White (1987) (LOAEL = 1 nM) after incubation for 5 days.

Splenocytes from B6C3F1 mice (3-6 months and 23-26 months) were exposed to 1, 10, or 50 µg/mL B(a)P and sheep erythrocytes for 4-5 days. After end of exposure period, the number of AFCs was determined. Dose-dependent decrease in the number of cells was observed in splenocytes from both age groups (data in graphs) (Lyte and Bick 1985).

B(a)P (in PVP-NaCl) dose-dependently increased LPS-induced IL-1 production by peritoneal exudate macrophages isolated from B6C3F1 mice; tested concentrations ranged from 25 to 800

μg/mL. A concurrent decrease in cell viabilities was noted at the same test concentrations. Comparatively, when B(a)P was dissolved in corn oil no effect on IL-1 production or cell viabilities was noted (Lyte and Bick 1986).

Mode of action information

Disruption of T-cell activities has been associated with B(a)P induced immunotoxic effects (Urso et al. 1986). Modulation of mouse splenic T-cell effects was associated with modulation of calcium levels; which was associated with suppression of the NF-□B and NFAT pathways (Guan et al. 2017).

In addition to T-cell effects, modulation of B-cell population or responses, or macrophage functions also have been implicated in B(a)P mode of action (Saxena et al. 2018; Urso et al. 1986). Hardin and colleagues (1992) proposed that B(a)P-induced suppression of B-cell lymphopoiesis was, partially, produced through induction of programmed cell death. Ah-receptor dependent- and/or independent-pathways could produce the observed effects.

References

Allan LL, Sherr DH. 2005. Constitutive activation and environmental chemical induction of the aryl hydrocarbon receptor/transcription factor in activated human B lymphocytes. Molecular pharmacology 67:1740–50; doi:10.1124/mol.104.009100.

Allan LL, Sherr DH. 2010. Disruption of human plasma cell differentiation by an environmental polycyclic aromatic hydrocarbon: a mechanistic immunotoxicological study. Environmental health : a global access science source 9:15; doi:10.1186/1476-069x-9-15.

Carfi M, Gennari A, Malerba I, Corsini E, Pallardy M, Pieters R, et al. 2007. In vitro tests to evaluate immunotoxicity: a preliminary study. Toxicology 229:11–22; doi:10.1016/j.tox.2006.09.003.

Chowdhury PH, Kitamura G, Honda A, Sawahara T, Hayashi T, Fukushima W, et al. 2017. Synergistic effect of carbon nuclei and polyaromatic hydrocarbons on respiratory and immune responses. Environmental toxicology 32:2172–2181; doi:10.1002/tox.22430.

Dean JH, Luster MI, Boorman GA, Lauer LD, Leubke RW, Lawson L. 1983. Selective immunosuppression resulting from exposure to the carcinogenic congener of benzopyrene in B6C3F1 mice. Clinical and experimental immunology 52: 199–206.

Guan S, Huang Y, Feng Z, Xu L, Jin Y, Lu J. 2017. The toxic effects of benzo[a]pyrene on activated mouse T cells in vitro. Immunopharmacology and immunotoxicology 39:117–123; doi:10.1080/08923973.2017.1299173.

Hardin JA, Hinoshita F, Sherr DH. 1992. Mechanisms by which benzo[a]pyrene, an environmental carcinogen, suppresses B cell lymphopoiesis. Toxicology and applied pharmacology 117: 155–64.

John K, Keshava C, Richardson DL, Weston A, Nath J. 2009. Immune response signatures of benzo{alpha}pyrene exposure in normal human mammary epithelial cells in the absence or presence of chlorophyllin. Cancer genomics & proteomics 6: 1–11.

Kawabata TT, White KL Jr. 1987. Suppression of the vitro humoral immune response of mouse splenocytes by benzo(a)pyrene metabolites and inhibition of benzo(a)pyrene-induced immunosuppression by alpha-naphthoflavone. Cancer research 47: 2317–22.

Kepley CL, Lauer FT, Oliver JM, Burchiel SW. 2003. Environmental polycyclic aromatic hydrocarbons, benzo(a) pyrene (BaP) and BaP-quinones, enhance IgE-mediated histamine release and IL-4 production in human basophils. Clinical immunology (Orlando, Fla) 107: 10–9.

Krieger JA, Born JL, Burchiel SW. 1994. Persistence of calcium elevation in the HPB-ALL human T cell line correlates with immunosuppressive properties of polycyclic aromatic hydrocarbons. Toxicology and applied pharmacology 127:268–74; doi:10.1006/taap.1994.1161.

Lecureur V, Ferrec EL, N'Diaye M, Vee ML, Gardyn C, Gilot D, et al. 2005. ERK-dependent induction of TNFalpha expression by the environmental contaminant benzo(a)pyrene in primary human macrophages. FEBS letters 579:1904–10; doi:10.1016/j.febslet.2005.01.081.

Lyte M, Bick PH. 1985. Differential immunotoxic effects of the environmental chemical benzo[a]pyrene in young and aged mice. Mechanisms of ageing and development 30: 333–41.

Lyte M, Bick PH. 1986. Modulation of interleukin-1 production by macrophages following benzo(a)pyrene exposure. International journal of immunopharmacology 8: 377–81.

Lyte M, Blanton RH, Myers MJ, Bick PH. 1987. Effect of in vivo administration of the carcinogen benzo(a)pyrene on interleukin-2 and interleukin-3 production. International journal of immunopharmacology 9: 307–12.

Mudzinski SP. 1993. Effects of benzo[a]pyrene on concanavalin A-stimulated human peripheral blood mononuclear cells in vitro: inhibition of proliferation but no effect on parameters related to the G1 phase of the cell cycle. Toxicology and applied pharmacology 119:166–74; doi:10.1006/taap.1993.1057.

Rodriguez JW, Kirlin WG, Wirsiy YG, Matheravidathu S, Hodge TW, Urso P. 1999. Maternal exposure to benzo[a]pyrene alters development of T lymphocytes in offspring. Immunopharmacology and immunotoxicology 21:379–96; doi:10.3109/08923979909052769.

Saxena N, Kaur AP, Chandra NC. 2018. Differential Response of B Cells to an Immunogen, a Mitogen and a Chemical Carcinogen in a Mouse Model System. Asian Pacific journal of cancer prevention : APJCP 19:81–90; doi:10.22034/apjcp.2018.19.1.81.

Schnizlein CT, Munson AE, Rhoades RA. 1987. Immunomodulation of local and systemic immunity after subchronic pulmonary exposure of mice to benzo(a)pyrene. International journal of immunopharmacology 9: 99–106.

Urso P, Gengozian N, Rossi RM, Johnson RA. 1986. Suppression of humoral and cell-mediated immune responses in vitro by benzo(a)pyrene. Journal of immunopharmacology 8: 223–41.

van Grevenynghe J, Rion S, Le Ferrec E, Le Vee M, Amiot L, Fauchet R, et al. 2003. Polycyclic aromatic hydrocarbons inhibit differentiation of human monocytes into macrophages. Journal of immunology (Baltimore, Md : 1950) 170: 2374–81.

Yanagisawa R, Koike E, Win-Shwe TT, Ichinose T, Takano H. 2018. Effects of lactational exposure to low-dose BaP on allergic and non-allergic immune responses in mice offspring. Journal of immunotoxicology 15:31–40; doi:10.1080/1547691x.2018.1442379.

Cadmium Chloride [CASRN 10108-64-2]

Human Data

Data from epidemiology studies

No data were located.

In vitro data with cells or cell lines

Cadmium chloride (10-100 µM) inhibited NK (against K562 cells) and antibody-dependent cellular (against P815 cells) cytotoxicity (ADCC) in peripheral blood lymphocytes in a concentration-dependent manner. The estimated 50% inhibition doses (ID50) for NK and ADCC activities were 50 and 100 µM, respectively. NK and ADCC activities were not significantly affected by changing the effector cell:target cell ratios. Cadmium chloride also inhibited cytotoxic activity against K562 or Daudi cells in activated IL-2 cells (data in graph). Time- course studies showed that a significant decrease in NK and ADCC activities was observed when added at 90 minutes after the start of the experiment (Cifone et al. 1990).

Viability of A549 cells was decreased (44.5% of control) after exposure to 75 µM cadmium chloride. At the same concentration, cadmium chloride increased select cytokine levels (e.g., IFN-γ, IL-3, IL-5, IL-10, IL-15, and IL-16). Comparatively, cadmium chloride decreased TGF-β3 levels (Odewumi et al. 2016).

Mode of action information

In vitro studies suggest that in peripheral blood lymphocytes, cadmium chloride modulated phosphoinositide hydrolysis induced by a target molecule. This modulation is proposed to lead to inhibited NK activity (Cifone et al. 1990).

Proposed direct action of cadmium on immunocompetent cells stimulates production and release of cytokines, which may produce proinflammatory effects (Marth et al. 2000).

Rodent Data

Data from in vivo immunotoxicology or toxicology studies

Female BDF1 mice were provided drinking water containing 5, 10, or 50 µg/mL cadmium (as cadmium chloride) for 3 weeks. Antibody response to sheep erythrocytes was decreased in a dose-dependent manner. Splenic plaque-forming cell number decreased by 16% to 28%. A dose-dependent increase in LPS-induced proliferation also was observed (LOAEL = 10 µg/mL). In the absence of a mitogen, cadmium chloride also increased lymphocyte proliferation (LOAEL = 10 µg/mL). No effect was observed when ConA mitogen was used to stimulate proliferation (Blakley 1985).

Female CD1 mice were provided drinking water containing 5, 10, or 50 µg/mL cadmium (as cadmium chloride) for 3 weeks. *In vivo* T-lymphocyte independent (against DNP-Ficoll) and T-

lymphocyte and macrophage independent (against *E. coli*) responses were increased by cadmium exposure (Blakley and Tomar 1986).

Female BDF1 mice were provided drinking water containing 50 µg/mL cadmium (as cadmium chloride) for 3 weeks. Spleen cell suspensions from pooled spleens were separated by adherence techniques and antibody production against sheep erythrocytes was assessed. Suppressed antibody production (26%-34%) was noted in cultures that contained cadmium-exposed T-lymphocytes. Antibody production was similar to controls in cultures that contained cadmium-exposed macrophages (Blakley and Tomar 1986).

Male Sprague-Dawley rats were administered 0.7 or 6 mg/kg cadmium (as cadmium chloride) by oral gavage for 28 days. Splenocyte proliferation was significantly decreased (76% of control) in rats that were administered 6 mg/kg cadmium. Splenocyte IL-2 production also was increased after administration of 6 mg/kg cadmium, when production was normalized with cell number. No effect was noted on splenocyte IFN-γ production (Wang et al. 2017).

Immunotoxic effects in offspring were noted after exposure to cadmium chloride *in utero* or through milk. In ICR mice administered 2.5 or 5.0 mg/kg cadmium chloride on GD 16, a significant increase in offspring spleen weight was reported (LOAEL = 2.5 mg/kg). Unstimulated spleen lymphocyte proliferation was significantly increased at both tested doses (1.5- to 2-fold). Additionally, ConA, PHA, and LPS stimulation was increased in treated animals (LOAELs = 5.0, 5.0, and 2.5 mg/kg). No effect on delayed-type hypersensitivity to sheep erythrocytes was reported, but an increase in total Ig and IgM antibody titer was noted at 2.5 mg/kg (Soukupova et al. 1991). In offspring that were exposed to cadmium chloride through maternal milk (dams received 5 ppm or 10 ppb cadmium chloride in water until weaning) decreased spleen weights were observed in females, but not males (data in graphs). The effect was greater in lower dosed females. Effects on organ weight did not persist to adulthood. In adult and juvenile rats, effects on cytotoxic activity of splenic NK-cells was noted (data in graphs). Additionally, cadmium chloride inhibited ConA-induced thymocyte proliferation in both male and female adult rats (Pillet et al. 2005).

Female C57BL/6 mice were exposed (nose-only) to aerosolized cadmium chloride (60-minute exposure to 0.88 mg Cd/m^3) and examined 5-18 days later. Decreased splenic cell viability was observed (data in graph). Significant decreases of proliferative responses to LPS and PHA, and inhibition of IgM secretion in response to sheep erythrocytes were observed. Comparatively, oral chronic exposure (5, 100, or 300 ppm cadmium chloride in water for 12-16 weeks) suppressed IgM response to sheep erythrocytes, without effects on cell viability (Krzystyniak et al. 1987).

In vitro data with cells or cell lines

Splenocytes isolated from male Sprague-Dawley rats were treated with ConA for 24 hours, followed by incubation with 5, 10, or 20 µM cadmium chloride for 4 or 24 hours. After exposure for 4 hours, decreased IL-2 (LOAEL = 5 µM) and IFN-γ (LOAEL = 10 µM) production was observed in the absence of effects on cell proliferation. After exposure for 24 hours, decreased IL-2 (LOAEL = 5 µM) production and cell proliferation (LOAEL = 10 µM) were observed. When cytokine production after 24-hour exposure was normalized based on cell number,

increased IFN-γ production (LOAEL = 10 μM) was noted. For IL-2 production, a significant decrease was noted at 5 μM and an increase was noted at 20 μM (Wang et al. 2017).

Mode of action information

In RAW264.7 cells, cadmium chloride upregulation of COX-2 and MIP-2 was associated with activation of the phosphatidylinositol 3-kinase/Akt signaling pathway (Huang et al. 2014).

Cadmium chloride also may induce overstimulation of nuclear factors of activated T-cells to activate Jurkat T cells (Colombo et al. 2004).

References

Blakley BR. 1985. The effect of cadmium chloride on the immune response in mice. Canadian journal of comparative medicine : Revue canadienne de medecine comparee 49: 104–8.
Blakley BR, Tomar RS. 1986. The effect of cadmium on antibody responses to antigens with different cellular requirements. International journal of immunopharmacology 8: 1009–15.
Cifone MG, Procopio A, Napolitano T, Alesse E, Santoni G, Santoni A. 1990. Cadmium inhibits spontaneous (NK), antibody-mediated (ADCC) and IL-2-stimulated cytotoxic functions of natural killer cells. Immunopharmacology 20: 73–80.
Colombo M, Hamelin C, Kouassi E, Fournier M, Bernier J. 2004. Differential effects of mercury, lead, and cadmium on IL-2 production by Jurkat T cells. Clinical immunology (Orlando, Fla) 111:311–22; doi:10.1016/j.clim.2004.02.005.
Huang YY, Xia MZ, Wang H, Liu XJ, Hu YF, Chen YH, et al. 2014. Cadmium selectively induces MIP-2 and COX-2 through PTEN-mediated Akt activation in RAW264.7 cells. Toxicological sciences : an official journal of the Society of Toxicology 138:310–21; doi:10.1093/toxsci/kfu013.
Krzystyniak K, Fournier M, Trottier B, Nadeau D, Chevalier G. 1987. Immunosuppression in mice after inhalation of cadmium aerosol. Toxicology letters 38: 1–12.
Marth E, Barth S, Jelovcan S. 2000. Influence of cadmium on the immune system. Description of stimulating reactions. Central European journal of public health 8: 40–4.
Odewumi CO, Latinwo LM, Ruden ML, Badisa VL, Fils-Aime S, Badisa RB. 2016. Modulation of cytokines and chemokines expression by NAC in cadmium chloride treated human lung cells. Environmental toxicology 31:1612–1619; doi:10.1002/tox.22165.
Pillet S, Rooney AA, Bouquegneau JM, Cyr DG, Fournier M. 2005. Sex-specific effects of neonatal exposures to low levels of cadmium through maternal milk on development and immune functions of juvenile and adult rats. Toxicology 209:289–301; doi:10.1016/j.tox.2004.12.007.

Soukupova D, Dostal M, Piza J. 1991. Developmental toxicity of cadmium in mice. II. Immunotoxic effects. Functional and developmental morphology 1: 31–6.
Wang P, Wang J, Sun YJ, Yang L, Wu YJ. 2017. Cadmium and chlorpyrifos inhibit cellular immune response in spleen of rats. Environmental toxicology 32:1927–1936; doi:10.1002/tox.22415.

Dibromoacetic Acid (DBAA) [CASRN 631-61-1]

Human Data

Data from epidemiology studies

No studies were located.

In vitro data with cells or cell lines

In cultured PBMCs collected from healthy, non-smoking volunteers and cultured in DBAA for four hours, DBAA increased the percentage of necrotic human PBMC and decreased PBMC cell size (LOAEL = 5 mM). Increases in the percentage of apoptotic cells and PBMC granulation also was reported (LOAEL = 1 and 5mM, respectively). Caspase-8, -9, and -3 expression were upregulated at 1 and 5 mM. Increased transmembrane mitochondrial potential and levels of reactive oxygen species (ROS) also were noted with DBAA exposure (LOAEL = 1 and 0.1 mM) (Michalowicz et al. 2015).

Mode of action information

DBAA may increase ROS levels and transmembrane mitochondrial potentials (Michalowicz et al. 2015).

Rodent Data

Data from in vivo immunotoxicology or toxicology studies

In female F344/N rats exposed to DBAA for 3 months (0-2000 mg/L in drinking water), minimal to mild hematopoietic cell proliferation was noted at the highest dose. A similar effect was not observed in males. While no spleen effects were noted in B6C3F1 mice exposed to DBAA for 3 months (0-2000 mg/L in drinking water), thymus atrophy was reported in males and females (LOAEL = 1000 and 2000 mg/L, respectively) (National Toxicology Program 2007).

In male and female BALB/c mice orally gavaged with 5, 20, or 50 mg/kg DBAA for 28 days altered spleen and thymus weights, and splenic and thymic cellularity were reported. DBAA also inhibited B-cell proliferation (LOAEL = 20 mg/kg). DBAA increased T-cell mitogenesis (value not provided) at 20 mg/kg. DBAA increased apoptosis in spleen and thymus in a dose-dependent manner (values not provided). Additionally, DBAA exposure altered the expression of apoptosis-related genes in spleens and thymus of treated mice. In the thymus, expression of Fas and TRAF2 were altered (2-2.5 fold). In spleens of treated mice, expression of Fas and TRAF2 were increased 5-fold while bcl-2 expression was decreased 1.5-fold. Increased protein expression of Fas and FasL also were observed in spleen and thymus of treated mice (LOAEL = 5 mg/kg) (Gao et al. 2008).

Table 1. Data from Gao et al. (2008)

Endpoint	0 mg/kgs	5 mg/kg	20 mg/kg	50 mg/kg
**Male**				
Spleen weight (mg)	80.5 ± 2.7	88.7 ± 3.7	91.2 ± 3.4**	94.0 ± 2.5***
Thymus weight (mg)	43.1 ± 3.4	37.6 ± 2.0	33.3 ± 2.8**	33.4 ± 2.3**
Relative spleen weight (mg/g)	3.68 ± 0.14	3.87 ± 0.22	4.18 ± 0.20*	4.23 ± 0.09*
Relative thymus weight (mg/g)	1.85 ± 0.14	1.65 ± 0.09	1.50 ± 0.13**	1.50 ± 0.11**
Splenic cellularity (x10^7)	9.00 ± 0.44	9.09 ± 0.28	7.63 ± 0.65	6.11 ± 0.38***
Thymic cellularity (x10^7)	8.88 ± 1.06	9.16 ± 0.28	7.39 ± 0.47	5.37 ± 0.82**
**Female**				
Spleen weight (mg)	78.9 ± 2.2	100.1 ± 7.7**	102.4 ± 5.0**	101.2 ± 4.8**
Thymus weight (mg)	46.9 ± 3.7	47.3 ± 3.9	35.8 ± 2.3*	29.5 ± 3.3***
Relative spleen weight (mg/g)	3.99 ± 0.18	5.33 ± 0.45**	5.29 ± 0.27**	5.22 ± 0.19**
Relative thymus weight (mg/g)	2.39 ± 0.17	2.41 ± 0.18	1.87 ± 0.11*	1.55 ± 0.17***
Splenic cellularity (x10^7)	8.60 ± 0.55	8.28 ± 1.19	6.14 ± 1.27	4.65 ± 0.43**
Thymic cellularity (x10^7)	7.97 ± 0.53	7.08 ± 0.74	5.42 ± 0.79*	4.28 ± 0.39***

Data are presented as mean ± SEM.
***p < 0.001, **p < 0.01, *p < 0.05, significance assessed by ANOVA when compared with control group (DBA 0 mg/kg).

Increased neuronal expression of immune factors was noted in Sprague-Dawley rats administered 20, 50, or 125 mg/kg DBAA via intragastric injection for 4-weeks. mRNA expression of Iba-1, NK-□B, IL-6, IL-1β, and TNF-α were increased in the pre-frontal cortex and hippocampus of treated rats (LOAEL = 50 mg/kg for all brain regions). Protein levels of Iba-1, NK-□B, IL-6, IL-1β, and TNF-α also were significantly increased in the same brain regions.
Protein expression LOAEL in the pre-frontal cortex for Iba-1, NK-□B, IL-6, IL-1β, and TNF-α was 50 mg/kg. Protein expression LOAEL In the hippocampus was 100 mg/kg for NK-□B and 50 mg/kg for other evaluated cytokines (Jiang et al. 2017).

Female B6C3F1 mice were given drinking water with 125, 500, or 1000 mg/L for 28 days. A significant decrease in thymus weight was noted at 500 and 1000 mg/L (19%). No effect on absolute or relative spleen weight, or relative thymus weight was reported. A non-dose response decrease (19%) in total spleen cell number and number of CD+CD- T-lymphocytes (13%) was observed at 500 and 125 mg/L, respectively. A significant decrease in absolute (38%) and percent (22%) NK1.1+CD3- cells was noted at 500 mg/L. Significant decreases in absolute and percent splenic macrophages also were observed (LOAEL = 500 and 1000 mg/L, respectively). No effects on absolute or percent Ig+, CD3+, CD4-CD+, or CD4+CD8+ markers were noted. No effects on AFC response or IgM antibody titers in response to exposure to sheep red blood cells were noted. Additionally, no impact on response to allogeneic spleen cell stimulation was noted.

A significant decrease in cytotoxicity was only observed after splenocyte NK cell activity was augmented with poly-IC; the effect was only observed at 125 mg/L. Host resistance to *Streptococcus pneumoniae*, *Plasmodoium yoelii*, and B16F10 melanoma tumors was not affect by treatment (Smith et al. 2010).

In vitro data with cells or cell lines

DBAA decreased thymocyte (obtained from BALB/c mice) proliferation at exposure lengths of at least 6 hours. At 6 hours, a significant decrease in proliferation was only observed at 40 µM. Comparatively, at 12, 24, and 48 hour exposure periods a significant decrease in proliferation was observed at 5, 10, 20 and 40 µM. DBAA also decreased IL-2 and IL-4 secretion (LOAEL = 10 and 5 µM, respectively). DBAA also increased late and early apoptosis (LOAEL = 5 and 10 µM), without effects on the percentage of necrotic cells. DBAA induced an increase in the percentage of cells in the G0/G1 phase and decreased the percentage of cells in the S phase. Increased intracellular thymocyte calcium levels (LOAEL = 5µM) and thymocyte Fas and FasL protein levels were reported (LOAELs = 10 µM for both proteins). Additionally, bcl-2 protein level was significantly decreased at all tested concentrations (LOAEL = 5 µM) (Gao et al. 2016).

Peritoneal exudate cells, obtained from B6C3F1 mice treated with 125, 500, or 1000 mg/L DBAA for 28 days, were evaluated for their ability to suppress B16F10 melanoma tumor cell proliferation *in vitro.* Treatment did not affect the ability of macrophages obtained from treated animals to suppress proliferation (Smith et al. 2010).

In Cl.Ly1 + 2/-9 cells, non-adherent cloned T-cell line derived from spleen cells from C57BL/6TL+ mice, DBAA (1-40 µM) decreased cell viability after exposure for 24, 48, or 72 hours (LOAEL = 1 µM). An increase in the mean percentage of early, late and total apoptotic cells also was noted (LOAEL = 5 µM) (Zhou et al. 2018).

Mode of action information

Overall, studies suggest that DBAA produces immunotoxic effects through modulation of T-cell mediated cell immunity. T-cell apoptosis, through extrinsic and intrinsic pathways, are proposed to play a role in the mode of action. Apoptosis may occur through a variety of pathways including modulation of transmembrane potential, the Fas/FasL pathway, modulation of intracellular calcium, and cell cycle arrest (Gao et al. 2008; Gao et al. 2016).

References

Gao S, Wang Y, Zhang P, Dong Y, Li B. 2008. Subacute oral exposure to dibromoacetic acid induced immunotoxicity and apoptosis in the spleen and thymus of the mice. Toxicological sciences : an official journal of the Society of Toxicology 105:331–41; doi:10.1093/toxsci/kfn139.

Gao SY, Zhou XR, Gong TT, Jia LM, Li BX. 2016. Dibromoacetic Acid Induces Thymocyte Apoptosis by Blocking Cell Cycle Progression, Increasing Intracellular Calcium, and the Fas/FasL Pathway in Vitro. Toxicologic pathology 44:88–97; doi:10.1177/0192623315612939.

Jiang W, Li B, Chen Y, Gao S. 2017. The toxic influence of dibromoacetic acid on the hippocampus and pre-frontal cortex of rat: involvement of neuroinflammation response and oxidative stress. Metabolic brain disease 32:2009–2019; doi:10.1007/s11011-017-0095-0.

Michalowicz J, Wroblewski W, Mokra K, Macczak A, Kwiatkowska M. 2015. Comparative study of the effect of chloro-, dichloro-, bromo-, and dibromoacetic acid on necrotic, apoptotic and morphological changes in human peripheral blood mononuclear cells (in vitro study).

Toxicology in vitro : an international journal published in association with BIBRA 29:1416–24; doi:10.1016/j.tiv.2015.05.021.

National Toxicology Program. 2007. Toxicology and carcinogenesis studies of dibromoacetic acid (Cas No. 631-64-1) in F344/N rats and B6C3F1 mice (drinking water studies). National Toxicology Program technical report series 1–320.

Smith MJ, Germolec DR, Luebke RW, Sheth CM, Auttachoat W, Guo TL, et al. 2010. Immunotoxicity of dibromoacetic acid administered via drinking water to female B(6)C(3)F(1) mice. Journal of immunotoxicology 7:333–43; doi:10.3109/1547691x.2010.519744.

Zhou XR, Jiang WB, Zhang YT, Gong TT, Gao SY. 2018. Dibromoacetic acid induced Cl.Ly1+2/-9 T-cell apoptosis and activation of MAPKs signaling cascades. Toxicology in vitro : an international journal published in association with BIBRA 47:156–164; doi:10.1016/j.tiv.2017.11.006.

Dibutyl phthalate (DBP) [CASRN 84-74-2]

Human Data

Data from epidemiology studies

No data were located.

In vitro data with cells or cell lines

DBP significantly decreased phagocytotic capacity of differentiated THP-1 cells at all tested concentrations (LOAEL = 0.001µM). DBP also increased TNF-α secretion (LOAEL = 0.1 µM). Comparatively, DBP had no effect on IL-1β or IL-8 secretion from differentiated THP-1 cells (NOAEL = 0.001 µM) (Couleau et al. 2015).

High-density microarray studies were conducted using normal human mammary epithelial cell strains obtained from discarded tissues; cells were treated with 1 µM DBP for 10 hours. Gene expression of 29 genes were increased in all four isolated cell strains. Gene expression of 28 genes were decreased in all four isolated cell strains including genes involved in the immune response (TNF-α-induced protein 3; values not provided) (Gwinn et al. 2007).

DBP (tested at 0.1 and 100 µM) increased IL-6, CXCL8, and IL-10 secretion from monocytes/macrophages, isolated from blood of healthy individuals. The cells were, stimulated with *E. coli* lipopolysaccharide (LPS) for 1 hour. Comparatively, DBP did not affect IL-1β and decreased TNF-α secretion from the cells. For all affected cytokines the LOAEL was 100 µM. For phytohemagglutinin-P (PHA-P) stimulated T cells, DBP decreased IL-2, IL-4, TNF-α and IFN-γ secretion (LOAEL for all cytokines = 100 µM). No effect on IL-6 or IL-10 secretion was observed in the PHA-P stimulated T cells treated with DBP. Metabolism studies indicated that DBP was metabolized to monobutyl phthalate *in vitro*. Additionally, secretion patterns of monobutyl phthalate was similar to those observed for DBP (Hansen et al. 2015).

DBP increased IL-1β gene expression (as assessed by RT-PCR) in human corneal endothelial cell line B4G12 at all tested concentrations (LOAEL = 1 µM). IL-8 gene expression was increased at 1 and 10 µM (values not provided). IL-β, IL-8, and IL-6 secretion from cells also was increased. IL-6 and IL-8 LOAEL values were 10 and 5 µM, respectively. Significant IL-1β secretion was only observed at 1 µM. [Note: The authors note that secretion for IL-1β and IL-6 was low and quantification was approximate] (Kruger et al. 2012).

In THP-1 cells, DBP did not induce release of IL-18 (doses tested not provided) or IL-8 (NOAEL = 250 µM), or expression of CD86 (NOAEL = 250 µM). However, DBP did induce IL-8 mRNA expression at 500 µM after exposure for 3 hours (values not provided in paper) (Lourenco et al. 2015).

In HepG2 and L02 (normal human liver) cell lines, DBP (10 µM and 25 µM, respectively) significantly increased levels of mature caspase-1, IL-1β, and nucleotide oligomerization domain (NOD) like receptor family, pyrin domain containing 3 (NLRP3) (values not provided). KN-62,

a P2X7 receptor inhibitor, attenuated DBP-induced effects on caspase-1, IL-1β, and NLRP3 (Ni et al. 2016).

In primary human keratinocytes cultured on an amorphous pseudodermis, DBP increased TSLP (thymic stromal lymphopoietin) mRNA expression (Schuepbach-Mallepell et al. 2013).

Mode of action information

Studies suggest that innate and adaptive immune system is impacted by DBP exposure (Hansen et al. 2015). DBP is proposed to be metabolized to the monoester *in vitro*. This compound then is proposed to modulate cytokine secretion from both monocytes/macrophages and T cells.

The results from Couleau and colleagues (2015) suggest that some effects may occur through activation of the endocrine pathway. DBP also may regulate gene and protein expression of a variety of immune factors (e.g., cytokines) without impacting cell viability.

Immunomodulation by DBP also may occur through receptor-mediated effects on the inflammasome.

Rodent Data

Data from in vivo immunotoxicology or toxicology studies

DBP did not increase proliferative responses in lymph nodes of BALB/c mice at concentrations up to 20% (v/v in acetone; dermal route of exposure). Additionally, 10% DBP did not increase dendritic cell accumulation in draining lymph nodes (Dearman et al. 1996).

Wistar rats were fed a diet containing 0.5% or 5% DBP for 34-36 days. While no effect on absolute spleen weight was reported, a significant increase (1.8-fold) in relative spleen weight was reported at 5% DBP (Murakami et al. 1986).

Female BALB/cJ mice were subcutaneously exposed to ovalbumin (antigen) and 2-2000 μg/mL DBP. After the primary immunization, one or two booster shots were given to the mice. No effects on the IgG1 or IgE serum levels after either one or two booster shots were noted. A dose-dependent effect was observed on IgG1 serum levels; maximum responses were observed at 200 μg/ml (value not provided). No effect was noted on IgE serum levels (data not provided) (Larsen et al. 2002).

Thymic stromal lymphopoietin (TSLP) mRNA expression was significantly increased in BALB/c mouse ears 24 hours after exposure to DBP (in acetone, 1:1) (values not provided). An increase in TSLP protein levels was also measured at 24 hours (values not provided) (Larson et al. 2010; Schuepbach-Mallepell et al. 2013). DBP-induced induction of TSLP was strain dependent (BALB/c was more sensitive than C57Bl/6 mice). DBP also produced effects on TSLP in IL-1 receptor or apoptosis-associated speck-like protein containing a caspase recruitment domain deficient mice (Schuepbach-Mallepell et al. 2013).

In vitro data with cells or cell lines

DBP was cytotoxic to murine peritoneal exudate macrophages (PEM) after exposure to 50 or 100 μM for 24 hours. Annexin V and PI double stained cells (markers of apoptosis) were significantly increased after treatment with 100 μM DBP for 24 hours. Additionally, using trypan blue exclusion, a significant decrease in viable cells was reported after DBP exposure (LOAEL = 50 μM). Using two-color flow cytometry, DBP was shown to decrease expression of CD80, CD36, and major histocompatibility-II molecules on F4/80+ macrophages at 1 and 10 μM. Cytokine expression (IL-1β, IL-6, IL-12, and TNF-α) also were decreased at the same concentrations. Phagocytotic capacity of PEM to apoptotic thymocytes and *E. coli* was decreased after exposure to DBP when compared to controls (LOAEL = 1 μM). DBP exposure also decreased PEM immunogenicity to allogenic T cells (LOAEL = 1 μM) (Li et al. 2013).

DBP decreased cell viability of RAW 264.7 macrophages (LOAEL = 100 μM for 60 minutes) but did not increase cellular apoptosis (NOAEL = 1 mM for 60 minutes) (Naarala and Korpi 2009).

In RBL-2H3 mast cells sensitized with anti-dinitrophenyl monoclonal IgE, DBP potentiated β-hexosaminidase activity, which was used as a measurement of degranulation (LOAEL = 50 μM for 10 minutes). DBP did not induce degranulation in the cells that were not sensitized (NOAEL = 500 μM for 10 minutes) (Nakamura et al. 2002).

In PAM212 keratinocytes, 1% DBP increased relative expression of TSLP; maximal effect (values not provided) was observed at 36 hours post treatment (Larson et al. 2010). DBP-induced TSLP expression was associated with epidermal mouse skin and human abdominal skin transplanted on mice (Schuepbach-Mallepell et al. 2013).

Mode of action information

In vivo rodent studies suggest that DBP impacts the Th2 response. Inflammasome activation by DBP impacts TSLP expression and Th2 response.

In vitro studies suggest that while high doses of DBP induced macrophage apoptosis, moderate doses induced protein expression and production of cytokines. DBP also impacted the antigen-presenting capacity of macrophages.

References

Couleau N, Falla J, Beillerot A, Battaglia E, D'Innocenzo M, Plancon S, et al. 2015. Effects of Endocrine Disruptor Compounds, Alone or in Combination, on Human Macrophage-Like THP-1 Cell Response. PloS one 10:e0131428; doi:10.1371/journal.pone.0131428.

Dearman RJ, Cumberbatch M, Hilton J, Clowes HM, Fielding I, Heylings JR, et al. 1996. Influence of dibutyl phthalate on dermal sensitization to fluorescein isothiocyanate. Fundamental and applied toxicology : official journal of the Society of Toxicology 33: 24–30.

Gwinn MR, Whipkey DL, Tennant LB, Weston A. 2007. Gene expression profiling of di-n-butyl phthalate in normal human mammary epithelial cells. Journal of environmental pathology, toxicology and oncology : official organ of the International Society for Environmental Toxicology and Cancer 26: 51–61.

Hansen JF, Nielsen CH, Brorson MM, Frederiksen H, Hartoft-Nielsen ML, Rasmussen AK, et al. 2015. Influence of phthalates on in vitro innate and adaptive immune responses. PloS one 10:e0131168; doi:10.1371/journal.pone.0131168.

Kruger T, Cao Y, Kjaergaard SK, Knudsen LE, Bonefeld-Jorgensen EC. 2012. Effects of phthalates on the human corneal endothelial cell line B4G12. International journal of toxicology 31:364–71; doi:10.1177/1091581812449660.

Larsen ST, Lund RM, Nielsen GD, Thygesen P, Poulsen OM. 2002. Adjuvant effect of di-n-butyl-, di-n-octyl-, di-iso-nonyl- and di-iso-decyl phthalate in a subcutaneous injection model using BALB/c mice. Pharmacology & toxicology 91: 264–72.

Larson RP, Zimmerli SC, Comeau MR, Itano A, Omori M, Iseki M, et al. 2010. Dibutyl phthalate-induced thymic stromal lymphopoietin is required for Th2 contact hypersensitivity responses. Journal of immunology (Baltimore, Md : 1950) 184:2974–84; doi:10.4049/jimmunol.0803478.

Li L, Li HS, Song NN, Chen HM. 2013. The immunotoxicity of dibutyl phthalate on the macrophages in mice. Immunopharmacology and immunotoxicology 35:272–81; doi:10.3109/08923973.2013.768267.

Lourenco AC, Galbiati V, Corti D, Papale A, Martino-Andrade AJ, Corsini E. 2015. The plasticizer dibutyl phthalate (DBP) potentiates chemical allergen-induced THP-1 activation. Toxicology in vitro : an international journal published in association with BIBRA 29:2001–8; doi:10.1016/j.tiv.2015.08.011.

Murakami K, Nishiyama K, Higuti T. 1986. Toxicity of dibutyl phthalate and its metabolites in rats. Nihon eiseigaku zasshi Japanese journal of hygiene 41: 775–81.

Naarala J, Korpi A. 2009. Cell death and production of reactive oxygen species by murine macrophages after short term exposure to phthalates. Toxicology letters 188:157–60; doi:10.1016/j.toxlet.2009.04.001.

Nakamura R, Teshima R, Sawada J. 2002. Effect of dialkyl phthalates on the degranulation and Ca2+ response of RBL-2H3 mast cells. Immunology letters 80: 119–24.

Ni J, Zhang Z, Luo X, Xiao L, Wang N. 2016. Plasticizer DBP Activates NLRP3 Inflammasome through the P2X7 Receptor in HepG2 and L02 Cells. Journal of biochemical and molecular toxicology 30:178–85; doi:10.1002/jbt.21776.

Schuepbach-Mallepell S, Philippe V, Bruggen MC, Watanabe H, Roques S, Baldeschi C, et al. 2013. Antagonistic effect of the inflammasome on thymic stromal lymphopoietin expression in the skin. The Journal of allergy and clinical immunology 132:1348–57; doi:10.1016/j.jaci.2013.06.033.

Dichloroacetic Acid (DCAA) [CASRN 79-43-6]

Human Data

Data from epidemiology studies

No studies were identified.

In vitro data with cells or cell lines

A single study suggested that DCAA may produce immunosuppressive effects. Using a two-way mixed lymphocyte reaction, DCAA (LOAEL = 0.33 mM; lowest dose tested) increased IL-10 production and FOX P3 expression 11.4- and 4.5-fold, respectively (Eleftheriadis et al. 2013). DCAA (3.0 mM and 0.5 mM, respectively) increased IL-2 production after incubation for 16 hours and expression of the T-cell activation marker CD25 in Jurkat cells. Comparatively, no effect on CD69 expression (0.5 and 3.0 mM) was noted. IL-2 and IFN-γ mRNA expression was significantly increased after DCAA treatment (3.0 and 0.5 mM, respectively) (Pan et al. 2015).

DCAA (N/LOAEL = 0.1/1.0 mM) induced statistically significant increases in necrosis in PBMC, as shown by a decrease in PBMC cell size combined with an increase in cellular granulation. Statistically significant increases in the percentage of apoptotic cells were observed at similar concentrations of DCAA (N/LOAEL = 1.0/2.0 mM) (Michalowicz et al. 2015).

Mode of action information

T-cell activation was one proposed mode of action for DCAA. Increased IL-10 production, combined with increased FOX P3 expression, is proposed to increase regulatory T-cell differentiation which may lead to increased IL-10 production. Additionally, DCAA increased expression of T-cell activation markers in Jurkat cells.

Apoptosis was proposed be associated with a variety of mechanisms including ROS generation, alterations in mitochondrial transmembrane potential, and activation of caspase activity.

Rodent Data

Data from in vivo immunotoxicology or toxicology studies

In a 90-day drinking water study (0, 50, 500 and 5000 ppm (w/v) DCAA) with male Sprague-Dawley rats, a significant increase in relative spleen weight was noted at 5000 ppm (0.25% vs. 0.21%). No consistent effects on T cell-dependent anti-keyhole limpet hemocyanin IgG antibody production (measured by ELISA), delayed hypersensitivity to bovine serum albumin, NK cell cytotoxicity, or production of peritoneal macrophage-derived PGE2 or spleen lymphocyte-derived IL-2 were detected at tested doses (data not shown in paper) (Exon et al. 1986; Mather et al. 1990).

In autoimmune-prone MRL +/+ female mice, 0.5 mg/mL DCAA (provided *ad libitum* in drinking water for 12 weeks) significantly increased serum IgG (32%) and IgM (30%) levels. DCAA significantly decreased IL-10 (34%) and KC chemokine (31%) in liver extracts from

MRL +/+ mice. Comparatively, a significant increase in serum IgG3 levels (27%) was observed in wild-type B6C3F1 after DCAA exposure. In liver extracts from treated B6C3F1 mice, DCAA significantly increased IL-4 (400%), IL-5 (33%), IL-6 (53%), IL-10 (25%), IL-12 (32%), KC chemokine (18%), GM-CSF (42%), G-CSF (56%), and IFN-γ (45%) compared to controls. Compared to isolated MRL +/+ splenic lymphocytes from controls, DCAA decreased IL-4 and IL-10 secretion in MRL+/+ treated mice. DCAA decreased IL-4 and increased IFN-γ secretion from splenic lymphocytes from treated B6C3F1 mice when compared to controls (values not provided). DCAA also significantly decreased IL-4 and IL-2 secretion and significantly increased IL-5, IFN-γ, and GM-CSF secretion from B6C3F1 isolated splenic lymphocytes when compared to secretion from MRL +/+ isolated splenic lymphocytes from treated animals (values not provided) (Cai et al. 2007).

In vitro data with cells or cell lines

No studies were identified.

Mode of action information

DCAA-induced increase of p53 accumulation has been proposed to lead to increased formation of cells in G2-M phase (Staneviciute et al. 2016).

References

Cai P, Boor PJ, Khan MF, Kaphalia BS, Ansari GAS, Konig R. 2007. Immuno- and hepato-toxicity of dichloroacetic acid in MRL(+/+) and B(6)C(3)F(1) mice. J Immunotoxicol 4:107–115; doi:10.1080/15476910701337225.

Eleftheriadis T, Pissas G, Karioti A, Antoniadi G, Antoniadis N, Liakopoulos V, et al. 2013. Dichloroacetate at therapeutic concentration alters glucose metabolism and induces regulatory T-cell differentiation in alloreactive human lymphocytes. J Basic Clin Physiol Pharmacol 24:271–276; doi:10.1515/jbcpp-2013-0001.

Exon JH, Koller LD, Talcott, P.A., O'Reilly CA, Henningsen GM. 1986. Immunotoxicity Testing: An Economical Multiple-Assay Approach. Fundamental and Applied Toxicology 7: 387–397.

Mather GG, Exon JH, Koller LD. 1990. Subchronic 90 day toxicity of dichloroacetic and trichloroacetic acid in rats. Toxicology 64: 71–80.

Michalowicz J, Wroblewski W, Mokra K, Macczak A, Kwiatkowska M. 2015. Comparative study of the effect of chloro-, dichloro-, bromo-, and dibromoacetic acid on necrotic, apoptotic and morphological changes in human peripheral blood mononuclear cells (in vitro study). Toxicol In Vitro 29:1416–1424; doi:10.1016/j.tiv.2015.05.021.

Pan Y, Wei X, Hao W. 2015. Trichloroethylene and Its Oxidative Metabolites Enhance the Activated State and Th1 Cytokine Gene Expression in Jurkat Cells. Int J Environ Res Public Health 12:10575–10586; doi:10.3390/ijerph120910575.

Staneviciute J, Urboniene D, Valanciute A, Balnyte I, Vitkauskiene A, Grigaleviciene B, Stakisaitis D. 2016. The effect of dicloroacetate on male rat thymus and on thymocyte cell cycle. Int J Immunopathol Pharmacol 29:818-822.

Diethylstilbestrol (DES) [CASRN 56-53-1]

Human Data

Data from epidemiology studies

Male and female offspring of pregnant women given DES doses from the seventh week to 34th week of pregnancy, were interviewed about immune-related health problems. A total of 549 DES-exposed offspring and 487 placebo-exposed offspring participated in the study. Rates of allergy-related health problems (e.g., asthma, drug allergy, hives) were similar between DES- and placebo-offspring. Infection (e.g., shingles, flu) and autoimmune disease (e.g., diabetes, rheumatoid arthritis) also were similar between the two groups (Baird et al. 1996).

The frequency of any autoimmune disease in women exposed to DES *in utero* (n = 1711) was higher than the frequency observed in control women. The overall frequency was 28.6 per 1000 women compared to 16.3 per 1000 women. Hashimoto's thyroiditis was significantly more prevalent (relative prevalence = 5.4) in exposed women compared to controls (Noller et al. 1988).

Increased incidence of asthma, arthritis, and diabetes mellitus was reported in sons and daughters exposed to DES *in utero* when compared to unexposed individuals. Additionally, the number of respiratory tract conditions (e.g., colds) was increased in the exposed population vs. the unexposed population (Wingard and Turiel 1988).

In vitro data with cells or cell lines

Lymphocyte NK activity (assessed using chromium release from K562 cells) from 12 patients exposed to DES *in utero* was greater than observed from controls; however, effects were not significant. No effects on adherent cells were noted (Ford et al. 1983). Comparatively, DES dose-dependently inhibited lysis of K562 cells in PBMCs obtained from 12 patients. At the highest concentration tested (100 µM), an 82% reduction in activity was observed compared to control samples (Ablin et al. 1988b, 1988a)

Responses to 0.125 µg/mL PHA (as measured by uptake of radiolabeled thymidine) was significantly greater in peripheral blood monocytes from women exposed to DES *in utero* compared to controls (88.6×10^3 vs. 44.0×10^3 cpm; $p < 0.002$). Maximal blastogenic response to PHA in lymphocytes from DES-exposed women was observed at 0.125 µg/mL while it was observed at 0.25-0.50 µg/mL in controls (Ways et al. 1987).

Mode of action information

DES inhibits the lytic activity of human NK cells (Kalland and Campbell 1984).

Rodent Data

Data from in vivo immunotoxicology or toxicology studies

C57BL/6 dams were orally administered 48 µg/kg DES from GD 14-16 and then sacrificed on GD 18. Fetal thymic weight and cellularity were significantly decreased (44% and 51%, respectively) in treated animals. Relative fetal thymic weight was decreased 28% when compared to controls. The percentage of thymocytes in the CD4-8- and CD4-8+ populations were increased 87% and 138%, respectively. Comparatively, CD4+8+ thymocyte population was decreased 12%. Increased apoptosis of CD4+8+, CD4-8+, and CD4-8- thymocytes also was observed (Besteman et al. 2005).

Male and female CD-1 mice were subcutaneously injected with 5, 15, or 30 µg/kg DES, four times on alternate days. Relative thymic weight (LOAEL = 30 µg/kg) was decreased, and absolute and relative splenic weights (LOAELs = 15 µg/kg) were increased in female mice. A similar effect in male mice was not observed. Relative expression of thymocyte populations (e.g., CD4-8-) were not affected in males or females. However, an increase in the number of total apoptotic and decrease in the number of live CD4+8+ and CD4+8- thymocytes was observed in male and female mice. Additionally, an increase in the number of total apoptotic and decrease in the number of live CD4-8- cells were observed in females. Increased proliferative response to ConA, LPS, or PMA was observed in splenic lymphocytes isolated from female mice treated with 5 µg/kg DES. At higher doses, a trend for decreased proliferation was observed in female splenic lymphocytes. Proliferative responses by splenic lymphocytes were only modulated in response to ConA at 15 µg/kg DES (Calemine et al. 2002).

Female mice (strain not provided) were administered (route not provided) 0.2, 2.0, or 8.0 mg/kg DES for 5 days. Antibody response to sheep erythrocytes and LPS were decreased 15% to 45% (LOAELs = 2.0 mg/kg). Delayed hypersensitivity response to keyhole lymphet hemocyanin was similar to controls when mice were exposed to DES before sensitization. However, when mice were exposed to DES after sensitization and before challenge a decrease in response was observed (LOAEL = 2 mg/kg). The percentage of splenic T lymphocytes was decreased 25% at the highest dose tested. No effect on the percentage of splenic B lymphocytes was observed. Splenic lymphoproliferative response to PHA and ConA were decreased (>30%) at all tested doses. Responses to *Staphylococcus* enterotoxin A were increased at 0.2 mg/kg and decreased at higher doses, while responses to LPS were increased at 0.2 and 2.0 mg/kg and decreased at 8.0 mg/kg. MLC responses also were decreased (LOAEL = 2 mg/kg). Suppressor cell activity was decreased after exposure to 8 mg/kg DES (Luster et al. 1980).

Differential effects on the immune system were observed in female NMRI mice depending on the time of DES exposure. Thymus weights were increased in 56-day-old mice that were subcutaneously injected with 5 µg from PND 1-5, 6-10, or 30-34 (1.2- to 1.4-fold). Comparatively, thymus weight was decreased in mice subcutaneously injected with DES from PND 48-52 (29%). A dose-related effect on thymus weight was observed in mice treated from PND 1-5; no effects on absolute or relative spleen weight were noted. Differences in thymus weight also were noted depending on when the mice were killed after treatment. Four days after treatment, thymus weights were decreased in all test groups. However, 4 to 8 weeks after

treatment showed an increase in thymus weight in mice treated on PND 1-5 and weights similar to controls in other treatment groups. DES treatment on PND 1-5 also reduced the number of cells in S-phase in the thymus (Forsberg 1996).

C57BL/6 mice were treated with DES once *in utero* and/or once at 12-16 months of age via subcutaneous injection. Increased secretion of IFNγ was observed in splenic lymphocytes obtained from mice exposed to DES *in utero* and as adults. Increased IFNγ also was observed when splenocytes were stimulated with anti-CD3 antibodies. This increase was not observed in other treatment conditions (data in graphs). An increase in IFNγ production also was observed in T-cells from mice exposed to DES *in utero* and as adults (Karpuzoglu-Sahin et al. 2001).

In vitro data with cells or cell lines

DES stimulated IL-1 production from peritoneal exudate macrophages at concentrations ranging from 0.01 to 1 μM; the maximal response was observed at 0.1 μM. DES (0.1 μM) also significantly increased production of IL-6 (1.7-fold), IL-12 (9.5-fold) TNF-α (3.1-fold), and macrophage chemotactic protein 1 (7.2-fold), and surface expression of CD86 (1.6-fold). DES also increased proliferative responses (8.6-fold) and IL-2 production (5.6-fold) observed when macrophages were incubated with purified T cells. Anti-MHC-II, -CD-80, and -CD86 blocked effects produced by DES (Yamashita et al. 2005).

DES increased IgE levels in male BALB/c mouse splenocytes at concentrations greater than 1 μM. Comparatively, DES had no effect on IgM, IgG, or IgA levels at concentrations up to 1 mM (Han et al. 2002).

Mode of action information

DES-induced thymic atrophy was proposed to be due, in part, to estrogen-related thymocyte apoptosis (Besteman et al. 2005; Fenaux et al. 2004). Brown and colleagues suggested that DES exposure upregulates TNF family members, which leads to altered T-cell development. This alteration was suggested to lead to thymic atrophy (Brown et al. 2006). Direct effects on T lymphocytes also may occur (Luster et al. 1980).

In mice, DES exposure was associated with down-regulation of gene expression in the TCR complex, and the TCR and CD28 signaling pathways. Genes in the B-cell receptor signaling pathway, antigen presentation, and dendritic cell pathways also was altered by DES exposure. It was proposed that DES dysregulation of T-cell development plays a role in thymus effects (Frawley et al. 2011). Alterations of microRNA expression also has been proposed as playing a role in the immunotoxic effects produced by DES (Singh et al. 2015).

Additional proposed modes of action on the immune system include effects on adherent suppressor cells, modulation of NK activity by interfering with bone marrow lymphoid precursors, and modulation of the mononuclear phagocyte system (Dean et al. 1986; Forsberg 1984; Kalland 1984; Luster et al. 1980).

References

Ablin RJ, Bartkus JM, Gonder MJ. 1988a. In vitro effects of diethylstilboestrol and the LHRH analogue leuprolide on natural killer cell activity. Immunopharmacology 15: 95–101.
Ablin RJ, Gonder MJ, Bartkus JM. 1988b. Leuprolide vs. diethylstilboestrol: effect on natural killer cells. Anticancer research 8: 73–6.
Baird DD, Wilcox AJ, Herbst AL. 1996. Self-reported allergy, infection, and autoimmune diseases among men and women exposed in utero to diethylstilbestrol. Journal of Clinical Epidemiology 49:263–266; doi:10.1016/0895-4356(95)00521-8.
Besteman EG, Zimmerman KL, Holladay SD. 2005. Diethylstilbestrol (DES)-induced fetal thymic atrophy in C57BL/6 mice: inhibited thymocyte differentiation and increased apoptotic cell death. International journal of toxicology 24:231–9; doi:10.1080/10915810591000703.
Brown N, Nagarkatti M, Nagarkatti PS. 2006. Induction of apoptosis in murine fetal thymocytes following perinatal exposure to diethylstilbestrol. International journal of toxicology 25:9–15; doi:10.1080/10915810500488353.
Calemine JB, Gogal RM Jr, Lengi A, Sponenberg P, Ahmed SA. 2002. Immunomodulation by diethylstilbestrol is dose and gender related: effects on thymocyte apoptosis and mitogen-induced proliferation. Toxicology 178: 101–18.

Dean JH, Lauer LD, Murray MJ, Luster MI, Neptun D, Adams DO. 1986. Functions of mononuclear phagocytes in mice exposed to diethylstilbestrol: a model of aberrant macrophage development. Cellular immunology 102: 315–22.
Fenaux JB, Gogal RM Jr, Ahmed SA. 2004. Diethylstilbestrol exposure during fetal development affects thymus: studies in fourteen-month-old mice. Journal of reproductive immunology 64:75–90; doi:10.1016/j.jri.2004.08.003.
Ford CD, Johnson GH, Smith WG. 1983. Natural killer cells in in utero diethylstilbesterol-exposed patients. Gynecologic oncology 16: 400–4.
Forsberg JG. 1984. Short-term and long-term effects of estrogen on lymphoid tissues and lymphoid cells with some remarks on the significance for carcinogenesis. Archives of toxicology 55: 79–90.
Forsberg JG. 1996. The different responses of the female mouse thymus to estrogen after treatment of neonatal, prepubertal, and adult animals. Acta anatomica 157: 275–90.
Frawley R, White K Jr, Brown R, Musgrove D, Walker N, Germolec D. 2011. Gene expression alterations in immune system pathways in the thymus after exposure to immunosuppressive chemicals. Environmental health perspectives 119:371–6; doi:10.1289/ehp.1002358.

Han D, Denison MS, Tachibana H, Yamada K. 2002. Effects of estrogenic compounds on immunoglobulin production by mouse splenocytes. Biological & pharmaceutical bulletin 25: 1263–7.
Kalland T. 1984. Exposure of neonatal female mice to diethylstilbestrol persistently impairs NK activity through reduction of effector cells at the bone marrow level. Immunopharmacology 7: 127–34.

Kalland T, Campbell T. 1984. Effects of diethylstilbestrol on human natural killer cells in vitro. Immunopharmacology 8: 19–25.

Karpuzoglu-Sahin E, Hissong BD, Ansar Ahmed S. 2001. Interferon-gamma levels are upregulated by 17-beta-estradiol and diethylstilbestrol. Journal of reproductive immunology 52: 113–27.

Luster MI, Boorman GA, Dean JH, Luebke RW, Lawson LD. 1980. The effect of adult exposure to diethylstilbestrol in the mouse: alterations in immunological functions. Journal of the Reticuloendothelial Society 28: 561–9.

Noller KL, Blair PB, O'Brien PC, Melton LJ, Offord JR, Kaufman RH, et al. 1988. Increased occurrence of autoimmune disease among women exposed in utero to diethylstilbestrol. Fertil Steril 49: 1080–1082.

Singh NP, Abbas IK, Menard M, Singh UP, Zhang J, Nagarkatti P, et al. 2015. Exposure to diethylstilbestrol during pregnancy modulates microRNA expression profile in mothers and fetuses reflecting oncogenic and immunological changes. Molecular pharmacology 87:842–54; doi:10.1124/mol.114.096743.

Ways SC, Mortola JF, Zvaifler NJ, Weiss RJ, Yen SS. 1987. Alterations in immune responsiveness in women exposed to diethylstilbestrol in utero. Fertil Steril 48: 193–197.

Wingard DL, Turiel J. 1988. Long-term effects of exposure to diethylstilbestrol. West J Med 149: 551–554.

Yamashita U, Sugiura T, Yoshida Y, Kuroda E. 2005. Effect of endocrine disrupters on macrophage functions in vitro. Journal of UOEH 27: 1–10.

Ethylene Dibromide (EDB) [CASRN 106-93-4]

Human Data

Data from epidemiology studies

The prevalence of adult-onset asthma, in relation to lifetime pesticide use, were assessed using data from the Agricultural Health Study (19,704 male farmers). Adult-onset asthma was reported in 441 individuals; 127 classified as allergic and 314 classified as non-allergic. EDB exposure was positively associated with allergic asthma (OR: 2.07 [1.02-4.20]) (Hoppin et al. 2009).

In vitro data with cells or cell lines

No data were located.

Mode of action information

No data were located.

Rodent Data

Data from in vivo immunotoxicology or toxicology studies

Female B6C3F1 mice were intragastrically treated with 100, 125, 160, or 200 mg/kg EDB for 14 days. Relative thymus and spleen weights were decreased in a dose-related manner (LOAEL = 200 mg/kg). Comparatively, relative liver and kidney weights were increased at higher doses (LOAEL = 125 and 160 mg/kg, respectively). Significant increases in white blood cells (LOAEL = 200 mg/kg) and neutrophils (LOAEL = 160 mg/kg) were noted. Host resistance to influenza A2, *Listeria monocytogenes*, and herpes simplex virus types 1 and 2 was not significantly affected by EDB exposure. The total number of resident peritoneal exudate cells were significantly increased in EDB-treated mice (LOAEL = 160 mg/kg). However, the percentage of cell types present in the exudates were similar to those observed in control exudates (macrophages: 53%; lymphocytes: 47%). Phagocytosis of radiolabeled chicken red blood cells was increased in peritoneal macrophages obtained from EDB-treated mice (187% of control; LOAEL = 125 mg/kg). Splenic NK cell activity was evaluated in animals treated with 100, 125, or 160 mg/kg; a significant decrease in activity was observed at 160 mg/kg. The number of viable cells in the spleen decreased at 125, 160, and 200 mg/kg (not significant), while a significant increase in the number of anti-SE PFC/10^6 viable spleen cells was significantly increased at 160 mg/kg. Splenic lymphocyte responses to allogenic spleen cells, PHA and ConA, but not LPS, were significantly decreased at 125 and 160 mg/kg (Ratajczak et al. 1994).

Female B6C3F1 were intragastrically treated with 31.25, 62.5, or 125 mg/kg EDB for 5 days per week for 12 weeks. No effect on white blood cell numbers, or the percentage of neutrophils or lymphocytes were noted at the doses tested. Splenic lymphocyte responses to PHA and LPS were significantly decreased at the highest dose tested (data not provided) (Ratajczak et al. 1995).

Relative spleen weights were not significantly affected in male Sprague-Dawley rats inhalationally exposed to EDB 7 hours per day, 5 days per week, for 30 days. However, relative liver weights were increased at the highest dose tested (LOAEL = 455 ppm) (Igwe et al. 1986).

In vitro data with cells or cell lines

No data were located.

Mode of action information

No data were located.

References

Hoppin JA, Umbach DM, London SJ, Henneberger PK, Kullman GJ, Coble J, et al. 2009. Pesticide use and adult-onset asthma among male farmers in the Agricultural Health Study. The European respiratory journal 34:1296–303; doi:10.1183/09031936.00005509.

Igwe OJ, Que Hee SS, Wagner WD. 1986. Interaction between 1,2-dichloroethane and disulfiram. I. Toxicologic effects. Fundamental and applied toxicology : official journal of the Society of Toxicology 6: 733–46.

Ratajczak HV, Aranyi C, Bradof JN, Barbera P, Fugmann R, Fenters JD, et al. 1994. Ethylene dibromide: evidence of systemic and immunologic toxicity without impairment of in vivo host defenses. In vivo (Athens, Greece) 8: 879–84.

Ratajczak HV, Thomas PT, Gerhart J, Sothern RB. 1995. Immunotoxicologic effects of ethylene dibromide in the mouse and their modulation by the estrous cycle. In vivo (Athens, Greece) 9: 299–304.

Glycidol [CASRN 556-52-5]

Human Data

Data from epidemiology studies

No data were located.

In vitro data with cells or cell lines

No data were located.

Mode of action information

No data were located.

Rodent Data

Data from in vivo immunotoxicology or toxicology studies

Of 10 F344/N female rats that received 400 mg/kg glycidol for 13-weeks (via gavage), lymphoid necrosis of the thymus was observed in nine (Irwin et al. 1996; National Toxicology Program 1990). Enlarged spleen were observed in haploinsufficient $p16^{Ink4a}/p19^{Arf}$ male mice treated with 200 mg/kg glycidol for 40 weeks via gavage (National Toxicology Program 2007).

Increased splenic fibrosis incidence was reported in male and female F344/N rats gavaged with 37.5 and 75 mg/kg glycidol for 2 years. In males, splenic fibrosis incidences were 26% in controls, 68% in rats treated with 37.5 mg/kg, and 56% in rats treated with 75 mg/kg. In females, splenic fibrosis incidences were 6%, 29%, and 40% for control, 37.5 mg/kg rats and 75 mg/kg rats, respectively (National Toxicology Program 1990).

In female B6C3F1 mice administered glycidol via gastric intubation (25, 125, and 250 mg/kg) for 14 days, no effect on spleen or thymus weights, or leukocyte or lymphocytes counts were reported. To assess AFC response, treated mice were intravenously exposed to sheep erythrocytes on day 11 and spleen IgM AFC response was measured 4 days later. At the highest treatment dose, there was a 31% reduction in specific activity. When expressed as total spleen activity, significant decreases were noted at 125 and 250 mg/kg (29% and 41%, respectively). Splenic T-cell proliferation, in response to 10 µg/mL ConA was significantly decreased (16% and 26%, respectively) in splenocytes obtained from mice treated with 125 and 250 mg/kg glycidol. B-cell proliferation, in response to Il-4 or Il-4 and goat anti-mouse IgM F(ab')2, was only decreased in splenocytes obtained from mice treated with 125 mg/kg glycidol (13% and 16%, respectively). Comparatively, proliferation in response to goat anti-mouse IgM F(ab')2 was decreased in splenocytes from mice treated with 125 and 250 mg/kg glycidol (30-32%). While glycidol had no effect on lymphocyte blastogenesis (as assessed by splenocyte proliferative response) alone, in the presence of allogenic DBA/2 spleen cells a 25% decrease in response was noted at the middle dose only. NK cell activity in spleens was decreased at two ratios of effector:target ratios (100:1 and 50:1); the LOAELs at both ratios were 125 and 250 mg/kg, respectively. Using flow cytometry, the number and percent of B lymphocytes, T-lymphocytes,

$CD4^+CD8^-$, $CD4^-CD8^+$, and $CD4^-CD8^-$ cells from spleens isolated from treated mice were quantified. The total number of spleen cells, B lymphocytes, and $CD4^+CD8^-$ were significantly decreased at 250 mg/kg. The LOAEL also was 250 mg/kg when the percent values of B and T lymphocytes were assessed (Guo et al. 2000).

In vitro data with cells or cell lines

To further assess the effect of glycidol on the immune function, Guo and colleagues (2000) conducted a set of *ex vivo* assays. Glycidol inhibited cytotoxic T cell activity in spleens obtained from mice administered glycidol via gastric intubation (25, 125, and 250 mg/kg) for 14 days. Splenocytes were sensitized with mitomycin C-exposed P815 mastocytoma cells, and co- cultured with labeled P815 cells at a variety of effector:target ratios. At an effector:target ratio of 25:1 and 0.75:1, glycidol inhibited CTL activity at a 25 mg/kg when compared to vehicle (53.8 vs. 31.5, and 8.8 vs. 2.1, respectively). At a ratio of 12.5:1, CTL activity was decreased significantly (39%) in spleens from mice treated with 125 mg/kg glycidol (Guo et al. 2000).

Resident macrophage activity (in the presence of macrophage stimulators) was assessed in mice treated with glycidol via gastric intubation (25, 125, and 250 mg/kg) for 14 days. Increased cytotoxicity was only observed after treatment with 25 mg/kg glycidol in the presence or absence of macrophage stimulators (1.7- to 2.5-fold increase) (Guo et al. 2000).

Host resistance to B16F10 melanoma cells, *Listeria monocytogenes* and *Streptococcus pneumoniae* were assessed in mice treated with glycidol via gastric intubation (25, 125, and 250 mg/kg) for 14 days. Glycidol increased pulmonary tumor formation in mice treated with B16F10 melanoma cells (LOAEL = 125 mg/kg). No effect on host resistance was noted at the three challenge levels of *Listera monocytogenes* (1, 2, or 4 × 10^4 CFU/mouse). At thechallenge level 5.52 × 10^7 CFU *Streptococcus phenumoniae*/mouse, increased host resistance was observed in the 250-mg/kg glycidol treated mice (Guo et al. 2000).

Mode of action information

Studies suggest that glycidol modulates B-cell function, and NK cell and macrophage activities (Guo et al. 2000).

References

Guo TL, McCay JA, Brown RD, Musgrove DL, Butterworth L, Munson AE, et al. 2000. Glycidol modulation of the immune responses in female B6C3F1 mice. Drug and chemical toxicology 23:433–57; doi:10.1081/dct-100100127.

Irwin RD, Eustis SL, Stefanski S, Haseman JK. 1996. Carcinogenicity of glycidol in F344 rats and B6C3F1 mice. Journal of applied toxicology : JAT 16:201–9; doi:10.1002/(sici)1099-1263(199605)16:3<201::aid-jat333>3.0.co;2-0.

National Toxicology Program. 1990. NTP Toxicology and Carcinogenesis Studies of Glycidol (CAS No. 556-52-5) In F344/N Rats and B6C3F1 Mice (Gavage Studies). Natl Toxicol Program Tech Rep Ser 374: 1–229.

National Toxicology Program. 2007. Toxicology and carcinogenesis study of glycidol (CAS No. 556-52-5) in genetically modified haploinsufficient p16(Ink4a)/p19(Arf) mice (gavage study). National Toxicology Program genetically modified model report 1–81.

Hydrocortisone (HC) [CASRN 50-23-7]

Human Data

Data from epidemiology studies

No data were located.

In vitro data with cells or cell lines

The effect of HC on IL-4-induced IgE production was measured in PBMCs isolated from healthy volunteers. HC induced an ~20 fold increase in IgE production at a LOEL of 1×10^{-7} M. HC did not have any effect on IgE production in the absence of IL-4 (data not shown) (Nüsslein et al. 1994).

Blood samples from healthy adults were pre-treated with 30 µg/dL HC (identified as cortisol); INF production was then stimulated with Newcastle disease virus. HC decreased IFN-α response by 50-60% (data in graph) (Reissland and Wandinger 1999).

Mode of action information

Keh and colleagues (2003) reported that in septic shock patients, HC attenuated inflammatory and anti-inflammatory responses without inducing immunosuppression.

Rodent Data

Data from in vivo immunotoxicology or toxicology studies

Swiss inbred mice were intraperitoneally injected with 0, 1.5, 5 or 15 mg/kg of HC. Forty-eight hours later, there was a significant decrease in thymus weight at 15 mg/kg (data in graph). To test the effect of HC on delayed hypersensitivity, mice were immunized with sheep erythrocytes in FCA, challenged in the footpad on day 5, and treated simultaneously with increasing amounts of HC. The mice received another injection of HC two hours before measuring 24-hour footpad swelling. 5 and 15 mg/kg HC suppressed footpad swelling (data in graph). Glucocorticoid-induced leukopenia and monocytopenia was evaluated in mice 2.5 hours after intravenous injection with HC. The numbers of circulating nucleated and monocytic cells was maximally decreased at the lowest dose tested (1.5 mg/kg), with the number of both cell types increasing with increasing dose (data in graph). A plasma transfer study found that 2.5 hours after transfer, the plasma of HC treated mice raised the number of nucleated cells in saline treated acceptor mice by 46%. To evaluate feedback-inhibition, mice were injected (route not specified) with 5 mg/kg HC for four days and examined 7 or 11 days (data not shown) after the last injection. At day 7, HC had no effect on delayed hypersensitivity, serum corticosterone, or numbers of circulating nucleated and monocytic cells (data in graphs) (Van Dijk et al. 1979).

In a trio of studies by El Fouhil and colleagues (El Fouhil et al. 1993a, 1993b; El Fouhil and Turkall 1993), immunologically immature rats were treated subcutaneously with 400 mg/M^2/day HC, administered on alternate days from PND 7 to PND 19. At two days after the last treatment (PND 21), thymus and spleen weights were decreased (71 and 28%) compared to vehicle control,

but at PND 42 organ weights were increased (18 and 7%). Leucocytosis was increased in PND 21 and 42 rats (12 and 24%), with a decrease in IgM concentration in serum (45 and 15%). At PND 21 there was a 46% decrease in the percentage of lymphocytes, which resolved by PND 42 (El Fouhil and Turkall 1993). On PND 21, splenic white pulp was largely depleted of small lymphocytes. There were no distinct periartiolar lymphoid sheaths and no primary follicles. The number of T cells surrounding the central arteriole was decreased (data not shown). By PND 42, the pulp appeared normal (El Fouhil et al. 1993a). On PND 21, the outer cortex of mesenteric lymph nodes was found to be depleted of small lymphocytes and primary follicles, and neither cortical expansion nor capsular indentations were detected. There was a marked depletion of B lymphocytes, which were more or less discrete and did not aggregate to form follicles. There was no apparent change in T lymphocytes. On PND 42, the lymph nodes were comparable between HC treated and control rats (El Fouhil et al. 1993b).

HC (1.5 mg intraperitoneally administered) decreased formation of splenic anti-sheep erythrocyte (4×10^7 sheep erythrocytes) PFC in female BALB/c mice (data in graph). HC did not affect IgM-PFC or IgG-PFC response or serum antibody titers (data in graphs) (Jokay et al. 1980).

In vitro data with cells or cell lines

No data were located.

Mode of action information

No data were located.

References

El Fouhil AFI, Iskander FA, Turkall RM. 1993a. Effect of Alternate-Day Hydrocortisone Therapy on the Immunologically Immature Rat. II: Changes in T- and B-Cell Areas in Spleen. Toxicologic Pathology 21:383–390; doi:10.1177/019262339302100406.

El Fouhil AFI, Iskander FA, Turkall RM. 1993b. Effect of Alternate-Day Hydrocortisone Therapy on the Immunologically Immature Rat. III: Changes in T- and B-Cell Areas in Lymph Nodes. Toxicologic Pathology 21:391–396; doi:10.1177/019262339302100407.

El Fouhil AFI, Turkall RM. 1993. Effect of Alternate-Day Hydrocortisone Therapy on the Immunologically Immature Rat. I: Effect on Blood Cell Count, Immunoglobulin Concentrations, and Body and Organ Weights. Toxicologic Pathology 21:377–382; doi:10.1177/019262339302100405.

Jokay I, Kelemenics K, Karczag E, Foldes I. 1980. Interactions of glucocorticoids and heparin on the humoral immune response of mice. Immunobiology 157:390–400; doi:10.1016/s0171-2985(80)80008-8.

Keh D, Boehnke T, Weber-Cartens S, Schulz C, Ahlers O, Bercker S, et al. 2003. Immunologic and Hemodynamic Effects of “Low-Dose” Hydrocortisone in Septic Shock: A Double-Blind, Randomized, Placebo-controlled, Crossover Study. American Journal of Respiratory and Critical Care Medicine 167:512–520; doi:10.1164/rccm.200205-446OC.

Nüsslein HG, Weber G, Kalden JR. 1994. Synthetic glucocorticoids potentiate IgE synthesis.: Influence of steroid and nonsteroid hormones on human in vitro IgE secretion. Allergy 49:365–370; doi:10.1111/j.1398-9995.1994.tb02283.x.

Reissland P, Wandinger KP. 1999. Increased Cortisol Levels in Human Umbilical Cord Blood Inhibit Interferon Alpha Production of Neonates. Immunobiology 200:227–233; doi:10.1016/S0171-2985(99)80072-2.

Van Dijk H, Bloksma N, Rademaker PM, Schouten WJ, Willers JM. 1979. Differential potencies of corticosterone and hydrocortisone in immune and immune-related processes in the mouse. International Journal of Immunopharmacology 1:285–292; doi:10.1016/0192-0561(79)90004-3.

Indomethacin [CASRN 53-86-1]

Human Data

Data from epidemiology studies

No data were located.

In vitro data with cells or cell lines

Heparinized whole blood, from healthy adult volunteers, was incubated with 1 to 50 µM indomethacin for 1 hour prior to stimulation with LPS. A significant increase in IL-6 expression was only noted at 50 µM indomethacin (129.7%). Comparatively, a dose-dependent increase in TNF-α was observed, and at 50 µM the number of TNF-α positive cells had doubled (204.7%) (Hartel et al. 2004).

Human PBMC were treated with 1, 10 or 100 µM indomethacin. At all tested doses, indomethacin decreased LPS-induced PGE2 synthesis to near 0%; the calculated IC50 was 0.039 µM (data in graph). Indomethacin also decreased IgG and IgM production (data in graph) at all doses tested. Indomethacin up-regulated IL-2 production and down-regulated IL-6 production in treated PBMC (data not shown). Increased PHA-, anti-CD3, and IL-2-induced lymphocyte proliferation was reported after indomethacin exposure. NK activity (against K562 target cells) was increased at 1 (1.5-fold) and 10 (1.5-fold) µM. A significant effect on LAK cell activity was not observed at 50 µM. Co-incubation of PBMCs with IL-2 and indomethacin caused an increase in IFN-γ production by LAK cells at 1, 10 or 100 µM (data not shown) (Tanaka et al. 1998).

Indomethacin (5.6 µM) increased proliferation of PHA- and ConA-stimulated lymphocytes (in mononuclear cell cultures) (data in graph). The effect was only observed at suboptimal concentrations of PHA and ConA. The observed increased proliferation was lost at optimal and supraoptimal concentrations. Additional testing showed indomethacin increased PHA-stimulated lymphocyte proliferation in a dose-dependent manner (LOEL = 0.04 µM). Removal of adherent cells from the culture negated the stimulatory effect produced by indomethacin. Indomethacin did not affect cell viability, but increased incorporation of tritiated thymidine in a dose- dependent manner (Jawad and Rogers 1984).

Mode of action information

No data were located.

Rodent Data

Data from in vivo immunotoxicology or toxicology studies

Female C57BL/6 mice were orally administered 5 mg/kg indomethacin for 4 days. Animals were then immunized with sheep erythrocytes and then serum hemagglutination and AFC titers were assessed 4 and 8 days, or 6 days later, respectively. Indomethacin decreased both titers by approximately 40% (data in graphs). Indomethacin also decreased ConA- and LPS-induced

stimulation of lymphocyte proliferation (data in graphs). Incubation of indomethacin (3 μM) with LPS-stimulated lymphocytes isolated from indomethacin-treated mice decreased proliferation (Barasoain et al. 1980).

Female B6C3F1 mice were subcutaneously injected with 1, 2, or 4 mg/kg indomethacin for 6 days. Studies were conducted on mice 3 days after final treatment. No effect on thymus weight was reported, while 4 mg/kg indomethacin caused a 38% increase in spleen weight. A dose-dependent increase in splenic lymphocyte proliferation (10%-80%) was observed in non-stimulated cultures. Increased proliferation was observed in LPS-stimulated cultures from mice treated with 1 or 2 mg/kg indomethacin (13% and 22%, respectively), while a decrease was observed at the highest indomethacin dose. Comparatively, decreased proliferation was observed in PHA-, ConA-, or MLC-treated splenic cultures from indomethacin treated mice. Increased formation of PFC/10^6 splenocytes also were observed in treated mice (149% increase at 4 mg/kg). Indomethacin did not affect macrophage-induced inhibition of tumor cell growth (MBL-2), but did increase phagocytosis of sheep erythrocytes. Host resistance to *Listeria* was increased in treated mice. No effect on delayed hypersensitivity was noted (Boorman et al. 1982).

Oral exposure to indomethacin (2.5, 5, or 10 mg/kg/day for 3 days) decreased formation of PFC in C57BL/6 mice after immunization to sheep erythrocytes. Studies showed a dose-dependent decrease in the number of PFC/10^6 spleen cells; decrease ranged from 43% to 97%. Similar inhibition was observed at 5 mg/kg/day indomethacin and various concentrations of sheep erythrocytes (2.5×10^8 and 5×10^8); decreases ranged from 47% to 68%. Indomethacin also inhibited antibody response to *P. aeruginosa* LPS; total response was decreased by 44% (Rojo et al. 1981).

Oral administration of 6 mg/kg/day indomethacin for 4 days produced a 32% decrease in total number of lymphocytes in Swiss male mice. No effect was noted at earlier time points (i.e., 2 or 3 days). An increase in the number of colonies/10^5 bone marrow cells (2.7- to 3.9-fold) also was noted in mice that were administered indomethacin for 4 days. Indomethacin also decreased PGE2 (25-43%) and PGF2α (41-56%) levels in bone marrow cells after 4 days of administration (Fontagne et al. 1980).

Male CBA mice were intraperitoneally injected with 0.7, 4, or 8 mg/kg indomethacin. Two to 24 hours after exposure, mice were euthanized and spleens removed. A dose-dependent increase in splenocyte proliferation was noted after 2 hours, with a 14.3-fold increase in proliferation at the highest dose tested. A time-dependent increase in proliferation was also noted when mice were treated with 4 mg/kg indomethacin, with a maximal fold change of 31.4-fold at 24 hours. Distribution of T-cell phenotypes was not affected by indomethacin administration (Gonzalez-Cabello et al. 1987).

Kushima and colleagues (2007, 2009) evaluated effects of indomethacin in young Sprague-Dawley rats after *in utero* exposure. In 3-week old pups from dams treated with 0.25, 0.5, or 1.0 mg/kg indomethacin on GD 18-21, a significant increase (31%) in the number of spleen cells was observed in males from the highest dose group. Immunophenotyping of splenocytes showed a dose-dependent increase in the proportion of CD45RA+ cells in male pups. However, a similar

increase in peripheral blood lymphocytes was noted. No effect on serum IgM or IgG levels was reported in males or females. A significant decrease in anti-KLH IgG titers, but not IgM titers was reported in males from the highest dose group tested (Kushima et al. 2007). When doses of 0.5, 1.0, or 2.0 mg/kg indomethacin were used, a significant decrease in splenocyte IL-10 levels were reported in males; no effects on IL-6, IL-2, IL-4, TNF, or IFN-γ levels were noted in either sex (Kushima et al. 2009).

Indomethacin (1 or 2 mg/kg administered twice daily for 3 days to adjuvant induced arthritic Sprague-Dawley rats) reduced PHA-induced lymphocyte proliferation in a dose-dependent manner (data in graph). LPS-stimulated proliferation was also inhibited at both doses, however the response was partially recovered at the higher tested indomethacin dose (data in graph) (Seng et al. 1990).

Indomethacin increased the total number of cells, and number of T- and B-cells up to 14 days after birth, in newborn ddy mice intraperitoneally injected with 5 μg/g every 2 days from birth (data in graphs) (Shibuya et al. 1986).

In vitro data with cells or cell lines

Indomethacin (3 μM) inhibited proliferation of lymphocytes isolated from C57BL/6 mice (data in graph). Additionally, dose-dependent inhibition LPS-induced proliferation of isolated lymphocytes was noted (Barasoain et al. 1980).

Indomethacin dose-dependently increased male rat (strain not provided) ConA-induced lymphocyte proliferation after an 18-hour incubation (LOAEC = 1 μM). A time-course evaluation with 1 μM indomethacin showed that ConA-inducted lymphocyte proliferation was enhanced at incubation times up to 30 hours. Proliferation at exposure times ranging from 36 to 66 hours were not different from controls (Calder et al. 1991).

Indomethacin (50 nM to 50 μM) dose dependently increased LAK activity in BALB/c mouse splenocytes that were cocultured with recombinant IL-2. Increased lysis of JC tumor cells was observed, reaching a maximum response of 123.6 lytic units at 50 μM compared to 43.6 lytic units for IL-2 alone. Studies also showed that the increased response, compared to addition of IL-2 alone, was observed when culture conditions were maintained for up to 4 days. Addition of nylon wool to the culture, abrogated the induction of LAK response observed in the presence of indomethacin (Chao et al. 1989).

Increased time-dependent proliferation was observed in lymphocytes, from CBA mice, treated with 10 μg/mL indomethacin. After 6 and 24 hours, proliferation was increased 4.3- and 46.6-fold, respectively (Gonzalez-Cabello et al. 1987).

Indomethacin decreased IL-4 levels in ConA-stimulated splenocytes isolated from 3-week old male rats (LOAEL = 50 μM). No effect was noted in splenocytes from females. Decreased IL-6 splenocyte levels was observed in cells obtained from females and treated with 2.0 μM indomethacin. No effect on IL-2, IL-10, IFN-γ, and TNF-α were noted (data not shown or in graph) (Kushima et al. 2009).

Mode of action information

Indomethacin induced effects on prostaglandin synthesis was associated with several immune effects. Lala and Parhar (1988) suggested that indomethacin effects are associated with suppression of prostaglandin synthesis. Rojo and colleagues (1981) and Franceschi and colleagues (1988) proposed that indomethacin inhibition of prostaglandin synthesis leads to altered T-cell function, and NK- and antibody-dependent cytotoxicity.

Differential effects on T-cell and B-cell-induced lymphocyte proliferation were reported. A dose-dependent effect on T-cell function was reported, while an inverse effect on B-cell function was noted (Seng et al. 1990).

Indomethacin has been postulated to produce immune effects through inhibition of Th1, and to a lesser extent Th2, responses (Yamaki et al. 2003). Studies conducted by Jaramillo and colleagues (1992) supported this proposed mode of action.

References

Barasoain I, Rojo JM, Portolés A. 1980. “In vivo” effects of acetylsalicylic acid and two ether derived compounds on primary immune response and lymphoblastic transformation. Immunopharmacology 2: 293–300.

Boorman GA, Luster MI, Dean JH, Luebke RW. 1982. Effect of indomethacin on the bone marrow and immune system of the mouse. Journal of clinical & laboratory immunology 7: 119–26.

Calder PC, Bond JA, Newsholme EA. 1991. Effect of inhibitors of eicosanoid synthesis upon lymphocyte proliferation. Biochemical Society transactions 19: 88s.

Chao TY, Ohnishi H, Chu TM. 1989. Augmentation of murine lymphokine (rIL-2)-activated killer cell activity by indomethacin. Molecular biotherapy 1: 318–22.

Fontagne J, Adolphe M, Semichon M, Zizine L, Lechat P. 1980. Effect of in vitro treatment with indomethacin on mouse granulocyte-macrophage colony-forming cells in culture (CFUC). Possible role of prostaglandins. Experimental hematology 8: 1157–64.

Franceschi D, Warnaka P, Kim B. 1988. Indomethacin inhibition of IL-2-induced splenocyte proliferation. Current surgery 45: 474–6.

Gonzalez-Cabello R, Perl A, Kalmar L, Gergely P. 1987. Short-term stimulation of lymphocyte proliferation by indomethacin in vitro and in vivo. Acta physiologica Hungarica 70: 25–30.

Hartel C, von Puttkamer J, Gallner F, Strunk T, Schultz C. 2004. Dose-dependent immunomodulatory effects of acetylsalicylic acid and indomethacin in human whole blood: potential role of cyclooxygenase-2 inhibition. Scandinavian journal of immunology 60:412–20; doi:10.1111/j.0300-9475.2004.01481.x.

Jaramillo A, Bhattacherjee P, Sonnenfeld G, Paterson CA. 1992. Modulation of immune responses by cyclo-oxygenase inhibitors during intraocular inflammation. Curr Eye Res 11: 571–579.

Jawad AM, Rogers HJ. 1984. The effects of flurbiprofen and indomethacin on the mitogenic response of human peripheral mononuclear cells. Immunopharmacology 7: 59–67.

Kushima K, Oda K, Sakuma S, Furusawa S, Fujiwara M. 2007. Effect of prenatal administration of NSAIDs on the immune response in juvenile and adult rats. Toxicology 232:257–67; doi:10.1016/j.tox.2007.01.012.

Kushima K, Sakuma S, Furusawa S, Fujiwara M. 2009. Prenatal administration of indomethacin modulates Th2 cytokines in juvenile rats. Toxicology letters 185:32–7; doi:10.1016/j.toxlet.2008.11.013.

Lala PK, Parhar RS. 1988. Cure of B16F10 melanoma lung metastasis in mice by chronic indomethacin therapy combined with repeated rounds of interleukin 2: characteristics of killer cells generated in situ. Cancer research 48: 1072–9.

Rojo JM, Barasoain I, Portoles A. 1981. Further studies on the immunosuppressive effects of indomethacin. International journal of clinical pharmacology, therapy, and toxicology 19: 220–2.

Seng GF, Benensohn J, Bayer BM. 1990. Changes in T and B lymphocyte proliferative responses in adjuvant-arthritic rats: antagonism by indomethacin. European journal of pharmacology 178: 267–73.

Shibuya T, Izuchi K, Koga Y, Nomoto K, Shirakawa K. 1986. Prostaglandin modulation of the postnatal development of T and B lymphocytes in the spleens of newborn mice. Dev Comp Immunol 10: 419–428.

Tanaka K, Tanaka H, Kanemoto Y, Tsuboi I. 1998. The effects of nonsteroidal anti-inflammatory drugs on immune functions of human peripheral blood mononuclear cells. Immunopharmacology 40: 209–17.

Yamaki K, Uchida H, Harada Y, Yanagisawa R, Takano H, Hayashi H, et al. 2003. Effect of the nonsteroidal anti-inflammatory drug indomethacin on Th1 and Th2 immune responses in mice. Journal of pharmaceutical sciences 92:1723–9; doi:10.1002/jps.10380.

Isonicotinic Acid Hydrazide (IAH) [CASRN 54-85-3]

Human Data

Data from epidemiology studies

In 19 cases of INH-induced liver failure, antibodies were present in sera of 15 patients. Anti-INH antibodies were present in 8 patients. Additionally, anti-cytochrome P450 antibodies were identified in up to 14 patients. Antibodies were not detected in patients that were treated with INH but did not have significant liver injury (Metushi et al. 2014c). In eight INH-induced liver failure patients, the dominant serum immunoglobulin isotype of anti-INH antibodies was IgG. A low titer of IgM was observed in two patients, while IgA and IgE antibodies were not detected. Phenotyping the IgG antibody indicated that the isotype was IgG3 (Metushi et al. 2014b).

INH (1.25 μg/mL) did not stimulate PGE3 production in polymorphonuclear leukocytes or modulate PHA-stimulated mononuclear leukocytes transformations. No effect on PG2 production was observed at 5 μg/mL (Zeis 1987).

In vitro data with cells or cell lines

In a series of studies, Kucharz and colleagues studies the immunomodulatory effects of IAH. In 5 μg/mL PHA-stimulated T-cells, IAH increased cellular proliferation (16% to 27%) at concentrations ranging from 0.01 to 0.0001 mM. (Kucharz and Sierakowski 1990a). In PBMC stimulated with 5 ng/mL anti-CD3 antibody, IAH produced a biphasic response. At 1 and 10 mM IAH decreased (53.6% and 24.4%, respectively) cell proliferation. Increased cellular proliferation (18-47%) was observed at concentrations ranging from 0.0001 to 0.1 mM. A similar biphasic pattern was observed when 10 ng/mL anti-CD3 antibody was used. In T-cells stimulated with anti-CD3 antibody, PHA, or PHA with PMA, IAH also modulated proliferation in a biphasic manner (Kucharz and Sierakowski 1990a). In cells stimulated with 5 μg/mL PHA and 20 ng/mL PMA, 0.1 to 10 mM IAH decreased T-cell proliferation 17% to 46%. At 0.001 mM IAH, at significant increase (21%) in T-cell proliferation was observed (Kucharz and Sierakowski 1990d). In cells stimulated with IL-2, IAH decreased cell proliferation 0.1 and 1 mM (71% and 47%, respectively) and increased proliferation at 0.01 to 0.001 mM (8% to 12%, respectively (Kucharz 1995).

IAH also decreased T-cell IL-2 production at 0.1 and 1 mM (44.7% and 71.6%, respectively) and increased T-cell IL-2 production at 0.01 to 0.0001 mM (105% to 115%). No effect on IL-2 receptor expression in T-cells was observed (Kucharz and Sierakowski 1990b).

IAH decreased IL-1 production from human monocytes in a dose-dependent manner in the absence or presence of lipopolysaccharide (LOAEL = 0.001 mM) (Kucharz and Sierakowski 1992).

In the absence of PHA, IAH stimulated proliferation of Jurkat cells (LOAEL = 0.01 mM). In the presence of PHA (2 or 5 μg/mL), IAH stimulation was observed at higher concentrations (1 and 10 mM) while at lower concentrations no effect was observed (Kucharz and Sierakowski 1990c).

When PMA (20 ng/mL) or PMA (20 ng/mL) and PHA (5 µg/mL) were added to the media, increased Jurkat cellular proliferation was observed at 0.001 mM (32%) and 0.01 and 0.001 mM (8% and 18%, respectively) (Kucharz and Sierakowski 1990d).

INH (5 µg/mL) did not have any effect on the phagocytic activity or intracellular killing activity on polymorphonuclear leukocytes obtained from healthy volunteers (Okuyan et al. 2005).

Mode of action information

Metushi and colleagues proposed that INH produced an immune response that leads to liver injury (Metushi et al. 2014c, 2014b).

Rodent Data

Data from in vivo immunotoxicology or toxicology studies

Female *Nat1/2(-/-)* mice were treated with INH either by oral gavage (100 mg/kg/day) for up to 7 days or by feed (0.2%) for 35 days. In mice treated by gavage, significant decrease in M1 macrophages and increase in M2a and M2b macrophages in cervical lymph nodes was noted. No effect on the M2c macrophages was observed. Comparatively, no effect was noted in the macrophage phenotypes obtained from mice that were exposed by feed (Metushi et al. 2014a).

INH (0.1 to 1.0 mg/10 µL) did not alter the weight of popliteal lymph nodes from C57BL/10 mice 7 days after subcutaneous injection (Kammuller et al. 1989). A lack of effect on popliteal lymph nodes from Brown Norway rats also was observed when exposed to 5 mg/50 µL INH (Verdier et al. 1990).

Four female Cbl-b-/-, C57BL/6 background that lack an E3 ubiquitin ligase, were provided diets containing 0.2% w/w INH for 5 weeks. Blood was collected to assess serum cytokine levels. Significant decreases in serum IL-12 and IL-1α was noted in female Cbl-b-/- mice (data provided in graph). No effects on IL-1β, IL-2, IL-3, IL-4, IL-5, IL-6, IL-9, IL-10, IL-12 (p40), IL-13, IL-17A, eotaxin, GCSF, GMCSF, IFNγ, KC, MCP-1, MIP-1α, MIP-1β, RANTES, and TNF-α were observed (data provided in supplementary materials) (Metushi and Uetrecht 2014).

In vitro data with cells or cell lines

In HT-2 cells, stimulated with IL-2 (3 or 30 U/mL), increased proliferation was observed at 1 and 10 mM IAH at 30 U/mL and only at 1 mM at 3 U/mL. No effect on proliferation was observed in cells stimulated with 60 U/mL IL-2 (Kucharz and Sierakowski 1990c). Additionally, no effect on proliferation by IAH was observed in HT-2 cells stimulated with PMA (data not provided) (Kucharz and Sierakowski 1990d).

Mode of action information

No data were located.

References

Kammuller ME, Thomas C, De Bakker JM, Bloksma N, Seinen W. 1989. The popliteal lymph node assay in mice to screen for the immune disregulating potential of chemicals--a preliminary study. International journal of immunopharmacology 11: 293–300.

Kucharz EJ. 1995. Studies on immunomodulatory properties of isoniazid. Influence of isoniazid on responsiveness of peripheral blood mononuclear cells to interleukin-2. Central European journal of public health 3: 65–6.

Kucharz EJ, Sierakowski SJ. 1990a. Studies on immunomodulatory properties of isoniazid. I. Effect of isoniazid on mitogen- and anti-CD3 antibody-induced proliferation of human peripheral blood mononuclear cells and T cells. Journal of hygiene, epidemiology, microbiology, and immunology 34: 99–105.

Kucharz EJ, Sierakowski SJ. 1990b. Studies on immunomodulatory properties of isoniazid. II. Effect of isoniazid on interleukin 2 production and interleukin 2-receptor expression. Journal of hygiene, epidemiology, microbiology, and immunology 34: 207–11.

Kucharz EJ, Sierakowski SJ. 1990c. Studies on immunomodulatory properties of isoniazid. III. Effect of isoniazid on proliferation of interleukin-dependent and interleukin-independent cell line. Journal of hygiene, epidemiology, microbiology, and immunology 34: 305–8.

Kucharz EJ, Sierakowski SJ. 1990d. Studies on immunomodulatory properties of isoniazid. IV. Effect of isoniazid on T lymphocyte activation by phorbol ester tumor promoter. Journal of hygiene, epidemiology, microbiology, and immunology 34: 381–5.

Kucharz EJ, Sierakowski SJ. 1992. Studies on immunomodulatory properties of isoniazid: V. Influence of isoniazid on secretion of interleukin-1. Journal of hygiene, epidemiology, microbiology, and immunology 36: 119–22.

Metushi IG, Cai P, Vega L, Grant DM, Uetrecht J. 2014a. Paradoxical attenuation of autoimmune hepatitis by oral isoniazid in wild-type and N-acetyltransferase-deficient mice. Drug metabolism and disposition: the biological fate of chemicals 42:963–73; doi:10.1124/dmd.113.056622.

Metushi IG, Lee WM, Uetrecht J. 2014b. IgG3 is the dominant subtype of anti-isoniazid antibodies in patients with isoniazid-induced liver failure. Chemical research in toxicology 27:738–40; doi:10.1021/tx500108u.

Metushi IG, Sanders C, Lee WM, Uetrecht J. 2014c. Detection of anti-isoniazid and anti-cytochrome P450 antibodies in patients with isoniazid-induced liver failure. Hepatology (Baltimore, Md) 59:1084–93; doi:10.1002/hep.26564.

Metushi IG, Uetrecht J. 2014. Isoniazid-induced liver injury and immune response in mice. Journal of immunotoxicology 11:383–92; doi:10.3109/1547691x.2013.860644.

Okuyan B, Izzettin FV, Sancar M, Ertas O, Cevikbas A, Gurer US. 2005. Effect of antituberculous drugs on human polymorphonuclear leukocyte functions in vitro. International immunopharmacology 5:1337–42; doi:10.1016/j.intimp.2005.03.002.

Verdier F, Virat M, Descotes J. 1990. Applicability of the popliteal lymph node assay in the Brown-Norway rat. Immunopharmacology and immunotoxicology 12:669–77; doi:10.3109/08923979009019683.

Zeis BM. 1987. Effects of anti-tuberculosis drugs on the production of prostaglandin E2 and on mononuclear leucocyte transformation. Chemotherapy 33:204–10; doi:10.1159/000238496.

Lead (II) Acetate Trihydrate [CASRN 6080-56-4]

Human Data

Data from epidemiology studies

No data were located.

In vitro data with cells or cell lines

Lead (lead acetate 5.0 mg - 1.5 ng/mL, or lead chloride 0.5 mg - 0.15 ng/mL for 24 hours) significantly reduced cell vitality and/or proliferation and affected secretion of proinflammatory, TH1 and TH2 cytokines in human peripheral mononuclear blood cells that were stimulated with either heat-killed *Salmonella enteritidis* (hk-SE) or monoclonal antibodies. At lower lead levels, expression of IFN-γ, IL-1β and TNF-α were reduced. Monoclonal antibody induced IL-4, IL-6 and IL-10 and hk-SE induced IL-10 and IL-6 levels were increased in the presence of lower lead levels. The authors suggest that lower dose lead suppresses the TH1 cytokine and the proinflammatory cytokines while the increased IL-4 and/or IL-10 production can induce and maintain a TH2 immune response (Hemdan et al. 2005).

Thirty male lead-exposed (battery recycling industry) workers with a blood lead level > 10 µg/dL and 27 unexposed healthy volunteers without any history of occupational exposure to lead were selected for this study. The serum level of IgA was found to be significantly increased in the lead-exposed group as compared to controls. No differences were observed in serum IgG and IgM levels. Both the level of nitric oxide production after stimulation with zymosan-A and the neutrophil respiratory burst as measured by nitroblue tetrazolium reduction were comparable in neutrophils from lead-exposed and unexposed volunteers (Mishra et al. 2006).

Mode of action information

Lead acetate (1 µM) induced activation of NF-κB in primary human CD4+ T lymphocytes. This lead induced activation was blocked by antibodies for p65 and p50 subunits (indicating that the p65:p50 heterodimer (NF-κB) is involved), but not by cRel. Lead acetate (100 pM – 100 µM) did not activate NF-κB in 4 different T cell lines, suggesting that these cell lines may not be a reliable system for studying transcriptional activation in human T cells (Pyatt et al. 1996).

Rodent Data

Data from in vivo immunotoxicology or toxicology studies

Lead acetate suppressed macrophage-dependent immune responses in cells taken from female BDF1 mice exposed to lead in drinking water at concentrations from 0 to 1000 ppm for 3 weeks. The T-cell dependent sheep erythrocyte primary immune response was suppressed by approximately 40-50% in all lead-exposed groups. In contrast, the *E. coli* lipopolysaccharide (T-cell and macrophage-independent) induced response, was not suppressed (Blakley and Archer 1981). Lead did not alter the ability of T-cell mitogens to induce interferon (Blakley et al. 1982).

Lead acetate effects were modulated by maternal protein intake. Fischer 344 rats were exposed to lead acetate (250 ppm) in the drinking water during breeding and pregnancy until parturition and were fed isocaloric diets (either 20% casein or 10% casein). Offspring exposed to lead and high maternal dietary protein had significantly elevated levels of both IL-4 and TNF-α (values not provided). Offspring exposed to lead and low maternal dietary protein had significantly reduced IL-4 levels compared to the lead control group (values not provided). No other changes were observed, and immune parameters measured in the dams were not affected by treatment (Chen et al. 2004).

In a study comparing immunotoxic effects of various lead salts, Balb/c mice were treated for five consecutive days between immunization and elicitation with intraperitoneal injections of 0.5 or 6 mg/kg of a lead salt. A statistically significant increase in delayed hypersensitivity (as measured by footpad swelling) was observed following administration of lead acetate (55.0% increase in footpad thickness as compared to controls; LOAEL = 6 mg/kg) (Descotes et al. 1984).

Exposure to lead acetate resulted in a decreased ability of mice to survive a sublethal dose of a virulent strain of *S. typhimurium*. C3H/HeN mice were exposed to lead acetate (5 or 10 mM) in the drinking water for up to 18 weeks. At week 16, mice were infected with *S. typhimurium*. 40% of the mice exposed to 5 mM lead acetate survived the infection with a median survival of 26 days. None of the mice treated with 10 mM lead acetate survived, with death occurring within three weeks of becoming infected. In contrast, 80% of control mice survived with a median survival of 60 days. The ability of splenocytes, cultured from the lead-treated and control mice showed a marked reduction in the production of IFN-γ (27% and 35% in mice treated with 5 and 10 mM lead acetate, respectively) and IL-12p40 (42-45% in mice treated with 5 and 10 mM lead acetate, respectively, as compared to induced control). Secretion of IL-4 by splenocytes from lead-treated mice was 3 to 3.6-fold higher than in control mice (Fernandez-Cabezudo et al. 2007).

Adult Sprague-Dawley females were treated with 500 ppm lead acetate via drinking water either early in gestation (days 3-9) or late in gestation (days 15-21). Offsping were assessed as adults. Significantly depressed DTH responses as well as increased IL-10 production, relative monocyte numbers and relative thymic weights were reported in female offspring exposed to lead during late gestation. Male offspring exposed during late gestation had significantly increased IL-12 production and decreased IL-10 production while the DTH response, relative monocyte numbers and thymic weights were unchanged compared to controls. The authors found that adherent splenocytes (likely macrophages) and T lymphocytes are the primary immune cells affected during fetal lead exposure and that gender may influence immunotoxicity due to lead exposure (Bunn et al. 2001).

Lead acetate increased IL-4 production in mice at 40 and 400 mg/L and decreased IFN-γ levels in mice at 400 mg/L. Adult Swiss mice were administered lead acetate in drinking water for 14 days. The authors concluded that low level lead exposure enhances a Th2 response while high lead levels can either stimulate Th2 immune activity or reduce Th1 activity, thus resulting in an imbalance between Th1 and Th2 activation (Iavicoli et al. 2004).

Lead acetate (100 or 1000 ppm in drinking water) did not alter the ability of splenocytes isolated from exposed male Alderly Park rats to mediate native and interferon activated natural cytotoxicity at 2,4,6 and 8 weeks following commencement of exposure. Splenic T-cell function of treated rats as determined by phytohaemagglutinin induced proliferation was comparable to control values (Kimber et al. 1986).

Lead acetate (10 mM in the drinking water for 8 weeks) did not suppress the primary direct humoral immune response to T-dependent antigen (sheep erythrocyte) and T-independent antigens (TNP-LPS, TNP-Ficoll) in several inbred (A, BALB/c, C57Bl/6, DBA/1, SJL, and NZW/NZB F1) and an outbred (CFW) strains of mice (Mudzinski et al. 1986).

Lead acetate (200 ppm either in the drinking water or given intraperitoneally for 4 weeks) decreased the number of lymphocyte cells and cellularity (i.e., number of cells per mg tissue) in the thymus, but no significant changes in either parameter were reported for the submaxillary lymph nodes. Proliferation of T cells stimulated by ConA and proliferation of B cells stimulated by LPS was increased by lead in the thymus by both routes of exposure. In the submaxillary lymph nodes, there was a decrease in the proliferation of T cells following treatment by either route (Teijon et al. 2010).

In vitro data with cells or cell lines

RAW 264.7 cells were treated with 100 ppm lead acetate for 24 hours in the presence or absence of LPS. Lead produced a statistically significant inhibition of the level of LPS-induced nitric oxide (data not provided). No effect on cytotoxicity was observed (Mishra et al. 2006).

Mode of action information

C3H/HeN mice were exposed to lead acetate (0, 5 or 10 mM in drinking water for periods of up to 18 weeks) and inoculated with a virulent strain of *S. typhimurium*. Sera were collected on days 15 and 38 post infection. The authors report that the IgG2a antibodies were elevated in control mice by day 38 post infection (0.09 ± 0.05 on day 15 vs. 0.30 ± 0.03 on day 38; an increase of 300% from day 15), but were only slightly increased in lead-exposed mice (0.11 ± 0.01 on day 15 vs. 0.16 ± 0.02 on day 38). IgG1 isotype antibodies (an isotype induced by IL-4) were significantly elevated in lead exposed mice on day 38, as compared to control mice. The authors conclude that lead acetate induces a subtle but substantial shift toward a Th2-type immune response to infection with Salmonella organism (Fernandez-Cabezudo et al. 2007).

A single intraperitoneal exposure to lead acetate (12 mg/kg) in B6C3F1 mice produced changes in cell surface markers on discrete subpopulations of lymphoid cells from the spleen and bone marrow. The authors concluded that while the changes may not correlate with functional activity of the cells, they seemed to predict a shift to immature cell types, which correlated with the increase in progenitor cells observed (Burchiel et al. 1987).

References

Blakley BR, Archer DL. 1981. The effect of lead acetate on the immune response in mice. Toxicology and applied pharmacology 61: 18–26.

Blakley BR, Archer DL, Osborne L. 1982. The effect of lead on immune and viral interferon production. Canadian journal of comparative medicine : Revue canadienne de medecine comparee 46: 43–6.

Bunn TL, Parsons PJ, Kao E, Dietert RR. 2001. Exposure to lead during critical windows of embryonic development: differential immunotoxic outcome based on stage of exposure and gender. Toxicological sciences : an official journal of the Society of Toxicology 64: 57–66.

Burchiel SW, Hadley WM, Cameron CL, Fincher RH, Lim TW, Elias L, et al. 1987. Analysis of heavy metal immunotoxicity by multiparameter flow cytometry: correlation of flow cytometry and immune function data in B6CF1 mice. International journal of immunopharmacology 9: 597–610.

Chen S, Golemboski K, Piepenbrink M, Dietert R. 2004. Developmental immunotoxicity of lead in the rat: influence of maternal diet. Journal of toxicology and environmental health Part A 67:495–511; doi:10.1080/15287390490276520.

Descotes J, Evreux JC, Laschi-Locquerie A, Tachon P. 1984. Comparative effects of various lead salts on delayed hypersensitivity in mice. Journal of applied toxicology : JAT 4: 265–6.

Fernandez-Cabezudo MJ, Ali SA, Ullah A, Hasan MY, Kosanovic M, Fahim MA, et al. 2007. Pronounced susceptibility to infection by Salmonella enterica serovar Typhimurium in mice chronically exposed to lead correlates with a shift to Th2-type immune responses. Toxicology and applied pharmacology 218:215–26; doi:10.1016/j.taap.2006.11.018.

Hemdan NY, Emmrich F, Adham K, Wichmann G, Lehmann I, El-Massry A, et al. 2005. Dose-dependent modulation of the in vitro cytokine production of human immune competent cells by lead salts. Toxicological sciences : an official journal of the Society of Toxicology 86:75–83; doi:10.1093/toxsci/kfi177.

Iavicoli I, Marinaccio A, Castellino N, Carelli G. 2004. Altered cytokine production in mice exposed to lead acetate. International journal of immunopathology and pharmacology 17:97–102; doi:10.1177/03946320040170s216.

Kimber I, Jackson JA, Stonard MD. 1986. Failure of inorganic lead exposure to impair natural killer (NK) cell and T lymphocyte function in rats. Toxicology letters 31: 211–8.

Mishra KP, Chauhan UK, Naik S. 2006. Effect of lead exposure on serum immunoglobulins and reactive nitrogen and oxygen intermediate. Human & experimental toxicology 25:661–5; doi:10.1177/0960327106070453.

Mudzinski SP, Rudofsky UH, Mitchell DG, Lawrence DA. 1986. Analysis of lead effects on in vivo antibody-mediated immunity in several mouse strains. Toxicology and applied pharmacology 83: 321–30.

Pyatt DW, Zheng JH, Stillman WS, Irons RD. 1996. Inorganic lead activates NF-kappa B in primary human CD4+ T lymphocytes. Biochemical and biophysical research communications 227:380–5; doi:10.1006/bbrc.1996.1516.

Teijon C, Blanco MD, Romero CS, Beneit JV, Villarino AL, Guerrero S, et al. 2010. Study of response of thymic and submaxillary lymph node lymphocytes to administration of lead by different routes. Biological trace element research 135:74–85; doi:10.1007/s12011-009-8495-6.

Mannitol [CASRN 69-65-8]

Human Data

Data from epidemiology studies

No data were located.

In vitro data with cells or cell lines

Increased urinary excretion of 9α,11β-prostaglandin F2 and leukotriene-4 were reported in association with mannitol-induced bronchoconstriction in 14 asthmatic patients. Urinary excretion of 9α,11β-prostaglandin F2 and leukotriene-4 increased from 61 to 92 and 19 to 31 ng × mmol/creatinine, respectively (Brannan et al. 2006). A separate study reported that repeated challenge with mannitol induced refractoriness in asthma patients. The mannitol refractoriness was associated with maintained release of 9α,11β-prostaglandin F2 and leukotriene-4 (Larsson et al. 2011).

Increased proportion of submucosal MC_{TC} was observed in asthmatic individuals with airway hyperresponsiveness to mannitol compared to asthmatic individuals without responses to mannitol. The percentage MC_{TC} increased from 18.7% to 40.3%, but the increase in the numbers of MC_{TC} between the two groups was not significantly increased. Increased gene expression of thymic stromal lymphopoietin and carboxypeptidase AM also were reported (Sverrild et al. 2016).

Mannitol significantly increased 9α,11β-prostaglandin F2, leukotriene-C4, and histamine release from cord blood-derived mast cells (LOAEL = 0.7 M for all endpoints). At the same tested mannitol concentrations (0.3-1.0 M), no concordant increase in lactate dehydrogenase release was observed suggesting cell viability was not affected. The ratio of 9α,11β-prostaglandin F2 to leukotriene-C4 was 156-1 (Gulliksson et al. 2006).

Mannitol did not induce DNA damage in human leukocytes at concentrations from 1.25 to 10 mM (Frenzilli et al. 2000).

Mannitol (22 mmol/L) did not increase IL-6 or TNF-α secretion from monocytes treated with glucose (11 mmol/glucose) for 24 hours. A similar lack of effect was observed when cells were incubated for 48 hours (Morohoshi et al. 1996).

At the highest concentration tested (100,000 µM), mannitol did not reduce cell viability in human LCLs or PBMCs. Mannitol (50,000 µM) did not modulate TNF-α, IL-6, IL-2, IL-4, IL-10, or IFNγ release in LCLs pre-treated with phorbol 12-myristate 13-acetate/ionomycin stimulated cells (Markovic et al. 2015).

Mannitol did not inhibit growth of human granulocyte precursor cells at a concentration up to 5 mM (Holdener et al. 1983).

Mode of action information

Mannitol is shown to narrow the airway in asthmatic, but not healthy, test subjects (Brannan et al. 2001, 2003, 2000). Mannitol is proposed to increase osmolarity of airway surface liquid, leading to an increase in mediator release (e.g., histamine, prostaglandins, and leukotrienes) from inflammatory cells which induces bronchoconstriction (Brannan et al. 2006; Sverrild et al. 2016). One mediator that is proposed to be released is prostaglandin D2 from mast cells (Brannan et al. 2003, 2006).

Rodent Data

Data from in vivo immunotoxicology or toxicology studies

No data were located.

In vitro data with cells or cell lines

No data were located.

Mode of action information

No data were located.

References

Brannan JD, Anderson SD, Freed R, Leuppi JD, Koskela H, Chan H-K. 2000. Nedocromil Sodium Inhibits Responsiveness to Inhaled Mannitol in Asthmatic Subjects. American Journal of Respiratory and Critical Care Medicine 161:2096–2099; doi:10.1164/ajrccm.161.6.9908096.

Brannan JD, Anderson SD, Gomes K, King GG, Chan HK, Seale JP. 2001. Fexofenadine decreases sensitivity to and montelukast improves recovery from inhaled mannitol. Am J Respir Crit Care Med 163:1420–1425; doi:10.1164/ajrccm.163.6.2006019.

Brannan JD, Gulliksson M, Anderson SD, Chew N, Kumlin M. 2003. Evidence of mast cell activation and leukotriene release after mannitol inhalation. Eur Respir J 22: 491–496.

Brannan JD, Gulliksson M, Anderson SD, Chew N, Seale JP, Kumlin M. 2006. Inhibition of mast cell PGD2 release protects against mannitol-induced airway narrowing. The European respiratory journal 27:944–50; doi:10.1183/09031936.06.00078205.

Frenzilli G, Bosco E, Barale R. 2000. Validation of single cell gel assay in human leukocytes with 18 reference compounds. Mutation research 468: 93–108.

Gulliksson M, Palmberg L, Nilsson G, Ahlstedt S, Kumlin M. 2006. Release of prostaglandin D2 and leukotriene C4 in response to hyperosmolar stimulation of mast cells. Allergy 61:1473–9; doi:10.1111/j.1398-9995.2006.01213.x.

Holdener EE, Park CH, Belt RJ, Stephens RL, Hoogstraten B. 1983. Effect of mannitol and plasma on the cytotoxicity of cisplatin. European journal of cancer & clinical oncology 19: 515–8.

Larsson J, Perry CP, Anderson SD, Brannan JD, Dahlen SE, Dahlen B. 2011. The occurrence of refractoriness and mast cell mediator release following mannitol-induced bronchoconstriction.

Journal of applied physiology (Bethesda, Md : 1985) 110:1029–35; doi:10.1152/japplphysiol.00978.2010.

Markovic T, Gobec M, Gurwitz D, Mlinaric-Rascan I. 2015. Characterization of human lymphoblastoid cell lines as a novel in vitro test system to predict the immunotoxicity of xenobiotics. Toxicology letters 233:8–15; doi:10.1016/j.toxlet.2014.12.013.

Morohoshi M, Fujisawa K, Uchimura I, Numano F. 1996. Glucose-dependent interleukin 6 and tumor necrosis factor production by human peripheral blood monocytes in vitro. Diabetes 45: 954–9.

Sverrild A, Bergqvist A, Baines KJ, Porsbjerg C, Andersson CK, Thomsen SF, et al. 2016. Airway responsiveness to mannitol in asthma is associated with chymase-positive mast cells and eosinophilic airway inflammation. Clinical and experimental allergy : journal of the British Society for Allergy and Clinical Immunology 46:288–97; doi:10.1111/cea.12609.

Nickel (II) Sulfate Hexahydrate (NiSO4) [CASRN 10101-97-0]

Human Data

Data from epidemiology studies

No data were located.

In vitro data with cells or cell lines

In PBMC from five nickel allergic individuals 0.1 mM NiSO4 increased IL-4 and IFN-γ production. The peak effect was lower than when PBMC were incubated with PHA (data shown in graph) (Thomas et al. 2003).

NiSO4 (85 μg/mL) significantly upregulated expression of CD40, CD83, CD86, and CD54 markers on THP-1 cells. NiSO4 also significantly increased production of TNF-α and IL-8 in a dose-dependent manner. IL-6 production was significantly increased after exposure to 170 μg/mL (Miyazawa et al. 2007). Ade and colleagues noted that NiSO4 induced CD83, CD86, HLA-DR, and CD40 in a dose dependent manner in dendritic cells (Ade et al. 2007).

Mode of action information

NiSO4 was shown to alter dendritic cell phenotypes by activation of MAPKs and NF-□B. Additionally, NiSO4 induced IL-8, IL-6, and IL-12 p40 production (Ade et al. 2007; Antonios et al. 2009). Activation of the MAPK pathway may lead to upregulation of the Cys-Cys chemokine receptor, CCR7, which allows dendritic cells to migrate to the draining lymph nodes (Boisleve et al. 2004).

NiSO4 has a similar capacity to stimulate polyclonal CD+4 in Ni-allergic and -nonallergic individuals. Differences in clonal expansion or presence of Ni-binding motifs in MHC class II complexes could be involved in the development of allergic contact dermatitis (Lisby et al. 1999).

Rodent Data

Data from in vivo immunotoxicology or toxicology studies

Male Wistar rats were intratracheally instilled with 1, 2, 4, or 8 μmole NiSO4 per rat. The rats were euthanized up to 7 days after treatment. Treatment decreased the percentage of lymphocytes in pulmonary lymphoid cells (~55% to ~40%). NK activity in lymphoid lung cells was dependent on concentration and effector:target cell ratio. NK activity was decreased 1 day after treatment of 4 and 8 μmole NiSO4 at the effector to target cell ratio of 6:1. Two days after treatment suppression of NK activity was significant at doses ≥2 μmole NiSO4 and at the effector to target cell ratio of 6:1. After 7 days, a significant decrease was only observed at 8 μmole.
NiSO4 did not significantly modulate alveolar macrophage cytotoxic activity towards 3T12 target cells. Decreased levels of TNF-α was reported at all time points, while increased IFN-γ level was only noted after exposure to 8 μmole/rat on day 2 (data in graph) (Goutet et al. 2000).

Female B6C3F1 mice were exposed to NiSO4 aerosol for 6 hours per day, 5 days per week for 65 days. The actual exposure concentrations tested were 0.027, 0.11, or 0.45 mg Ni/m3. No change in thymic weight was reported. A significant increase in the number of nucleated cell numbers from lung-associated lymph nodes (LALN) and lavage fluid, after mice were immunized with sheep red blood cells, was noted at the highest dose tested (1.72- and 3.86-fold, respectively). Nonsignificant increase in the total antibody-forming cells (AFC)/(LALN) and nonsignificant decrease in AFC/spleen, after immunization with sheep red blood cells, were also noted after NiSO4 exposure. NiSO4 had no effect on mixed lymphocyte response of spleen cells after exposure to mitomycin C-treated spleen cells from DBA/2 mice. No effect in mitogen-stimulation assays also were noted by NiSO4 exposure. NiSO4 modulated pulmonary alveolar macrophage function, as measured by phagocytosis of opsonized erythrocytes; activity was significantly increased at 0.11 mg Ni/m3 (data not provided). Comparatively, NiSO4 had no effect on peritoneal macrophage phagocytosis activity at any tested dose. The highest dose of NiSO4 was associated with a significant two-fold increase in the number of B16F10 tumor nodules in the lungs of treated animals. However, incorporation of radiolabeled uridine was not considered biologically significant. NiSO4 did not affect splenic NK cell cytolytic activity (Haley et al. 1990).

Histopathological lesions in lungs, liver, thymus, kidneys, spleen, and lymph nodes were noted in male F344 rats intramuscularly injected with 125 µmole/kg NiSO4 over 26 days. Thymus glands from rats treated with the highest dose were much smaller than controls. Corticomedullary junction was not distinct and extensive degeneration and depletion of lymphocytes in the thymic cortex were noted. Additional tissues from these rats were evaluated further. In the lungs, large alveolar macrophages and polymorphonuclear leukocytes were noted in alveolar spaces and exudate. In the spleen and lymph nodes, lymphocytes were focally depleted in the white and red pulp (Knight et al. 1991).

Male Sprague-Dawley rats were exposed to 0.02, 0.05, and 0.1% NiSO4 in drinking water for 13 weeks. Effects on splenic lymphocyte and thymocyte subpopulations were evaluated. In splenic lymphocytes, increases in the total number of T-cells (LOAEL = 0.05%) and CD8+ T-cells (LOAEL = 0.02%) were reported. For CD4+ T-cells, the number of cells increased at 0.05% NiSO4 and then decreased at 0.1% dose. An increase in the total number of B cells was noted at 0.05% NiSO4. Subchronic exposure to 0.02% NiSO4 also increased the percentage and absolute number of thymocyte CD8+ cells. Exposure to 0.05% NiSO4 increased the total number of thymocyte cells, the percentage and absolute number of CD8+ cells, and absolute numbers of both CD4+ and B-cell populations. Exposure to 0.1% NiSO4 decreased the total number of thymocytes, the percentage and absolute number of CD4+ T cells, and absolute numbers of CD8+ T cells and of B cells (Obone et al. 1999).

Male C3H/He mice were provided 0.01, 0.05, 0.1, 0.25, 0.5, or 1% NiSO4 for 7 or 10 weeks. Mice were then sensitized with NiSO4 for 7 days and the footpad thickness was measured. The mice were then challenged with 0.4% NiSO4 and footpad swelling was measured 24 hours later. After 7 weeks of oral exposure, footpad swelling was not reduced at any of the tested doses.

However, after 10 weeks of exposure swelling was decreased (LOAEL = 0.1%) (Ishii et al. 1993).

Lymph nodes from C3H/He mice sensitized to NiSO4 were incubated with various monoclonal antibodies and then injected into naïve mice. After challenging with NiSO4, footpad swelling was measured. Cells treated with CD4-, Thy1.2-, or Ig-specific antibodies showed reduced swelling while cells treated with CD8 antibodies induced footpad swelling (Ishii et al. 1993).

Macrophage and PMN chemotactic activities in bronchoalveolar fluid were increased at 2 days after intratracheal instillation of 50 µg Ni per male Wistar rat. Activity then decreased until end of the experiment (14 days). Comparatively, LTB4 were maximally decreased at day 1 and then increased to control levels by day 14 (Hirano et al. 1994).

In vitro data with cells or cell lines

Spleen cells from C57BL/6 and Rag-1 deficient mice were stimulated with varying concentrations of NiSO4 (concentrations not provided). Using the ELISPOT assay, IL-2, IL-4 and IFN-γ secreting cells were identified in splenic cells from C57BL/6 mice. The number of IFN-γ cells were greater than the IL-2 and IL-4 cells. At higher concentrations (≥400 µM), the numbers of IL-2 and IL-2 secreting cells decreased while those secreting IFN-γ remained high. The number of IFN-γ cells did not increase due to previous immunization of NiSO4. In splenic cells from Rag-1 deficient mice, NiSO4 also contained IFN-γ secreting cells. However, at higher concentrations the cell levels decreased (in comparison to wild-type). Addition of NK1.1 antibodies produced a partial depletion in the cells. Further studies showed that addition of NKG2D antibodies reduced the number of IFN-γ secreting cells in wild-type and RAG-1 deficient mice (Kim et al. 2009).

Mode of action information

No data were located.

References

Ade N, Antonios D, Kerdine-Romer S, Boisleve F, Rousset F, Pallardy M. 2007. NF-kappaB plays a major role in the maturation of human dendritic cells induced by NiSO(4) but not by DNCB. Toxicological sciences : an official journal of the Society of Toxicology 99:488–501; doi:10.1093/toxsci/kfm178.

Antonios D, Ade N, Kerdine-Romer S, Assaf-Vandecasteele H, Larange A, Azouri H, et al. 2009. Metallic haptens induce differential phenotype of human dendritic cells through activation of mitogen-activated protein kinase and NF-kappaB pathways. Toxicology in vitro : an international journal published in association with BIBRA 23:227–34; doi:10.1016/j.tiv.2008.11.009.

Boisleve F, Kerdine-Romer S, Rougier-Larzat N, Pallardy M. 2004. Nickel and DNCB induce CCR7 expression on human dendritic cells through different signalling pathways: role of TNF-alpha and MAPK. The Journal of investigative dermatology 123:494–502; doi:10.1111/j.0022-202X.2004.23229.x.

Goutet M, Ban M, Binet S. 2000. Effects of nickel sulfate on pulmonary natural immunity in Wistar rats. Toxicology 145: 15–26.

Haley PJ, Shopp GM, Benson JM, Cheng YS, Bice DE, Luster MI, et al. 1990. The immunotoxicity of three nickel compounds following 13-week inhalation exposure in the mouse. Fundamental and applied toxicology : official journal of the Society of Toxicology 15: 476–87.

Hirano S, Asami T, Kodama N, Suzuki KT. 1994. Correlation between inflammatory cellular responses and chemotactic activity in bronchoalveolar lavage fluid following intratracheal instillation of nickel sulfate in rats. Archives of toxicology 68: 444–9.

Ishii N, Moriguchi N, Nakajima H, Tanaka S, Amemiya F. 1993. Nickel sulfate-specific suppressor T cells induced by nickel sulfate in drinking water. Journal of dermatological science 6: 159–64.

Kim JY, Huh K, Lee KY, Yang JM, Kim TJ. 2009. Nickel induces secretion of IFN-gamma by splenic natural killer cells. Experimental & molecular medicine 41:288–95; doi:10.3858/emm.2009.41.4.032.

Knight JA, Plowman MR, Hopfer SM, Sunderman FW Jr. 1991. Pathological reactions in lung, liver, thymus, and spleen of rats after subacute parenteral administration of nickel sulfate. Annals of clinical and laboratory science 21: 275–83.

Lisby S, Hansen LH, Menn T, Baadsgaard O. 1999. Nickel-induced proliferation of both memory and naive T cells in patch test-negative individuals. Clinical and experimental immunology 117: 217–22.

Miyazawa M, Ito Y, Yoshida Y, Sakaguchi H, Suzuki H. 2007. Phenotypic alterations and cytokine production in THP-1 cells in response to allergens. Toxicology in vitro : an international journal published in association with BIBRA 21:428–37; doi:10.1016/j.tiv.2006.10.005.

Obone E, Chakrabarti SK, Bai C, Malick MA, Lamontagne L, Subramanian KS. 1999. Toxicity and bioaccumulation of nickel sulfate in Sprague-Dawley rats following 13 weeks of subchronic exposure. Journal of toxicology and environmental health Part A 57: 379–401.

Thomas P, Barnstorf S, Summer B, Willmann G, Przybilla B. 2003. Immuno-allergological properties of aluminium oxide (Al2O3) ceramics and nickel sulfate in humans. Biomaterials 24: 959–66.

Nitrobenzene [CASRN 98-95-3]

Human Data

Data from epidemiology studies
No data were located.

In vitro data with cells or cell lines
No data were located.

Mode of action information
No data were located.

Rodent Data

Data from in vivo immunotoxicology or toxicology studies
Female B6C3F1 mice were exposed to 30, 100, or 300 mg/kg nitrobenzene for 14 days via gastric intubation. Hepatomegaly and splenomegaly were observed in mice that received 100 and 300 mg/kg nitrogenzene. Mild congestion in the red pulp areas of the spleen was noted in mice that received 100 mg/kg, while the spleen was dark red in those that received 300 mg/kg. Absolute and relative spleen weight were significantly increased (LOAEL = 100 mg/kg). Comparatively, absolute and relative thymus weights were increased only at 100 mg/kg. The number of bone marrow cells increased in a dose-dependent manner (LOAEL = 30 mg/kg). At the highest dose tested the increase was 60% above controls. DNA synthesis and the number of CFU-GM per femur also were increased (LOAELs = 30 mg/kg). In response to sheep erythrocytes, a significant increase in spleen weight (62%) and spleen cell number (29%) was observed at 300 mg/kg, when animals were sensitized four days after nitrobenzene exposure. Comparatively, a decrease in IgM AFCs were decreased (LOAEL = 100 mg/kg). When responses to sheep erythrocytes were observed (sensitization occurred 5 days after nitrobenzene exposure), spleen weight and cells were increased at 100 and 300 mg/kg. However, no effects on IgG AFC were noted. When 20 days lapsed between nitrobenzene exposure and sensitization to sheep erythrocytes, no effects were reported. No effect on delayed hypersensitivity was reported at any of the tested doses. Splenic proliferation responses induced by PHA and ConA were suppressed by exposure to nitrobenzene (LOAEL = 100 mg/kg). No effect on LPS-induced proliferation were reported. Responses to DBA/2 mice spleen cells also were decreased (LOAEL = 100 mg/kg). Using radiolabeled sheep erythrocytes, the phagocytic index was shown to be increased in a dose-dependent manner. The phagocytic activity of peritoneal cells also was increased in a dose-dependent manner (LOAEL = 300 mg/kg). The ability of spleen cells to lyse radioactivity from YAC-1 target cells also was evaluated. Nitrobenzene exposure produced a decrease in lysis capacity at 100 and 300 mg/kg at effector:target ratios of 100:1 and 30:1. Nitrobenzene did not affect host resistance to *Streptococcus pneumonia, Plasmodium berghei,* herpes simplex 2, or B16F10 melanoma. Comparatively, host resistance to *Listeria monocytogenes* was decreased. A challenge of 6 x 10^3 *L. monocytogenes* per

mouse killed 13%

of control animals and 57% of animals treated with 300 mg/kg nitrobenzene. A challenge with 1.2×10^4 *L. monocytogenes* increased animal death from 19% in controls to 100% at 100 mg/kg nitrobenzene and 86% at 300 mg/kg nitrobenzene (Burns et al. 1994).

In vitro data with cells or cell lines

No data were located.

Mode of action information

Two proposed targets of nitrobenzene are: (1) erythrocytes and (2) precursors to erythrocytes and other cells (e,g., granulocytes). The site of action is proposed to be the bone marrow. Additionally, effects on T-cell function may play a role in increased susceptibility to *L. monocytogenes* (Burns et al. 1994).

References

Burns LA, Bradley SG, White KL Jr, McCay JA, Fuchs BA, Stern M, et al. 1994. Immunotoxicity of nitrobenzene in female B6C3F1 mice. Drug and chemical toxicology 17:271–315; doi:10.3109/01480549409017862.

o-Benzyl-p-chlorophenol (BCP) [CASRN 120-32-1]

Human Data

Data from epidemiology studies

No data were located.

In vitro data with cells or cell lines

No data were located.

Mode of action information

No data were located.

Rodent Data

Data from in vivo immunotoxicology or toxicology studies

B6C3F1 mice were orally administered 100, 300, or 500 mg/kg BCP for 14 days. No effect on spleen or thymus weight were reported. No effect on delayed hypersensitivity response (to keyhold limpet hemocyanin), antibody response to sheep erythrocytes, serum IgM, IgA, or IgG levels, or splenic lymphocyte proliferation were noted. Absolute and relative liver weights were increased at the highest dose group. Additionally, BCP-treated mice did not develop tumors after challenge with PYB6 tumor cells (vs. controls which had a 15% tumor incidence) (Birnbaum et al. 1986).

BCP produced contact hypersensitivity in female B6C3F1 mice (Stern et al. 1991).

In vitro data with cells or cell lines

No data were located.

Mode of action information

No data were located.

References

Birnbaum LS, Deskin R, Grumbein SL, Kurtz P, Fowler KL, Peters AC. 1986. Prechronic toxicity of o-benzyl-p-chlorophenol in rats and mice. Fundamental and applied toxicology : official journal of the Society of Toxicology 7: 615–25.

Stern ML, Brown TA, Brown RD, Munson AE. 1991. Contact hypersensitivity response to o-benzyl-p-chlorophenol in mice. Drug and chemical toxicology 14:231–42; doi:10.3109/01480549109002186.

Perflouorooctanoic Acid (PFOA) [CASRN 335-67-1]

Human Data

Data from epidemiology studies

Several studies have suggested that prenatal PFOA exposure is linked to immunosuppressive and immunotoxicant effects observed in offspring. Granum and colleagues (2013) reported that maternal PFOA blood levels, collected at birth, were positively associated with decreased rubella antibody-levels ($\beta = -0.40$) and an increased number of common cold episodes in children from 0-3 and 2-3 years old. Cord blood IgE levels also were suppressed in female infants with high maternal PFOA levels. However, no effects on number of 18 month-old infants with allergies (e.g., food allergy, eczema) or infections (e.g., otitis media, pneumonia, skin infections, chicken pox) were noted (Okada et al. 2012). Okada and colleagues noted that while the correlation between fetal PFOA levels and the evaluated endpoints were not available, the results suggest that PFOA produced immunosuppressive effects after prenatal exposure.

A positive association between serum PFOA in adults and development of ulcerative colitis also was reported. However, a positive association with other autoimmune diseases, such as Type 1 diabetes, lupus, multiple sclerosis, Chron's disease, and rheumatoid arthritis, was not observed (Steenland et al. 2013).

Chang and colleagues (2016) conducted a systematic review to summarize and evaluate epidemiological literature on PFOA and perfluorooctanesulfonate (PFOS) with relation to evaluated immune endpoints. Endpoints evaluated included immune biomarker levels (e.g., IgE levels, white blood cell count, and C-reactive protein), immune gene expression patterns, atopic or allergic disorders (e.g., asthma, eczema, and food allergy), infectious disease (e.g., common cold), vaccine response, and autoimmune and inflammatory conditions (e.g., ulcerative colitis, rheumatoid arthritis, and osteoarthritis). The authors stated that the totality of the data limited development of a conclusion on the causal relationship between PFOA and/or PFOS exposure and evaluated endpoints due to inconsistent results and confounding factors.

In vitro data with cells or cell lines

Studies with human cells or human-derived cell lines indicate that PFOA modulates cell activation and cytokine production. In human PBMC, PFOA significantly increased the percentage of viable cells at concentrations <125 µg/mL. At higher concentrations (250 and 500 µg/mL), a significant decrease in cell viability was reported (values not reported). No effects on T-cell proliferation (NOAEL = 1 µg/mL) or, TNF-α or IL-6 release (NOAEL = 1 µg/mL) were noted. PFOA also increased monocyte differentiation in HL-60 cells (LOAEL = 100 µg/mL) (Brieger et al. 2011). Comparatively, PFOA decreased TNF-α, IL-4, and IL-10 (LOAEL = 1 µg/mL, 10 and 10 pg/mL, respectively) in peripheral leukocytes. PFOA also decreased TNF-α (LOAEL = 10 µg/mL) production in THP-1 cells (value not reported). PFOA did not affect IL-2 production in Jurkat cells (value not reported) (NOAEL = 0.005 µg/mL) (Corsini et al. 2011, 2012; Midgett et al. 2015).

Mode of action information

Direct modulation of NF-□B has been implicated in modulation of cytokine production and secretion (Corsini et al. 2012). PFOA interaction with the PPARα receptor also was implicated in immunomodulatory effects in human cells. Receptor interaction was associated with reduced p65 phosphorylation and NF-□B-mediated transcription (Corsini et al. 2011). The extent the role of PPARα receptor activation plays in human effects is unclear given the low level of human receptor expression (Corsini et al. 2014).

Rodent Data

Data from in vivo immunotoxicology or toxicology studies

Animal studies suggest that PFOA exposure can affect innate and adaptive immune functions *in vivo*. Dietary exposure to PFOA (0.02% w/w) for 7 days significantly decreased spleen and thymus weight, and splenocyte and thymocyte levels in wild-type C57Bl/6 mice. Spleen weight and splenocyte numbers were not affected in PPARα-null mice (Yang et al. 2002).

Table 1. Data from Yang et al. (2002)

Group and Treatment	Body weight (g)	Spleen weight (g)	Splenocyte number (x 10^6)	Thymus weight (g)	Thymocyte number (x 10^6)
Wild-type mice					
None	24.5 ± 1.58	0.082 ± 0.006	68.8 ± 16.8	0.061 ± 0.014	81.0 ± 28.2
PFOA	21.0 ± 0.74**	0.050 ± 0.001***	15.3 ± 5.84***	0.013 ± 0.001***	12.8 ±7.98***
PPARα-null mice					
None	23.6 ± 2.9	0.064 ± 0.021	84.0 ± 19.3	0.054 ± 0.006	88.3 ± 7.04
PFOA	23.5 ± 1.0†	0.054 ± 0.015	73.8 ± 26.2†††	0.033 ± 0.005***†††	54.0 ± 12.7**†††

All values are means ± SEM for four animals in each group. **P<0.01, ***P<0.001 compared to the corresponding control.
†P<0.05, †††P<0.001 compared to the corresponding wild-type group.

Dose response studies in C57Bl/6N mice reported that PFOA decreased absolute and relative spleen weights (LOAEL = 7.5 and 15 mg/kg/day, respectively) and absolute and relative thymus weights (LOAEL =15 mg/kg/day for both endpoints). Organ weight effects were generally reversed by 15 days after exposure was terminated (DeWitt et al. 2008). No effect on organ weights was reported in PPARα knockout mice treated with 7.5 or 30 mg/kg/day PFOA for 15 days (DeWitt et al. 2016).

PFOA exposure in drinking water was associated with reduced IgM antibody titers in C57Bl/6J and C57Bl/6N mice (DeWitt et al. 2008, 2016). Removal of the adrenal glands in C57Bl/6N mice did not reverse reductions in IgM antibody titer levels, suggesting that the observed suppression was not in response to corticosterone production (DeWitt et al. 2009).

Modulation of the complement system was observed in C57Bl/6 mice administered PFOA-treated diets. In mice provided diets containing PFOA for 10 days, activity of the classical and

alternative pathways of the complement system was decreased (N/LOAEL = 0.01%/0.02%, respectively). Serum C3 levels also was decreased by PFOA (N/LOAEL = 0.01%/0.02%, respectively). Results showed that PFOA-induced hepatotoxicity was associated with activation of the complement system (Botelho et al. 2015).

Dietary PFOA (0.02% w/w) for 10 days significantly decreased total white blood cell count (72%) and number of macrophages in the bone marrow (12.2%) (Qazi et al. 2009). Exposure of mice to 0.002% PFOA for 10 days modulated levels of intrahepatic immune cells. The total number of all leukocytes (CD45+) was increased 2-fold in treated mice. Additionally, changes in cell numbers other cell types also were noted (e.g., granulocytes and myeloid suppressor cells). Hepatic levels of TNF-α (33%), IFN-γ (37%), and IL-4 (31%) were decreased in treated mice; IL-6 levels were not affected (Qazi et al. 2010).

Hu and colleagues reported effects in offspring of dams exposed to PFOA. Dams were gavaged with 0.02, 0.2, or 2 mg/kg PFOA from before pregnancy to PND 21. Splenic CD4+CD25+Foxp3+ T cells was decreased by 22% in exposed offspring (LOAEL = 2 mg/kg) (Hu et al. 2012).

In vitro data with cells or cell lines

Reduced lymphocyte proliferation was observed in cells isolated from C57Bl/6 mice treated with diets containing 0.02% PFOA for 7 days. No effect was observed in lymphocytes isolated from PPARα-null mice also provided diets containing 0.02% PFOA (values not provided) (Yang et al. 2002). Increased *ex vivo* production of TNF-α in cells isolated from peritoneal cavity (2.2-fold) and bone marrow (1.7-fold), and IL-6 in cells isolated from peritoneal cavity (2.6-fold) was observed in mice treated with 0.02% dietary PFOA for 10 days. Comparatively, TNF-α production was decreased (0.8-fold) in cells isolated from spleen of treated animals (Qazi et al. 2009). IgM or IFN-γ production levels were not modulated in intrahepatic immune cells isolated from male C57Bl/6 mice provided diets with 0.002% (w/w) PFOA for 10 days (Qazi et al. 2010).

Ex vivo co-cultures of splenic CD4+CD25+ and CD4+CD25- T cells offspring gestationally and lactationally exposed to PFOA were assessed for effects on IL-10 production. Results showed IL-10 produced was significantly decreased at all doses 61%-75% in cells obtained from male offspring (LOAEL = 0.02 mg/kg). *Ex vivo* measurement of autoreactivity antibodies in female mice gestationally and lactationally exposed to 0.02 and 2 mg/kg PFOA showed an decrease (26%) in anti-ssDNA (Hu et al. 2012).

Mode of action information

PFOA suppresses T-cell-dependent and T-cell-independent antibody responses (DeWitt et al. 2012). The role of PPARα in PFOA-induced immunosuppression may be strain dependent (Corsini et al. 2014). PFOA-induced effects on humoral immunity may occur through effects on B-cell/plasma cell function (DeWitt et al. 2016). Direct effects on immune cells also are a proposed mode of action of PFOA (Corsini et al. 2014).

The lack of impact of removal of the adrenal gland on PFOA-induced inhibition of IgM antibody titer levels suggests that the observed effects are not dependent on elevated corticosterone levels in mice (DeWitt et al. 2009).

Effects on lymphoid organ weights and measures of immune function (i.e., thymus and spleen) indicate that they are differentially sensitive to PFOA effect. The biological basis for this difference is not known (DeWitt et al. 2016).

References

Botelho SC, Saghafian M, Pavlova S, Hassan M, DePierre JW, Abedi-Valugerdi M. 2015. Complement activation is involved in the hepatic injury caused by high-dose exposure of mice to perfluorooctanoic acid. Chemosphere 129:225–231; doi:10.1016/j.chemosphere.2014.06.093.

Brieger A, Bienefeld N, Hasan R, Goerlich R, Haase H. 2011. Impact of perfluorooctanesulfonate and perfluorooctanoic acid on human peripheral leukocytes. Toxicol Vitro Int J Publ Assoc BIBRA 25:960–968; doi:10.1016/j.tiv.2011.03.005.

Chang ET, Adami H-O, Boffetta P, Wedner HJ, Mandel JS. 2016. A critical review of perfluorooctanoate and perfluorooctanesulfonate exposure and immunological health conditions in humans. Crit Rev Toxicol 46:279–331; doi:10.3109/10408444.2015.1122573.

Corsini E, Avogadro A, Galbiati V, dell'Agli M, Marinovich M, Galli CL, et al. 2011. In vitro evaluation of the immunotoxic potential of perfluorinated compounds (PFCs). Toxicol Appl Pharmacol 250:108–116; doi:10.1016/j.taap.2010.11.004.

Corsini E, Luebke RW, Germolec DR, DeWitt JC. 2014. Perfluorinated compounds: emerging POPs with potential immunotoxicity. Toxicol Lett 230:263–270; doi:10.1016/j.toxlet.2014.01.038.

Corsini E, Sangiovanni E, Avogadro A, Galbiati V, Viviani B, Marinovich M, et al. 2012. In vitro characterization of the immunotoxic potential of several perfluorinated compounds (PFCs). Toxicol Appl Pharmacol 258:248–255; doi:10.1016/j.taap.2011.11.004.

DeWitt JC, Copeland CB, Luebke RW. 2009. Suppression of humoral immunity by perfluorooctanoic acid is independent of elevated serum corticosterone concentration in mice. Toxicol Sci Off J Soc Toxicol 109:106–112; doi:10.1093/toxsci/kfp040.

DeWitt JC, Copeland CB, Strynar MJ, Luebke RW. 2008. Perfluorooctanoic acid-induced immunomodulation in adult C57BL/6J or C57BL/6N female mice. Environ Health Perspect 116:644–650; doi:10.1289/ehp.10896.

DeWitt JC, Peden-Adams MM, Keller JM, Germolec DR. 2012. Immunotoxicity of perfluorinated compounds: recent developments. Toxicol Pathol 40:300–311; doi:10.1177/0192623311428473.

DeWitt JC, Williams WC, Creech NJ, Luebke RW. 2016. Suppression of antigen-specific antibody responses in mice exposed to perfluorooctanoic acid: Role of PPARα and T- and B-cell targeting. J Immunotoxicol 13:38–45; doi:10.3109/1547691X.2014.996682.

Granum B, Haug LS, Namork E, Stølevik SB, Thomsen C, Aaberge IS, et al. 2013. Pre-natal exposure to perfluoroalkyl substances may be associated with altered vaccine antibody levels and immune-related health outcomes in early childhood. J Immunotoxicol 10:373–379; doi:10.3109/1547691X.2012.755580.

Hu Q, Franklin JN, Bryan I, Morris E, Wood A, DeWitt JC. 2012. Does developmental exposure to perflurooctanoic acid (PFOA) induce immunopathologies commonly observed in neurodevelopmental disorders? Neurotoxicology 33:1491–1498; doi:10.1016/j.neuro.2012.10.016.

Midgett K, Peden-Adams MM, Gilkeson GS, Kamen DL. 2015. In vitro evaluation of the effects of perfluorooctanesulfonic acid (PFOS) and perfluorooctanoic acid (PFOA) on IL-2 production in human T-cells. J Appl Toxicol JAT 35:459–465; doi:10.1002/jat.3037.

Okada E, Sasaki S, Saijo Y, Washino N, Miyashita C, Kobayashi S, et al. 2012. Prenatal exposure to perfluorinated chemicals and relationship with allergies and infectious diseases in infants. Environ Res 112:118–125; doi:10.1016/j.envres.2011.10.003.

Qazi MR, Abedi MR, Nelson BD, DePierre JW, Abedi-Valugerdi M. 2010. Dietary exposure to perfluorooctanoate or perfluorooctane sulfonate induces hypertrophy in centrilobular hepatocytes and alters the hepatic immune status in mice. Int Immunopharmacol 10:1420–1427; doi:10.1016/j.intimp.2010.08.009.

Qazi MR, Bogdanska J, Butenhoff JL, Nelson BD, DePierre JW, Abedi-Valugerdi M. 2009. High-dose, short-term exposure of mice to perfluorooctanesulfonate (PFOS) or perfluorooctanoate (PFOA) affects the number of circulating neutrophils differently, but enhances the inflammatory responses of macrophages to lipopolysaccharide (LPS) in a similar fashion. Toxicology 262:207–214; doi:10.1016/j.tox.2009.06.010.

Steenland K, Zhao L, Winquist A, Parks C. 2013. Ulcerative colitis and perfluorooctanoic acid (PFOA) in a highly exposed population of community residents and workers in the mid-Ohio valley. Environ Health Perspect 121:900–905; doi:10.1289/ehp.1206449.

Yang Q, Xie Y, Alexson SEH, Nelson BD, DePierre JW. 2002. Involvement of the peroxisome proliferator-activated receptor alpha in the immunomodulation caused by peroxisome proliferators in mice. Biochem Pharmacol 63: 1893–1900.

Toluene [CASRN 108-88-3]

Human Data

Data from epidemiology studies

No difference was noted in lymphocyte counts between individuals with or without toluene exposure (Akbas et al. 2004).

In vitro data with cells or cell lines

No data were located.

Mode of action information

No data were located.

Rodent Data

Data from in vivo immunotoxicology or toxicology studies

Male C3H mice were exposed to 9 ppm toluene (nose-only inhalation exposure) for 30 minutes on study days 0, 1, 2, 7, 14, 21, and 28. Mice also were immunized with ovalbumin. Toluene exposure significantly increased total cell (3-fold) and macrophage (3.1-fold) count in BAL 24 hours after final exposure. No effect on lymphocyte count was noted. BDNF level in BAL was increased in toluene-exposed mice that were immunized with ovalbumin (data in figure). Splenic ratio of CD4 and CD8 cells in control and toluene-exposed mice were not significantly different; 3.95 and 4.14, respectively. Treatment with ani-CD4 antibody decreased the ratios to 0.65 and 0.49, respectively. Toluene exposure significantly increased plasma levels of nerve growth factor (data in figure), but did not increase plasma BDNF levels (data not provided) (Fujimaki et al. 2009).

Male C57BL/10 and B10.BR/Sg mice were inhalationally exposed to 0, 5, and 50 ppm toluene for 6 hours per day, 5 days per week for 6 weeks. Subgroups of control and treated mice were administered ovalbumin prior to exposure. Toluene exposure did not impact ConA- or LPS-induced proliferation of spleen cells from C57BL/10 mice. While no effect of ConA was noted in B10.BR/Sg (not treated with ovalbumin) mice spleen cells, 50 ppm toluene significantly increased the LPS-induced proliferation of spleen cells. Comparatively, 50 ppm toluene significantly decreased spleen cell proliferation in B10.BR/Sg mice treated with ovalbumin (data in graphs). Toluene did not alter expression of CD3, CD19, and CD11b (data not provided).
Forkhead box P3 (Foxp3) transcription was significantly increased in spleen cells from B10.BR/Sg mice exposed to 5 ppm toluene and ovalbumin, when compared to controls and those not treated with ovalbumin. No effect on GATA3 or T-bet expression was noted (Fujimaki et al. 2010).

Pregnant C3H/HeN mice were exposed to 50 ppm toluene via inhalation on GD 14-18. Additionally, male offspring of unexposed dams were exposed to 50 ppm toluene on PND 2-6 or 8-12. The following table summarizes the effects observed in male offspring on PND 21.

Table 1. Summary of effects in male offspring

Origin	Biomarker	GD 14-18	PND 2-6	PND 8-12
Plasma	IgG2a	No effect	Decrease	Increase
	IgG1	Decrease	Decrease	Decrease
Spleen	CD4+ lymphocyte subset	No effect	Decrease	Decrease
	CD8+ lymphocyte subset	No effect	No effect	Decrease
	T-bet mRNA	No effect	Decrease	Decrease
	Foxp3 mRNA	No effect	Decrease	Decrease
	GATA3 mRNA	No effect	No effect	No effect

On PND 42, IgG2a levels were decreased in mice exposed to 50 ppm toluene on PND 8-12. No effect on IgG1 was noted. CD19+ B-lymphocytes and CD4+ T-lymphocytes were significantly decreased, while CD3+ T-lymphocytes were increased at PND 42 after exposure on PND 8-12. Additionally, T-bet expression was significantly decreased, while no effects on GATA3 or Foxp3 mRNA expression were reported (Win-Shwe et al. 2012a).

Pregnant C3H/HeN mice were exposed to 5 or 50 ppm toluene via inhalation on GD 14-18. Additionally, male offspring of unexposed dams were exposed to 5 or 50 ppm toluene on PND 2-6 or 8-12. In the hippocampus of PND 21 male offspring, TNF-α and NF-κB mRNA were significantly increased in mice exposed to 50 ppm on PND 2-6 when compared to controls (data in graphs). TNF-α, CCL3, and NF-κB were increased in mice exposed to 5 ppm on PND 8-12 (data in graphs) (Win-Shwe et al. 2012b).

In vitro data with cells or cell lines

Toluene (500 µM) exposure significantly increased ConA- (1.8-fold) and LPS- (2.1-fold) induced proliferation of spleen cells from female C57BL/6 mice. However, at the same concentration toluene did not modulate NK activity or suppress CTL formation (Grayson and Gill 1986).

Mode of action information

Low-level (5 ppm) inhalational exposure to toluene activates the STAT6, STAT5, and Foxp3 signaling pathway to enhance Th2-related and T_{reg}-related responses in B10.BR/Sg mice treated with ovalbumin (Fujimaki et al. 2010). Toluene also enhanced NF-□B, STAT5, and NF-AT in thymus cells of C3H/HeN mice inhalationally exposed to toluene (Liu et al. 2010).

Toluene modulation of IL-2 synthesis, after oral exposure, may play a role in observed immunotoxic effects (Hsieh et al. 1989).

References

Akbas E, Derici E, Soylemez F, Kanik A, Polat F. 2004. An investigation of effects of toluene and cigarette smoking on some blood parameters and lymphocyte life span. Cell biology and toxicology 20: 33–40.
Fujimaki H, Win-Shwe TT, Yamamoto S, Kunugita N, Yoshida Y, Arashidani K. 2010. Different sensitivity in expression of transcription factor mRNAs in congenic mice following

exposure to low-level toluene. Inhalation toxicology 22:903–9; doi:10.3109/08958378.2010.494256.

Fujimaki H, Win-Shwe TT, Yamamoto S, Nakajima D, Goto S. 2009. Role of CD4(+) T cells in the modulation of neurotrophin production in mice exposed to low-level toluene. Immunopharmacology and immunotoxicology 31:146–9; doi:10.1080/08923970802504762.

Grayson MH, Gill SS. 1986. Effect of in vitro exposure to styrene, styrene oxide, and other structurally related compounds on murine cell-mediated immunity. Immunopharmacology 11: 165–73.

Hsieh GC, Sharma RP, Parker RD. 1989. Immunotoxicological evaluation of toluene exposure via drinking water in mice. Environmental research 49: 93–103.

Liu J, Yoshida Y, Kunugita N, Noguchi J, Sugiura T, Ding N, et al. 2010. Thymocytes are activated by toluene inhalation through the transcription factors NF-kappaB, STAT5 and NF-AT. Journal of applied toxicology : JAT 30:656–60; doi:10.1002/jat.1536.

Win-Shwe TT, Kunugita N, Nakajima D, Yoshida Y, Fujimaki H. 2012a. Developmental stage-specific changes in immunological biomarkers in male C3H/HeN mice after early life toluene exposure. Toxicology letters 208:133–41; doi:10.1016/j.toxlet.2011.10.015.

Win-Shwe TT, Kunugita N, Yoshida Y, Nakajima D, Tsukahara S, Fujimaki H. 2012b. Differential mRNA expression of neuroimmune markers in the hippocampus of infant mice following toluene exposure during brain developmental period. Journal of applied toxicology : JAT 32:126–34; doi:10.1002/jat.1643.

Tributyltin Chloride (TBTC) [CASRN 1461-22-9]

Human Data

Data from epidemiology studies

No data were located.

In vitro data with cells or cell lines

TBTC dose-dependently decreased the percentage of colony forming unit-granulocyte macrophage (CFU-GM) colonies at concentrations ranging from 0.001 to 3.3 µM (data not provided) (Carfi et al. 2007).

Cytokine release from human lymphocytes was assessed in whole blood obtained from three donors. IFN-γ was evaluated in blood from two donors after PHA stimulation for 72 hours. Comparatively, TNF-α was evaluated in blood from three donors after LPS stimulation for 72 hours. Overall, IFN-γ and TNF-α was modulated (i.e., either increase or decrease release) in all tested samples (Carfi et al. 2007).

Long-term cultures of human bone marrow cells were incubated with 0.001 µM TBTC in the presence or absence of a cytokine mixture for 7 or 14 days. A significant decrease in the percentage of CD19+CD22+ cells, in the absence of effects on the total lymphocyte population or percentage of T-cell subsets was reported after 7 and 14 days. Addition of cytokine mixture had no effect on TBTC effects. TBTC also induced cell death in CD19+ lymphocytes, in the absence of PPAR-γ receptor expression (Carfi et al. 2010).

The IC50s for cell viability in human LCLs or PBMCs were 0.25 and 0.33 µM, respectively. TBTC (0.1 µM) did not modulate TNF-α, IL-2, IL-4, or IL-10 release in LCLs pre-treated with phorbol 12-myristate 13-acetate/ionomycin stimulated cells. Comparatively, TBTC significantly decreased IL-6 and IFN-γ release (Markovic et al. 2015).

Mode of action information

In vitro toxicogenomic studies in Jurkat cells (human lymphoblastic T-cell line) showed that TBTC activated cellular stress response and retinoic-acid mediated response genes (Shao et al. 2013).

Rodent Data

Data from in vivo immunotoxicology or toxicology studies

In a 2-week study, male Wistar rats were provided diets containing 15, 50, or 150 ppm TBTC. A dose-related decrease in relative and absolute spleen and thymus weights were reported (LOAEL = 50 and 15 ppm, respectively). Concurrent to the change in thymus weight, a decrease in thymic cell counts also was observed (LOAEL = 50 ppm). However, no signs of increased lymphocyte destruction in the spleen was observed. A dose-related increase in relative liver weight was reported (LOAEL = 50 ppm). Decreased thymus weight also was observed in rats fed 100 ppm

TBTC for 4-weeks (43% of control weight); no effects on spleen or liver weight were noted (Snoeij et al. 1985).

In utero and lactational exposure effects of TBTC (0.025, 0.25, or 2.5 mg/kg/day) were evaluated in Sprague-Dawley rats. Dams were orally dosed with TBTC from GD 8 until weaning. After weaning, pups were orally exposed to the same dose as the dam until sacrifice (up to PND 90). In males, a significant decrease in spleen weight was only observed in pups treated with 0.25 mg/kg/day on PND 30. A significant decrease in thymus weight also was noted on PND 30 (LOAEL = 2.5 mg/kg/day) (Cooke et al. 2004). Serum IgM levels were increased in 30- and 60-day old female offspring, while IgA, IgM, IgG, and IgG2a levels were increased in 90-day old male rats (Tables 1 and 2) (Tryphonas et al. 2004).

Table 1. Serum IgM levels in 30- and 60-day old females†.

	30-day old females				60-day old females			
	Control	0.025 mg/kg/day	0.25 mg/kg/day	2.5 mg/kg/day	Control	0.025 mg/kg/day	0.25 mg/kg/day	2.5 mg/kg/day
IgM	51.6 ± 8.8	41.2 ± 6.8	39.6 ± 5.8	66.5 ± 9.9	34.0 ± 3.2	63.8 ± 5.8	68.1 ± 16.4	73.0 ± 15.0

†Values provided as pg Ig/mL serum × 10^4 (standard error of the mean ± standard error).

Table 2. Serum immunoglobulin levels in 90-day old males†

	Control	0.025 mg/kg/day	0.25 mg/kg/day	2.5 mg/kg/day	Pearson product moment correlation
IgA	32.0 ± 8.8	13.9 ± 3.3	9.7 ± 3.4*	11.9 ± 1.6	>0.05
IgM	46.2 ± 8.9	65.1 ± 4.9	69.6 ± 5.8	232.5 ± 90.1*	0.00168
IgG	96.8 ± 9.6	184.2 ± 86.7	194.6 ± 25.7*	314.1 ± 57.5*	0.0134
IgG1	41.5 ± 8.6	77.2 ± 28.6	85.2 ± 18.6	58.1 ± 15.9	>0.05
IgG2a	53.1 ± 5.7	59.1 ± 6.3	50.8 ± 4.6	31.3 ± 4.4*	0.00041
IgG2b	34.1 ± 3.3	39.1 ± 5.6	39.3 ± 3.5	31.6 ± 4.2	>0.05
IgG2c	13.6 ± 1.7	20.6 ± 3.6	41.0 ± 19.4	20.9 ± 2.2	>0.05

†Values provided as pg Ig/mL serum × 10^4 (standard error of the mean ± standard error).

* Significantly different from control.

The number and percentage of NK cells was increased in 30-day female and male offspring (LOAEL = 2.5 mg/kg/day). A dose-dependent increase in the number and percentage of NK cells also was noted in 90-day male rats. In 60-day female offspring an increase in the percentage of CD4+8+ T lymphocytes (LOAEL = 0.25 mg/kg/day). No anti-sheep erythrocyte IgM response or lymphoproliferative activity of splenocytes in response to mitogen stimulation was noted in 60-

day old female rats or 90-day old male rats (data not provided). Delayed-type hypersensitivity to oxazolone was increased in 90-day old male rats at 0.025 and 0.25 mg/kg/day and decreased at 2.50 mg/kg/day. Mean colony forming *L. monocytogenes* bacteria was non-linearly increased at 48 hours post-infection and statistically significant in pairwise comparisons (0.25 mg/kg/day) in 60-day old females. In 90-day old males, a non-linear dose–response trend 3 days after infection was reported. No effects in serum levels of IL-2, TNF-α, IFN-γ, and IL-1β were reported in males or females. A non-linear dose-response increase in NK activity in 60-day females was reported (Tryphonas et al. 2004).

Lactational exposure in mice to TBTC also impaired innate immunodefenses in offspring. C57BL/6 pregnant mice were given drinking water with 15 or 50 µg/mL TBTC from parturition to weaning. Clearance of *Escherichia coli* K-12 from the peritoneal cavity and spleen of offspring treated with 15 µg/mL TBTC was significantly decreased (Kimura et al. 2005).

ICR mice were orally dosed with 0.5, 4, or 20 mg/kg TBTC for 28 days. Relative spleen and thymus weights were significantly decreased at the highest dose tested (46% and 59% decrease, respectively). TBTC also decreased the number of plaque forming cells in response to exposure to sheep red blood cells (LOAEL = 4 mg/kg). TBTC also suppressed delayed-type hypersensitivity response to sheep red blood cells when assessed 24 and 48 hours after injection (LOAEL = 4 mg/kg). TBTC suppressed T-lymphocyte proliferation in a dose dependent manner (LOAEL = 20 mg/kg). Increased percentage of early- and late-stage thymocyte apoptosis, and expression of Fas protein expression in proteins also were noted (LOAEL = 4 mg/kg) (Chen et al. 2011).

Esophageal tubing of male C3H/Hen mice with 10 or 100 ppm TBTC for 1 week was associated with decreased NK activity. NK activities were inhibited 36% to 46% at effector:target (YAC-1 cells) ratios of 25:1 and 50:1, respectively. A significant decrease in the percentage of large granular lymphocytes (~60%) also was noted 1 week after end of treatment (Ghoneum et al. 1990).

In vitro data with cells or cell lines

Neutrophils and macrophages from mice lactationally exposed to TBTC (15 or 50 µg/mL) were isolated from peritoneal exudates. Bacterial binding to isolated neutrophils from offspring treated with 50 µg/mL TBTC was significantly decreased (data not provided). Comparatively, bacterial binding was increased in macrophages isolated from offspring treated with 50 µg/mL TBTC. Decreased phagocytosis (LOAEL = 15 µg/mL) and killing activities (15 µg/mL) only were observed in neutrophils. No effect on IL-1β, IL-6, or TNF-α production was noted from macrophages or neutrophils. MCP-1 production was significantly increased in neutrophils isolated from offspring treated with 50 µg/mL TBTC (Kimura et al. 2005).

Rat and mouse spleen cells were treated with TBTC for 24 hours and then LPS or PHA for 48 hours. Then, mitogen stimulation was assessed by 5-bromo-2'-deoxyuridine incorporation. TBTC inhibited cellular proliferation for both species; the inhibitory response was more potent in mice cells vs. rat cells (IC50 with LPS: 0.0025 vs. 0.007 µM, IC50 with PHA: 0.002 vs. 0.007 µM). TBTC also inhibited rat spleen proliferation that was stimulated by ConA (no data provided). TBTC also inhibited anti-CD3 antibody stimulation of mouse lymphocyte proliferation (IC50 > 0.1 µM) (Carfi et al. 2007).

NK activity was dose-dependently inhibited in splenic lymphocytes incubated with 0.01 to 1 ppm TBTC. The LOAEL values at effector:target ratios of 25:1 and 50:1 were 0.05 and 0.01 ppm, respectively. Decreased viability of splenic lymphocytes also was reported after exposure to TBTC (LOAEL = 0.1 ppm) (Ghoneum et al. 1990).

Mode of action information

In vivo effects of TBTC on the thymus of orally treated rats are proposed to be due to the metabolite dibutyltin chloride (Snoeij et al. 1988).

The role of apoptosis is not clear. In one study the authors indicated that apoptosis does not appear to be involved in inhibition of immature thymocyte proliferation, which may lead to thymus atrophy (Gennari et al. 1997). In a separate study, the authors proposed oxidative stress plays a role in TBTC-caspase-dependent apoptosis in murine thymocytes (Sharma and Kumar 2014).

In vitro studies suggest that TBTC promotes Th2 polarization via depletion of glutathione in antigen-presenting cells, which leads to modulation of IL-10 and IL-12 production (Kato et al. 2006).

References

Carfi M, Bowe G, Pieters R, Gribaldo L. 2010. Selective inhibition of B lymphocytes in TBTC-treated human bone marrow long-term culture. Toxicology 276:33–40; doi:10.1016/j.tox.2010.06.012.

Carfi M, Gennari A, Malerba I, Corsini E, Pallardy M, Pieters R, et al. 2007. In vitro tests to evaluate immunotoxicity: a preliminary study. Toxicology 229:11–22; doi:10.1016/j.tox.2006.09.003.

Chen Q, Zhang Z, Zhang R, Niu Y, Bian X, Zhang Q. 2011. Tributyltin chloride-induced immunotoxicity and thymocyte apoptosis are related to abnormal Fas expression. International journal of hygiene and environmental health 214:145–50; doi:10.1016/j.ijheh.2011.01.008.

Cooke GM, Tryphonas H, Pulido O, Caldwell D, Bondy GS, Forsyth D. 2004. Oral (gavage), in utero and postnatal exposure of Sprague-Dawley rats to low doses of tributyltin chloride. Part 1: Toxicology, histopathology and clinical chemistry. Food and chemical toxicology : an international journal published for the British Industrial Biological Research Association 42: 211–20.

Gennari A, Potters M, Seinen W, Pieters R. 1997. Organotin-induced apoptosis as observed in vitro is not relevant for induction of thymus atrophy at antiproliferative doses. Toxicol Appl Pharmacol 147:259–266; doi:10.1006/taap.1997.8265.

Ghoneum M, Hussein AE, Gill G, Alfred LJ. 1990. Suppression of murine natural killer cell activity by tributyltin: in vivo and in vitro assessment. Environmental research 52: 178–86.

Kato T, Tada-Oikawa S, Takahashi K, Saito K, Wang L, Nishio A, et al. 2006. Endocrine disruptors that deplete glutathione levels in APC promote Th2 polarization in mice leading to the exacerbation of airway inflammation. European journal of immunology 36:1199–209; doi:10.1002/eji.200535140.

Kimura K, Kobayashi K, Naito H, Suzuki Y, Sugita-Konishi Y. 2005. Effect of lactational exposure to tributyltin chloride on innate immunodefenses in the F1 generation in mice. Bioscience, biotechnology, and biochemistry 69:1104–10; doi:10.1271/bbb.69.1104.

Markovic T, Gobec M, Gurwitz D, Mlinaric-Rascan I. 2015. Characterization of human lymphoblastoid cell lines as a novel in vitro test system to predict the immunotoxicity of xenobiotics. Toxicology letters 233:8–15; doi:10.1016/j.toxlet.2014.12.013.

Shao J, Katika MR, Schmeits PC, Hendriksen PJ, van Loveren H, Peijnenburg AA, et al. 2013. Toxicogenomics-based identification of mechanisms for direct immunotoxicity. Toxicological sciences : an official journal of the Society of Toxicology 135:328–46; doi:10.1093/toxsci/kft151.

Sharma N, Kumar A. 2014. Mechanism of immunotoxicological effects of tributyltin chloride on murine thymocytes. Cell biology and toxicology 30:101–12; doi:10.1007/s10565-014-9272-7.

Snoeij NJ, Penninks AH, Seinen W. 1988. Dibutyltin and tributyltin compounds induce thymus atrophy in rats due to a selective action on thymic lymphoblasts. Int J Immunopharmacol 10: 891–899.

Snoeij NJ, van Iersel AA, Penninks AH, Seinen W. 1985. Toxicity of triorganotin compounds: comparative in vivo studies with a series of trialkyltin compounds and triphenyltin chloride in male rats. Toxicol Appl Pharmacol 81: 274–286.

Tryphonas H, Cooke G, Caldwell D, Bondy G, Parenteau M, Hayward S, et al. 2004. Oral (gavage), in utero and post-natal exposure of Sprague-Dawley rats to low doses of tributyltin chloride. Part II: effects on the immune system. Food and chemical toxicology : an international journal published for the British Industrial Biological Research Association 42: 221–35.

Urethane [CASRN 51-79-6]

Human Data

Data from epidemiology studies

No data were located.

In vitro data with cells or cell lines

The IC50s for cell viability in human LCLs or PBMCs were 82,329 and 140,768 µM, respectively. Urethane (5000 µM) did not modulate TNF-α, IFN-γ, IL-2, IL-4, IL-6, or IL-10 release in LCLs pre-treated with phorbol 12-myristate 13-acetate/ionomycin stimulated cells (Markovic et al. 2015).

Urethane dose-dependently decreased the percentage of CFU-GM colonies at concentrations greater than 1000 µM (data provided in graph) (Carfi et al. 2007).

Cytokine release from human lymphocytes was assessed in whole blood obtained from four donors. IFN-γ was evaluated in blood from three donors after PHA stimulation for 72 hours. Comparatively, TNF-α was evaluated in blood from four donors after LPS stimulation for 72 hours. IFN-γ was modulated (i.e., either increase or decrease release) in a single tested sample. TNF-α was not modulated any of the tested samples (Carfi et al. 2007).

Mode of action information

No data were located.

Rodent Data

Data from in vivo immunotoxicology or toxicology studies

Inbred A/J mice were administered urethane (1 mg/g) via intraperitoneal injection. A biphasic response on splenic NK cell activity was noted. At one day after the injection activity was decreased 60%, activity then increased (decreased 35%), and then remained decreased until 14 days after exposure (decreased 98%). Spleen size was initially reduced, but then increased to control levels. Mitogen response (against YAC-1 or RL♂1 target cells) was initially depressed after urethane exposure and then returned to control levels (Gorelik and Herberman 1981a).

Inbred A/J mice (5-24 days old) were administered urethane (0.5 mg/g or 1 mg/g) up to 24 days old. In all tested groups, splenic NK activity was inhibited without effects on cellularity on spleens. Decreased NK activity remained until at least 8-10 weeks of age (Gorelik and Herberman 1981a).

Inbred A/J, CBA/J, and C57BL/6 mice were administered urethane (1 mg/g) via intraperitoneal injection. One day after injection, cytotoxicity (against YAC-1 target cells) of A/J and CBA/] spleen cells was significantly decreased (63% and 25%, respectively). Activity was similar to control levels at day 4. Activity then decreased in splenic cells from A/J mice (58%), while a

similar effect in cells from CBA/J mice was not observed. No effect on activity was observed in C57BL/6 mice (Gorelik and Herberman 1981b).

Female B6C3F1 mice were administered 1, 2, or 4 mg/g urethane over a 14-day period via intraperitoneal injection. Decreased spleen weight (decreased 47%) and thymic atrophy (decreased 40%) were observed at 4 mg/g. Splenic lymphoproliferative response to ConA was decreased at 4 mg/g (42%). Responses to PHA and spleen cells from DBA mice were similar to controls. Delayed hypersensitivity responses also were not affected by exposure to urethane. Serum immunoglobulin levels and antibody responses to sheep erythrocytes and LPS were decreased in mice administered 4 mg/kg (decreased 61% and 46%, respectively). Macrophage cytostasis of MBL-2 target cells was decreased (LOAEL = 1 mg/g). However, phagocytosis and bactericidal activity against *S. aureus* was not affected. Pluripotent stem cells proliferation was inhibited at all doses. Urethane decreased NK activity against all YAC-1 target to cell ratios at all doses (Luster et al. 1982).

C57BL/6J dams were subcutaneously injected with 0.05 or 0.1 mg/g urethane on GD 7-17. Offspring were evaluated 8 weeks after parturition. Increased relative spleen weight was reported for the litter at 0.05 mg/g urethane. When evaluated based on sex, only an increase in relative thymus weight was observed at 0.05 and 0.1 mg/g. Decreased white blood cell count was also observed (LOAEL = 0.05 mg/g). No effect on lymphoproliferative responses or NK cell activity was noted. However, a decrease in the levels of plaque forming cells in response to sheep erythrocytes was noted (LOAEL = 0.1 mg/g) (Luebke et al. 1986).

C57BL/6J offspring were subcutaneously injected with 0.2 mg/g urethane on PND 5-14. No effects on organ weight or lymphoproliferative responses were noted. NK cell activity was decreased at an effector:target (YAC-1) ratio of 50:1. Splenic cellularity was increased in female offspring and decreased in male offspring (Luebke et al. 1986).

Female C57BL/6J mice were subcutaneously injected with 1, 2, or 4 mg/g urethane. Significant reduction in absolute (LOAEL = 1 mg/g) and relative (data not provided) spleen weights were observed. Additionally, absolute thymus weight was decreased (LOAEL = 4 mg/g). Dose-dependent reduction in leukocyte number was noted, but differential counts of white blood cells were not altered. Lymphoproliferative responses, induced by ConA, PHA, and LPS, were suppressed by urethane (LOAELs = 1, 1, and 4 mg/g, respectively). were noted. Lymphoproliferative responses to allogenic cells (mitomycin C treated CBA/J mouse spleen cels) were not affected by urethane exposure. NK cell activity was not affected at any effector:target (YAC-1) ratio. Splenic cellularity of mice treated with urethane and sheep erythrocytes was decreased (LOAEL = 2 mg/g) without effects on PFC/spleen or PFC/splenocytes. Decreased DTH index (to keyhole limpet hemocyanin) was decreased in urethane treated mice (LOAEL = 4 mg/g) (Luebke et al. 1987).

mRNA expression of interleukins and TNF-α were evaluated in spleens of male Wistar rats exposed to 1500 mg/kg urethane. Increased expression of IL-6 was noted, while decreased expression of IL-1β and TNF-α were reported. No effects on IL-2 expression were observed (Bette et al. 2004).

Urethane (10%) did not deplete ear epidermis Ia-positive LCs after male BALB/c mice were treated with topical application. Similarly, urethane did not alter the density of β-glucuronidase-positive LC in C57BL mouse tails topically treated for 1 or 3 weeks (Halliday et al. 1988).

Urethane administration to pregnant ICR mice (1.5 mg/g subcutaneous injection on GD 10) produced a transient decrease in dam thymocyte cell count. At 3 days after treatment, a significant decrease in cell count was noted. By 5 days after treatment, the cell count had recovered to control levels (data in graph). A similar phenomenon was noted with thymocyte phenotypes; decrease in CD4+8+ thymocytes (88%) at day 3 after treatment was recovered by day 5. Transient changes in dam splenocyte cell count and splenocyte phenotype CD4+8-, CD4-8+, and CD4-8- also were reported. Gene expression analyses identified changes in spleen gene expression due to urethane exposure with or without immune stimulation (FCA). Increased expression of TGFβ3 was observed in the presence or absence of immune stimulation one day after treatment. IGF-I, IGF-II and IL-2 were also differentially expressed (Sharova et al. 2002).

In vitro data with cells or cell lines

Rat and mouse spleen cells were treated with urethane for 24 hours and then LPS or PHA for 48 hours. Then, mitogen stimulation was assessed by 5-bromo-2'-deoxyuridine incorporation. Urethane did not inhibit cellular proliferation in either species (data not provided). Urethane also did not modulate rat spleen proliferation that was stimulated by ConA or inhibit anti-CD3 antibody stimulation of mouse lymphocyte proliferation (data not provided) (Carfi et al. 2007).

Mode of action information

In vitro and *in vivo* studies suggest that urethane metabolism by cytochrome P450 is needed to produce the observed immunomodulatory effects (Cha et al. 2000). Macrophage effects are based on urethane effects on the inductive phase of immune responses (Foris et al. 1983).

References

Bette M, Schlimme S, Mutters R, Menendez S, Hoffmann S, Schulz S. 2004. Influence of different anaesthetics on pro-inflammatory cytokine expression in rat spleen. Laboratory animals 38:272–9; doi:10.1258/002367704323133655.

Carfi M, Gennari A, Malerba I, Corsini E, Pallardy M, Pieters R, et al. 2007. In vitro tests to evaluate immunotoxicity: a preliminary study. Toxicology 229:11–22; doi:10.1016/j.tox.2006.09.003.

Cha SW, Gu HK, Lee KP, Lee MH, Han SS, Jeong TC. 2000. Immunotoxicity of ethyl carbamate in female BALB/c mice: role of esterase and cytochrome P450. Toxicology letters 115: 173–81.

Foris G, Bojan F, Medgyesi GA, Szilagyi T. 1983. Effects of urethan on lymphokine-producing activity of lymphocytes and on some functions of peritoneal macrophages in rats. Agents and actions 13: 63–8.

Gorelik E, Herberman RB. 1981a. Inhibition of the activity of mouse natural killer cells by urethan. Journal of the National Cancer Institute 66: 543–8.

Gorelik E, Herberman RB. 1981b. Susceptibility of various strains of mice to urethan-induced lung tumors and depressed natural killer cell activity. Journal of the National Cancer Institute 67: 1317–22.

Halliday GM, Odling KA, Ruby JC, Muller HK. 1988. Suppressor cell activation and enhanced skin allograft survival after tumor promotor but not initiator induced depletion of cutaneous Langerhans cells. The Journal of investigative dermatology 90: 293–7.

Luebke RW, Riddle MM, Rogers RR, Rowe DG, Garner RJ, Smialowicz RJ. 1986. Immune function in adult C57BL/6J mice following exposure to urethan pre- or postnatally. Journal of immunopharmacology 8: 243–57.

Luebke RW, Rogers RR, Riddle MM, Rowe DG, Smialowicz RJ. 1987. Alteration of immune function in mice following carcinogen exposure. Immunopharmacology 13: 1–9.

Luster MI, Dean JH, Boorman GA, Dieter MP, Hayes HT. 1982. Immune functions in methyl and ethyl carbamate treated mice. Clinical and experimental immunology 50: 223–30.

Markovic T, Gobec M, Gurwitz D, Mlinaric-Rascan I. 2015. Characterization of human lymphoblastoid cell lines as a novel in vitro test system to predict the immunotoxicity of xenobiotics. Toxicology letters 233:8–15; doi:10.1016/j.toxlet.2014.12.013.

Sharova LV, Gogal RM Jr, Sharov AA, Chrisman MV, Holladay SD. 2002. Immune stimulation in urethane-exposed pregnant mice increases expression level of spleen leukocyte genes for TGFbeta3 GM-CSF and other cytokines that may play a role in reduced chemical-induced birth defects. International immunopharmacology 2: 1477–89.

Vanadium Pentoxide [CASRN 1314-62-1]

Human Data

Data from epidemiology studies

No data were located.

In vitro data with cells or cell lines

Vanadium pentoxide (25 – 400 µM) inhibited cell proliferation and induced cell apoptosis in a dose and time-related manner in the IL-2-independent human NK cell line, NK-92MI. Cell proliferation was maximally inhibited (78%) at 400 µM vanadium pentoxide, and the percentage of cells undergoing apoptosis increased at 12 and 24 hours of exposure (51.2 and 64.7%, respectively) as the concentration of vanadium pentoxide increased. IL-2, IL-10 and IFNγ secretion were all inhibited by vanadium pentoxide after 24 hours at the highest concentration tested. IL-2 secretion also was inhibited after 12 hours. Expression of CD25 significantly increased above background starting at 50 µM, reaching a maximal migration inhibitory factor (MIF) of 47.4% at 400 µM. A similar pattern was observed for IL-15Rα, with a maximal MIF of 55.2% at 400 µM. Fas expression began to increase at 100 µM and reached a maximal MIF of 48.9% at 400 µM, while FasL peaked at 200 µM (62.1%). Jak3 phosphorylation was increased at 12 and 24 hours after treatment with 200 and 400 µM vanadium pentoxide (data in figure), and intracellular staining showed a strong presence of pJak3 in the internal cell membranes after treatment. (Gallardo-Vera et al. 2016).

Mode of action information

Vanadium in the +2, +3, and +4 (but not the +5) valence states interacted with human FMLP-activated neutrophils and statistically significantly increased the formation of hydroxyl radicals, with additional augmentation observed in the presence of sodium azide (values not provided) (Fickl et al. 2006).

Vanadium pentoxide induced toxic effects on the IL-2-independent human NK cell line, NK-92MI, through dysregulation of signaling pathways mediated by IL-2 via increased PTEN and decreased SHP1 expression (Gallardo-Vera et al. 2018).

Rodent Data

Data from in vivo immunotoxicology or toxicology studies

Male F344 rats were exposed to vanadium pentoxide (100 µg V/m^3) via inhalation, 5 hours per day for 5 days. The animals were infected with Listeria following the 5-day exposure and the bacterial burden assessed at 24, 48 and 72 hours, post-infection. Vanadium pentoxide did not have any significant effect on Listeria burdens at any of the timepoints observed (Cohen et al. 2007).

Male CD-1 mice were exposed to vanadium pentoxide (0 or an average of 1436 µg/m^3) via inhalation, 1 hour per day, 2 times per week over 12 weeks. An increase in the number (3.8±0.12

vs. 2.1±0.12 µm per field) and the size (36±0.52 vs. 25±0.35 µm) of megakaryocytes in the

spleen was observed in vanadium pentoxide exposed mice, as compared to controls. These same types of changes were also observed in the bone marrow (values not provided). No statistical difference was observed in spleen weight between treated and control mice (Fortoul et al. 2008).

When male and female CD-1 mice were exposed to vanadium pentoxide (0 or 1.4 mg/m^3) using the same protocol as in Fortoul et al., 2008, a sex difference was observed in the expression of Ki-67, a specific proliferation marker for lymphocytes. The percentage of Ki-67 immunopositive lymphocytes increased in male mice (38.86, 41.75 and 41.91%) after 4, 8 and 12 weeks of exposure, respectively, with both cytoplasmic and nuclear expression of Ki-67 observed. In female mice, the percentage of proliferating lymphocytes increased only after the first week of exposure (34.87%) and the signal was observed only in the nucleus. Subsequent exposures did not produce significant changes in the percentage of proliferating cells in females. The authors concluded there is a role for sex hormones in potential protection against vanadium immunoxicity (Rodriguez-Lara et al. 2016).

Male CD-1 mice were exposed to vanadium pentoxide (0 or an average of 1.4 mg/m^3) via inhalation, 1 hour per day, 2 times per week over 12 weeks. Spleen weight of vanadium exposed animals peaked at 9 weeks (546 ± 45 vs. 274 ± 27 mg in controls) and progressively decreased afterwards (321 ± 39 mg at 12 weeks vs. 298 ± 35 mg in controls). The spleens of vanadium exposed animals had histological changes that included increased numbers of lymphocytes and megakaryocytes as compared to controls. The number of $CD19^+$ cells was also increased within the hyperplastic germinal node (values not provided) and the mean hepatitis B surface antigen levels in immunized control mice was greater than in the exposed hosts (OD=0.39 ± 0.03 vs. 0.11 ± 0.05). The authors concluded that vanadium pentoxide induces functional changes in the spleen which appear to result in effects on the humoral immune response (Pinon-Zarate et al. 2008).

Male CD-1 mice were exposed to vanadium pentoxide (0 or an average of 1.4 mg/m^3) via inhalation, 1 hour per day, 2 times per week over 4 weeks. The expression of CD11c in the thymic medulla was decreased in vanadium pentoxide exposed mice, as compared to controls (values not provided), based on immunohistochemistry. Flow cytometry also demonstrated a decrease in $CD11c^+$ and $MHC\text{-}II^+$ cells in vanadium pentoxide exposed mice, as compared to controls (values not provided). The decrease was both, in terms of number and in mean fluorescence intensity values (Ustarroz-Cano et al. 2012).

Male F344/N rats and female B6C3F1 mice were exposed to 0, 4,8, or 16 mg/m^3 vanadium pentoxide, via inhalation, 6 hours per day, 5 days per week for 16 days. Pulmonary inflammation was assessed via analysis of BAL fluid. Significant alterations in the percentage of recoverable macrophages and neutrophils (NOAEL 4 mg/m^3), and increased lung protein and lysozyme in male rats (LOAEL 4 mg/m^3) were observed. In female mice, an increase in lymphocytes, protein and lysozymes was observed (LOAEL 4 mg/m^3). No effects were observed on systemic immunity as evidenced by a normal response to *Klebsiella pneumoniae* (National Toxicology Program 2002).

The induction of pulmonary inflammation was examined in three different strains of mice [A/J (sensitive strain for pulmonary inflammation and carcinogenesis), BALB/c (intermediate

sensitivity), and C57Bl/6J (resistant)]. Mice were aspirated with vanadium pentoxide (4 mg/kg) or phosphate-buffered saline, four times per week, with BALF collected at 6 hours, and 1, 3, 6 and 21 days. In A/J mice, vanadium pentoxide increased BALF levels of total cells (95.7%) inflammatory markers (PMNs, macrophages and lymphocytes, 74.6, 99.5, and 623.8%, respectively). Levels of inflammatory chemokines (keratinocyte-derived chemokine, macrophage inflammatory protein-2 and monocyte chemoattractant protein 1), transcription factor activity (NFκB and c-Fos) and signaling pathway activation (MAPK) were increased with highest levels observed in A/J mice followed by BALB/c and then C57BL/6J mice (data in graphs). All results returned to baseline 21 days post exposure (Rondini et al. 2010).

In vitro data with cells or cell lines

No data were located.

Mode of action information

Rondini and colleagues (2010) reported that vanadium pentoxide impacts pulmonary levels of inflammatory markers, induction of chemokines, and modulation of transcription factors Alterations in macrophage mediated functions have been associated with vanadium exposure (Cohen et al. 1996).

The ability of several vanadium compounds to increase mRNA levels of cytokines in BALF was investigated in female CD rats. Rats received 42 or 420 µg of vanadium pentoxide or phosphate-buffered saline by intratracheal instillation. BALF was collected at times ranging from 1 hour to 10 days. Influx of neutrophils was significantly increased 24 hours after exposure to vanadium pentoxide and peaked 24-48 hours post exposure (data in graph). Macrophage inflammatory protein-2 mRNA expression levels were significantly elevated in vanadium pentoxide treated rats at 1 to 48-hour timepoints, as compared to controls (Pierce et al. 1996).

References

Cohen MD, McManus TP, Yang Z, Qu Q, Schlesinger RB, Zelikoff JT. 1996. Vanadium affects macrophage interferon-gamma-binding and -inducible responses. Toxicology and applied pharmacology 138:110–20; doi:10.1006/taap.1996.0104.

Cohen MD, Sisco M, Prophete C, Chen LC, Zelikoff JT, Ghio AJ, et al. 2007. Pulmonary immunotoxic potentials of metals are governed by select physicochemical properties: vanadium agents. Journal of immunotoxicology 4:49–60; doi:10.1080/15476910601119350.

Fickl H, Theron AJ, Grimmer H, Oommen J, Ramafi GJ, Steel HC, et al. 2006. Vanadium promotes hydroxyl radical formation by activated human neutrophils. Free radical biology & medicine 40:146–55; doi:10.1016/j.freeradbiomed.2005.09.019.

Fortoul TI, Pinon-Zarate G, Diaz-Bech ME, Gonzalez-Villalva A, Mussali-Galante P, Rodriguez-Lara V, et al. 2008. Spleen and bone marrow megakaryocytes as targets for inhaled vanadium. Histology and histopathology 23:1321–6; doi:10.14670/hh-23.1321.

Gallardo-Vera F, Diaz D, Tapia-Rodriguez M, Fortoul van der Goes T, Masso F, Rendon-Huerta E, et al. 2016. Vanadium pentoxide prevents NK-92MI cell proliferation and IFNgamma

secretion through sustained JAK3 phosphorylation. Journal of immunotoxicology 13:27–37; doi:10.3109/1547691x.2014.996681.

Gallardo-Vera F, Tapia-Rodriguez M, Diaz D, Fortoul van der Goes T, Montano LF, Rendon-Huerta EP. 2018. Vanadium pentoxide increased PTEN and decreased SHP1 expression in NK-92MI cells, affecting PI3K-AKT-mTOR and Ras-MAPK pathways. Journal of immunotoxicology 15:1–11; doi:10.1080/1547691x.2017.1404662.

National Toxicology Program. 2002. NTP toxicology and carcinogensis studies of vanadium pentoxide (CAS No. 1314-62-1) in F344/N rats and B6C3F1 mice (inhalation). National Toxicology Program technical report series 1–343.

Pierce LM, Alessandrini F, Godleski JJ, Paulauskis JD. 1996. Vanadium-induced chemokine mRNA expression and pulmonary inflammation. Toxicology and applied pharmacology 138:1–11; doi:10.1006/taap.1996.9999.

Pinon-Zarate G, Rodriguez-Lara V, Rojas-Lemus M, Martinez-Pedraza M, Gonzalez-Villalva A, Mussali-Galante P, et al. 2008. Vanadium pentoxide inhalation provokes germinal center hyperplasia and suppressed humoral immune responses. Journal of immunotoxicology 5:115–22; doi:10.1080/15476910802085749.

Rodriguez-Lara V, Muniz-Rivera Cambas A, Gonzalez Villalva A, Fortoul TI. 2016. Sex-based differences in lymphocyte proliferation in the spleen after vanadium inhalation. Journal of immunotoxicology 13:498–508; doi:10.3109/1547691x.2015.1134731.

Rondini EA, Walters DM, Bauer AK. 2010. Vanadium pentoxide induces pulmonary inflammation and tumor promotion in a strain-dependent manner. Particle and fibre toxicology 7:9; doi:10.1186/1743-8977-7-9.

Ustarroz-Cano M, Garcia-Pelaez I, Pinon-Zarate G, Herrera-Enriquez M, Soldevila G, Fortoul TI. 2012. CD11c decrease in mouse thymic dendritic cells after vanadium inhalation. Journal of immunotoxicology 9:374–80; doi:10.3109/1547691x.2012.673181.

Zinc Dimethyldithiocarbamate (ZDMDC) [CASRN 137-30-4]

Human Data

Data from epidemiology studies

No data were located.

In vitro data with cells or cell lines

ZDMDC induced cytotoxicity in purified NK cells from healthy donors. Exposure to 2.5 µM ZDMDC for 24 hours produced a 99% decrease in lytic function (against K562 target cells) and at 1 µM for 6 days produced a 96% decrease. When a preparation containing T- and NK-cells were exposed to 2.5 µM for 24 hours a 41% decrease in function was observed. Comparatively, a 6-day exposure to 1 µM ziram did not inhibit lytic function. (Whalen et al. 2003). Wilson and colleagues showed that concentrations as low as 125 nM decreased cytotoxic function of purified NK cells (Wilson et al. 2004).

ZDMDC significantly inhibited NK-92MI activity (against K562 target cells) in a dose- and concentration-dependent manner (LOAEL = 0.125 µM at 2 hours incubation). A similar dose- and concentration-dependent inhibition of NK activity was observed with human lymphokine activated killer cells (LOAEL = 0.125 µM at 2 hours incubation) (Li et al. 2012a).

Purified, human NK cells were exposed to ZDMDC (0.5-5 µM) for 1 hour. Then the cells were incubated for 24 or 48 hours, or 6 days in ZDMDC-free media. A decrease in NK activity was observed at 2.5 and 5 µM. The loss of activity lasted up to 6 days after exposure (Taylor et al. 2005).

ZDMDC (5 µg/mL) decreased LPS-induced TNF-α production in THP-1 cells (data in graph). ZDMDC (5 µg/mL) also blocked LPS-induced degradation of I□B (data in Western blot) (Corsini et al. 2006).

ZDMDC induced apoptosis and necrosis in U937, NK-92MI, NK-92CI, Jurkat, and human T cells. Of U937 cells treated with 2 µM ZDMDC, 49.3% were apoptotic and 18.5% were necrotic (Li et al. 2011). In Jurkat cells treated with 0.5 µM ZDMDC, 52.5% were apoptotic and 7.9% were late apoptotic/necrotic (Li et al. 2012c). In NK-92MI cells treated with 0.5 µM ZDMDC, 47.4% were apoptotic and 12.2% were late apoptotic/necrotic (Li et al. 2012b). In NK-92CI cells treated with 0.5 µM ZDMDC, 28.7% were apoptotic and 38.5% were necrotic (Li et al. 2014). Increased apoptosis and late apoptosis/necrosis also was observed in a time- and dose-dependent manner in isolated primary T-cells (data in graph) (Li et al. 2012c).

At concentrations ranging from 0.1 to 10 µg/mL, ZDMDC was not cytotoxic to lymphocyte cultures obtained from peripheral blood from healthy volunteers (Zenzen et al. 2001).

Mode of action information

Effects in U937, NK-92MI, and Jurkat cells were dose- and time-dependent. Increased DNA fragmentation, level of active caspase-3, and level of cytochrome c release from U937 and Jurkat

cells also were noted after ZDMDC exposure (Li et al. 2011, 2012c, 2012b, 2015). Increased levels of caspase-7, -8, and -9 also were detected in NK-92MI and Jurkat cells (Li et al. 2012c, 2012b).

ZDMDC-induced inhibition of NK and LAK activity was mediated, in part, by decreases in intracellular levels of Gr3/K, granulysin, perforin, granzyme (Gr) A, and GrB (Li et al. 2012a). Decreased levels of GrB was associated with activation of p38 while activation of p44/42 was associated with decreased levels of perforin (Taylor and Whalen 2011).

Rodent Data

Data from in vivo immunotoxicology or toxicology studies

The EC3 in the local lymph node assay was between 1.0% and 5.0% in female BALB/c mice (De Jong et al. 2002). ZDMDC also was identified as a skin sensitizer in the guinea pig maximization test (TS5 = 0.01%) (van Och et al. 2001).

In vitro data with cells or cell lines

ZDMDC inhibited murine (C57BL/6J) cytotoxic T lymphocyte activity in a dose- and concentration-dependent manner (LOAEL = 0.125 μM) (Li et al. 2012a).

ZDMDC (10 μM) decreased expression of pro-caspase-1 and NLRP3 in J774A.1 cells. Studies in RAW264.7 cells showed that 10 μM ZDMDC increased pro-caspase-1 degradation and not protein cleavage. ZDMDC also decreased LPS-induced production of IL-18 and IL-1β in bone marrow macrophages. Inhibition of LPS-induced IL-1β production occurred in a dose-dependent manner in J774A.1 (LOAEL = 5 μM) (Muroi and Tanamoto 2015).

J774A.1 cells were infected with *S. typhimurium* TA98 and then treated with ZDMDC. ZDMDC (1-10 μM) increased the number of infected bacteria in a concentration-dependent manner (LOAEL = 5 μM) (Muroi and Tanamoto 2015).

Mode of action information

ZDMDC increased intracellular level of zinc in rat thymic lymphocytes, which may be associated with induction of apoptosis (Kanemoto-Kataoka et al. 2015).

References

Corsini E, Viviani B, Birindelli S, Gilardi F, Torri A, Codeca I, et al. 2006. Molecular mechanisms underlying mancozeb-induced inhibition of TNF-alpha production. Toxicology and applied pharmacology 212:89–98; doi:10.1016/j.taap.2005.07.002.

De Jong WH, Tentij M, Spiekstra SW, Vandebriel RJ, Van Loveren H. 2002. Determination of the sensitising activity of the rubber contact sensitisers TMTD, ZDMC, MBT and DEA in a modified local lymph node assay and the effect of sodium dodecyl sulfate pretreatment on local lymph node responses. Toxicology 176: 123–34.

Kanemoto-Kataoka Y, Oyama TM, Ishibashi H, Oyama Y. 2015. Dithiocarbamate fungicides increase intracellular Zn(2+) levels by increasing influx of Zn(2+) in rat thymic lymphocytes. Chemico-biological interactions 237:80–6; doi:10.1016/j.cbi.2015.05.014.

Li Q, Kobayashi M, Kawada T. 2014. Carbamate pesticide-induced apoptosis and necrosis in human natural killer cells. Journal of biological regulators and homeostatic agents 28: 23–32.

Li Q, Kobayashi M, Kawada T. 2015. Carbamate pesticide-induced apoptosis in human T lymphocytes. International journal of environmental research and public health 12:3633–45; doi:10.3390/ijerph120403633.

Li Q, Kobayashi M, Kawada T. 2012a. Effect of ziram on natural killer, lymphokine-activated killer, and cytotoxic T lymphocyte activity. Archives of toxicology 86:475–81; doi:10.1007/s00204-011-0771-5.

Li Q, Kobayashi M, Kawada T. 2012b. Mechanism of ziram-induced apoptosis in human natural killer cells. International journal of immunopathology and pharmacology 25:883–91; doi:10.1177/039463201202500406.

Li Q, Kobayashi M, Kawada T. 2012c. Mechanism of ziram-induced apoptosis in human T lymphocytes. Archives of toxicology 86:615–23; doi:10.1007/s00204-011-0791-1.

Li Q, Kobayashi M, Kawada T. 2011. Ziram induces apoptosis and necrosis in human immune cells. Archives of toxicology 85:355–61; doi:10.1007/s00204-010-0586-9.

Muroi M, Tanamoto K. 2015. Zinc- and oxidative property-dependent degradation of pro- caspase-1 and NLRP3 by ziram in mouse macrophages. Toxicology letters 235:199–205; doi:10.1016/j.toxlet.2015.04.012.

Taylor TR, Tucker T, Whalen MM. 2005. Persistent inhibition of human natural killer cell function by ziram and pentachlorophenol. Environmental toxicology 20:418–24; doi:10.1002/tox.20127.

Taylor TR, Whalen MM. 2011. Ziram activates mitogen-activated protein kinases and decreases cytolytic protein levels in human natural killer cells. Toxicology mechanisms and methods 21:577–84; doi:10.3109/15376516.2011.578170.

van Och FM, Vandebriel RJ, Prinsen MK, De Jong WH, Slob W, van Loveren H. 2001. Comparison of dose-responses of contact allergens using the guinea pig maximization test and the local lymph node assay. Toxicology 167: 207–15.

Whalen MM, Loganathan BG, Yamashita N, Saito T. 2003. Immunomodulation of human natural killer cell cytotoxic function by triazine and carbamate pesticides.

Chemico-biological interactions 145: 311–9.

Wilson S, Dzon L, Reed A, Pruitt M, Whalen MM. 2004. Effects of in vitro exposure to low levels of organotin and carbamate pesticides on human natural killer cell cytotoxic function. Environmental toxicology 19:554–63; doi:10.1002/tox.20061.

Zenzen V, Fauth E, Zankl H, Janzowski C, Eisenbrand G. 2001. Mutagenic and cytotoxic effectiveness of zinc dimethyl and zinc diisononyldithiocarbamate in human lymphocyte cultures. Mutation research 497: 89–99.
