# Supplementary material for: An international validation study of the IL-2 Luc assay for evaluating the potential immunotoxic effects of chemicals on T cells and a proposal for reference data for immunotoxic chemicals
Source: Toxicol In Vitro. Author manuscript; Available in PMC 2022 Oct 11. (PMC9552337; doi:10.1016/j.tiv.2020.104832)
Supplement: 3 [file NIHMS1660497-supplement-3.docx]

Appendix 3 The additional references of immunotoxicological information of the chemicals used in Phase I and II studies.

1. Chen, S., Golemboski, K., Piepenbrink, M., et al., 2004. Developmental immunotoxicity of lead in the rat: influence of maternal diet. J Toxicol Environ Health A 67, 495-511.
2. Chikanza, L.C., Panayi, G.S., 1993. The effects of hydrocortisone on in vitro lymphocyte proliferation and interleukin-2 and -4 production in corticosteroid sensitive and resistant subjects. Eur J Clin Invest 23, 845-850.
3. Demenesku, J., Mirkov, I., Ninkov, M., et al., 2014. Acute cadmium administration to rats exerts both immunosuppressive and proinflammatory effects in spleen. Toxicology 326, 96-108.
4. Fernandez-Cabezudo, M.J., Ali, S.A., Ullah, A., et al., 2007. Pronounced susceptibility to infection by Salmonella enterica serovar Typhimurium in mice chronically exposed to lead correlates with a shift to Th2-type immune responses. Toxicol Appl Pharmacol 218, 215-226.
5. Goodwin, J.S., Atluru, D., Sierakowski, S., et al., 1986. Mechanism of action of glucocorticosteroids. Inhibition of T cell proliferation and interleukin 2 production by hydrocortisone is reversed by leukotriene B4. J Clin Invest 77, 1244-1250.
6. Goutet, M., Ban, M., Binet, S., 2000. Effects of nickel sulfate on pulmonary natural immunity in Wistar rats. Toxicology 145, 15-26.
7. Hansen, J.F., Nielsen, C.H., Brorson, M.M., et al., 2015. Influence of phthalates on in vitro innate and adaptive immune responses. PLoS One 10, e0131168.
8. Hemdan, N.Y., Emmrich, F., Adham, K., et al., 2005. Dose-dependent modulation of the in vitro cytokine production of human immune competent cells by lead salts. Toxicol Sci 86, 75-83.
9. Iavicoli, I., Marinaccio, A., Castellino, N., et al., 2004. Altered cytokine production in mice exposed to lead acetate. Int J Immunopathol Pharmacol 17, 97-102.
10. Kim, J.Y., Huh, K., Lee, K.Y., et al., 2009. Nickel induces secretion of IFN-gamma by splenic natural killer cells. Exp Mol Med 41, 288-295.
11. Kooijman, R., Devos, S., Hooghe-Peters, E., 2010. Inhibition of in vitro cytokine production by human peripheral blood mononuclear cells treated with xenobiotics: implications for the prediction of general toxicity and immunotoxicity. Toxicol In Vitro 24, 1782-1789.
12. Metushi, I.G., Uetrecht, J., 2014. Isoniazid-induced liver injury and immune response in mice. J Immunotoxicol 11, 383-392.
13. Pathak, N., Khandelwal, S., 2008. Comparative efficacy of piperine, curcumin and picroliv against Cd immunotoxicity in mice. Biometals 21, 649-661.
14. Ringerike, T., Ulleras, E., Volker, R., et al., 2005. Detection of immunotoxicity using T-cell based cytokine reporter cell lines ("Cell Chip"). Toxicology 206, 257-272.
15. Thomas, P., Barnstorf, S., Summer, B., et al., 2003. Immuno-allergological properties of aluminium oxide (Al2O3) ceramics and nickel sulfate in humans. Biomaterials 24, 959-966.
16. Tsuboi, I., Tanaka, H., Nakao, M., et al., 1995. Nonsteroidal anti-inflammatory drugs differentially regulate cytokine production in human lymphocytes: up-regulation of TNF, IFN-gamma and IL-2, in contrast to down-regulation of IL-6 production. Cytokine 7, 372-379.
17. Wagner, W., Walczak-Drzewiecka, A., Slusarczyk, A., et al., 2006. Fluorescent Cell Chip a new in vitro approach for immunotoxicity screening. Toxicol Lett 162, 55-70.
18. Wang, P., Wang, J., Sun, Y.J., et al., 2017. Cadmium and chlorpyrifos inhibit cellular immune response in spleen of rats. Environ Toxicol 32, 1927-1936.
