# Supplementary material for: An international validation study of the IL-2 Luc assay for evaluating the potential immunotoxic effects of chemicals on T cells and a proposal for reference data for immunotoxic chemicals
Source: Toxicol In Vitro. Author manuscript; Available in PMC 2022 Oct 11. (PMC9552337; doi:10.1016/j.tiv.2020.104832)
Supplement: 4 [file NIHMS1660497-supplement-4.docx]

Appendix 4. References for toxicological information of 60 chemicals

1. NTP 1993a. Toxicology and Carcinogenesis Studies of Acetaminophen (CAS No. 103-90-2) in F344 Rats and B6C3F1 Mice (Feed Studies). Natl Toxicol Program Tech Rep Ser 394, 1-274.
2. NTP 1993b. Toxicology and Carcinogenesis Studies of p-Nitroaniline (CAS No. 100-01-6) in B6C3F1 Mice (Gavage Studies). Natl Toxicol Program Tech Rep Ser 418, 1-203.
3. NTP 1995. Toxicology and Carcinogenesis Studies of Benzethonium Chloride (CAS No. 121-54-0) in F344/N Rats and B6C3F1 Mice (Dermal Studies). Natl Toxicol Program Tech Rep Ser 438, 1-220.
4. NTP 1996. Toxicology and Carcinogenesis Studies of Nickel Sulfate Hexahydrate (CAS No. 10101-97-0) in F344 Rats and B6C3F1 Mice (Inhalation Studies). Natl Toxicol Program Tech Rep Ser 454, 1-380.
5. NTP 1997. Toxicology and Carcinogenesis Studies of Salicylazosulfapyridine (CAS No. 599-79-1) in F344/N Rats and B6C3F1 Mice (Gavage Studies). Natl Toxicol Program Tech Rep Ser 457, 1-327.
6. NTP 2003.Toxicology and carcinogenesiss studies of citral (microencapsulated) (CAS No. 5392-40-5) in F344/N rats and B6C3F1 mice (feed studies). Natl Toxicol Program Tech Rep Ser, 1-268.
7. Almousa, L.A., Salter, A.M., Langley-Evans, S.C., 2018. Magnesium deficiency heightens lipopolysaccharide-induced inflammation and enhances monocyte adhesion in human umbilical vein endothelial cells. Magnes Res 31, 39-48.
8. Auli, M., Domenech, A., Andres, A., et al., 2012. Multiparametric immunotoxicity screening in mice during early drug development. Toxicol Lett 214, 200-208.
9. Beschorner, W.E., Namnoum, J.D., Hess, A.D., et al., 1987. Cyclosporin A and the thymus. Immunopathology. Am J Pathol 126, 487-496.
10. Bessler, H., Straussberg, R., Gurary, N., et al., 1996. Effect of dexamethasone on IL-2 and IL-3 production by mononuclear cells in neonates and adults. Arch Dis Child Fetal Neonatal Ed 75, F197-201.
11. Blanke, T.J., Little, J.R., Shirley, S.F., et al., 1977. Augmentation of murine immune responses by amphotericin B. Cell Immunol 33, 180-190.
12. Bruserud, O., Lundin, K., 1987. The effect of drugs used in anticoagulation therapy on T lymphocyte activation in vitro. II. Warfarin inhibits T lymphocyte activation. J Clin Lab Immunol 23, 169-173.
13. Bunn, T.L., Parsons, P.J., Kao, E., et al., 2001. Exposure to lead during critical windows of embryonic development: differential immunotoxic outcome based on stage of exposure and gender. Toxicol Sci 64, 57-66.
14. Bygbjerg, I.C., Svenson, M., Theander, T.G., et al., 1987. Effect of antimalarial drugs on stimulation and interleukin 2 production of human lymphocytes. Int J Immunopharmacol 9, 513-519.
15. Caren, L.D., Oven, H.M., Mandel, A.D., 1985. Dimethyl sulfoxide: lack of suppression of the humoral immune response in mice. Toxicol Lett 26, 193-197.
16. Cesario, T.C., Slater, L.M., Kaplan, H.S., et al., 1984. Effect of antineoplastic agents on gamma-interferon production in human peripheral blood mononuclear cells. Cancer Res 44, 4962-4966.
17. Chetty, K.N., Subba Rao, D.S., Drummond, L., et al., 1979. Cobalt induced changes in immune response and adenosine triphosphatase activities in rats. J Environ Sci Health B 14, 525-544.
18. Chikanza, L.C., Panayi, G.S., 1993. The effects of hydrocortisone on in vitro lymphocyte proliferation and interleukin-2 and -4 production in corticosteroid sensitive and resistant subjects. Eur J Clin Invest 23, 845-850.
19. de Abreu Costa, L., Henrique Fernandes Ottoni, M., Dos Santos, M.G., et al., 2017. Dimethyl Sulfoxide (DMSO) Decreases Cell Proliferation and TNF-alpha, IFN-gamma, and IL-2 Cytokines Production in Cultures of Peripheral Blood Lymphocytes. Molecules 22.
20. De Waal, E.J., Timmerman, H.H., Dortant, P.M., et al., 1995. Investigation of a screening battery for immunotoxicity of pharmaceuticals within a 28-day oral toxicity study using azathioprine and cyclosporin A as model compounds. Regul Toxicol Pharmacol 21, 327-338.
21. Dieter, M.P., Luster, M.I., Boorman, G.A., et al., 1983. Immunological and biochemical responses in mice treated with mercuric chloride. Toxicol Appl Pharmacol 68, 218-228.
22. Dupont, E., Huygen, K., Schandene, L., et al., 1985. Influence of in vivo immunosuppressive drugs on production of lymphokines. Transplantation 39, 143-147.
23. Dupuis, G., Martel, J., Bastin, B., et al., 1993. Microtubules are not an essential component of phytohemagglutinin-dependent signal transduction in Jurkat T lymphocytes. Cell Immunol 146, 38-51.
24. el Fouhil, A.F., Iskander, F.A., Turkall, R.M., 1993a. Effect of alternate-day hydrocortisone therapy on the immunologically immature rat. II: Changes in T- and B-cell areas in spleen. Toxicol Pathol 21, 383-390.
25. el Fouhil, A.F., Iskander, F.A., Turkall, R.M., 1993b. Effect of alternate-day hydrocortisone therapy on the immunologically immature rat. III: Changes in T- and B-cell areas in lymph nodes. Toxicol Pathol 21, 391-396.
26. el Fouhil, A.F., Turkall, R.M., 1993. Effect of alternate-day hydrocortisone therapy on the immunologically immature rat. I: Effect on blood cell count, immunoglobulin concentrations, and body and organ weights. Toxicol Pathol 21, 377-382.
27. Exon, J.H., Koller, L.D., Talcott, P.A., et al., 1986. Immunotoxicity testing: an economical multiple-assay approach. Fundam Appl Toxicol 7, 387-397.
28. Ezendam, J., Hassing, I., Bleumink, R., et al., 2004. Hexachlorobenzene-induced Immunopathology in Brown Norway rats is partly mediated by T cells. Toxicol Sci 78, 88-95.
29. Freed, B.M., Lempert, N., Lawrence, D.A., 1989. The inhibitory effects of N-ethylmaleimide, colchicine and cytochalasins on human T-cell functions. Int J Immunopharmacol 11, 459-465.
30. Freed, B.M., Rapoport, R., Lempert, N., 1987. Inhibition of early events in the human T-lymphocyte response to mitogens and alloantigens by hydrogen peroxide. Arch Surg 122, 99-104.
31. Fujiwara, M., Mitsui, K., Yamamoto, I., 1990. Inhibition of proliferative responses and interleukin 2 productions by salazosulfapyridine and its metabolites. Jpn J Pharmacol 54, 121-131.
32. Gabryel, B., Labuzek, K., Malecki, A., et al., 2004. Immunophilin ligands decrease release of pro-inflammatory cytokines (IL-1beta, TNF-alpha and IL-2 in rat astrocyte cultures exposed to simulated ischemia in vitro. Pol J Pharmacol 56, 129-136.
33. Garly, M.L., Trautner, S.L., Marx, C., et al., 2008. Thymus size at 6 months of age and subsequent child mortality. J Pediatr 153, 683-688, 688.e681-683.
34. Gentile, D.A., Henry, J., Katz, A.J., et al., 1997. Inhibition of peripheral blood mononuclear cell proliferation by cardiac glycosides. Ann Allergy Asthma Immunol 78, 466-472.
35. Ghare, S., Patil, M., Hote, P., et al., 2011. Ethanol inhibits lipid raft-mediated TCR signaling and IL-2 expression: potential mechanism of alcohol-induced immune suppression. Alcohol Clin Exp Res 35, 1435-1444.
36. Goodwin, J.S., Atluru, D., Sierakowski, S., et al., 1986. Mechanism of action of glucocorticosteroids. Inhibition of T cell proliferation and interleukin 2 production by hydrocortisone is reversed by leukotriene B4. J Clin Invest 77, 1244-1250.
37. Haley, P.J., Shopp, G.M., Benson, J.M., et al., 1990. The immunotoxicity of three nickel compounds following 13-week inhalation exposure in the mouse. Fundam Appl Toxicol 15, 476-487.
38. Hanke, J.H., Nichols, L.N., Coon, M.E., 1992. FK506 and rapamycin selectively enhance degradation of IL-2 and GM-CSF mRNA. Lymphokine Cytokine Res 11, 221-231.
39. Hansen, J.F., Nielsen, C.H., Brorson, M.M., et al., 2015. Influence of phthalates on in vitro innate and adaptive immune responses. PLoS One 10, e0131168.
40. Hattori, A., Kunz, H.W., Gill, T.J., 3rd, et al., 1987. Thymic and lymphoid changes and serum immunoglobulin abnormalities in mice receiving cyclosporine. Am J Pathol 128, 111-120.
41. He, Y.W., Deftos, M.L., Ojala, E.W., et al., 1998. RORgamma t, a novel isoform of an orphan receptor, negatively regulates Fas ligand expression and IL-2 production in T cells. Immunity 9, 797-806.
42. Henderson, D.J., Naya, I., Bundick, R.V., et al., 1991. Comparison of the effects of FK-506, cyclosporin A and rapamycin on IL-2 production. Immunology 73, 316-321.
43. Himmerich, H., Schonherr, J., Fulda, S., et al., 2011. Impact of antipsychotics on cytokine production in-vitro. J Psychiatr Res 45, 1358-1365.
44. Hu, H., Abedi-Valugerdi, M., Moller, G., 1997. Pretreatment of lymphocytes with mercury in vitro induces a response in T cells from genetically determined low-responders and a shift of the interleukin profile. Immunology 90, 198-204.
45. Huchet, R., Grandjon, D., 1988. Histamine-induced regulation of IL-2 synthesis in man: characterization of two pathways of inhibition. Ann Inst Pasteur Immunol 139, 485-499.
46. Iatropoulos, M.J., Hobson, W., Knauf, V., et al., 1976. Morphological effects of hexachlorobenzene toxicity in female rhesus monkeys. Toxicol Appl Pharmacol 37, 433-444.
47. Kanariou, M., Huby, R., Ladyman, H., et al., 1989. Immunosuppression with cyclosporin A alters the thymic microenvironment. Clin Exp Immunol 78, 263-270.
48. Karas, K., Salkowska, A., Sobalska-Kwapis, M., et al., 2018. Digoxin, an Overlooked Agonist of RORgamma/RORgammaT. Front Pharmacol 9, 1460.
49. Khan, M.M., Melmon, K.L., Fathman, C.G., et al., 1985. The effects of autacoids on cloned murine lymphoid cells: modulation of IL 2 secretion and the activity of natural suppressor cells. J Immunol 134, 4100-4106.
50. Kim, J.H., Park, J.S., 2002. Potentiation of the immunotoxicity of ethanol by acetaminophen in mice. Int Immunopharmacol 2, 15-24.
51. Kim, J.Y., Huh, K., Lee, K.Y., et al., 2009. Nickel induces secretion of IFN-gamma by splenic natural killer cells. Exp Mol Med 41, 288-295.
52. Kim, S.K., Kwon, D.A., Lee, H.S., et al., 2019. Preventive Effect of the Herbal Preparation, HemoHIM, on Cisplatin-Induced Immune Suppression. Evid Based Complement Alternat Med 2019, 3494806.
53. Kloppenburg, M., Verweij, C.L., Miltenburg, A.M., et al., 1995. The influence of tetracyclines on T cell activation. Clin Exp Immunol 102, 635-641.
54. Knight, J.A., Plowman, M.R., Hopfer, S.M., et al., 1991. Pathological reactions in lung, liver, thymus, and spleen of rats after subacute parenteral administration of nickel sulfate. Ann Clin Lab Sci 21, 275-283.
55. Kouchi, Y., Maeda, Y., Ohuchida, A., et al., 1996. Immunotoxic effect of low dose cisplatin in mice. J Toxicol Sci 21, 227-233.
56. Kucharz, E.J., Sierakowski, S.J., 1990. Studies on immunomodulatory properties of isoniazid. II. Effect of isoniazid on interleukin 2 production and interleukin 2-receptor expression. J Hyg Epidemiol Microbiol Immunol 34, 207-211.
57. Labuzek, K., Kowalski, J., Gabryel, B., et al., 2005. Chlorpromazine and loxapine reduce interleukin-1beta and interleukin-2 release by rat mixed glial and microglial cell cultures. Eur Neuropsychopharmacol 15, 23-30.
58. Landewe, R.B., Miltenburg, A.M., Verdonk, M.J., et al., 1995. Chloroquine inhibits T cell proliferation by interfering with IL-2 production and responsiveness. Clin Exp Immunol 102, 144-151.
59. Lee, J., Lim, K.T., 2012. SJSZ glycoprotein (38 kDa) modulates expression of IL-2, IL-12, and IFN-gamma in cyclophosphamide-induced Balb/c. Inflamm Res 61, 1319-1328.
60. Lehmann, D.M., Williams, W.C., 2018. Development and utilization of a unique in vitro antigen presentation co-culture model for detection of immunomodulating substances. Toxicol In Vitro 53, 20-28.
61. Lemster, B., Woo, J., Strednak, J., et al., 1992. Cytokine gene expression in murine lymphocytes activated in the presence of FK 506, bredinin, mycophenolic acid, or brequinar sodium. Transplant Proc 24, 2845-2846.
62. Loose, L.D., Silkworth, J.B., Pittman, K.A., et al., 1978. Impaired host resistance to endotoxin and malaria in polychlorinated biphenyl- and hexachlorobenzene-treated mice. Infect Immun 20, 30-35.
63. Lu, Z., Liu, F., Chen, L., et al., 2015. Effect of Chronic Administration of Low Dose Rapamycin on Development and Immunity in Young Rats. PLoS One 10, e0135256.
64. Maeda, M., Ishii, H., Tanaka, S., et al., 2010. Suppressive efficacies of antimicrobial agents against human peripheral-blood mononuclear cells stimulated with T cell mitogen and bacterial superantigen. Arzneimittelforschung 60, 760-768.
65. Meredith, C., Scott, M.P., 1994. Altered gene expression in immunotoxicology screening in vitro: Comparison with ex vivo analysis. Toxicol In Vitro 8, 751-753.
66. Miller, L.C., Kaplan, M.M., 1992. Serum interleukin-2 and tumor necrosis factor-alpha in primary biliary cirrhosis: decrease by colchicine and relationship to HLA-DR4. Am J Gastroenterol 87, 465-470.
67. Miller, T.E., Golemboski, K.A., Ha, R.S., et al., 1998. Developmental exposure to lead causes persistent immunotoxicity in Fischer 344 rats. Toxicol Sci 42, 129-135.
68. Munson, A.E., Sanders, V.M., Douglas, K.A., et al., 1982. In vivo assessment of immunotoxicity. Environ Health Perspect 43, 41-52.
69. Nalesnik, M.A., Todo, S., Murase, N., et al., 1987. Toxicology of FK-506 in the Lewis rat. Transplant Proc 19, 89-92.
70. Northoff, H., Carter, C., Oppenheim, J.J., 1980. Inhibition of concanavalin A-induced human lymphocyte mitogenic factor (Interleukin-2) production by suppressor T lymphocytes. J Immunol 125, 1823-1828.
71. Palacios, R., Sugawara, I., 1982. Hydrocortisone abrogates proliferation of T cells in autologous mixed lymphocyte reaction by rendering the interleukin-2 Producer T cells unresponsive to interleukin-1 and unable to synthesize the T-cell growth factor. Scand J Immunol 15, 25-31.
72. Pally, C., Tanner, M., Rizvi, H., et al., 2001. Tolerability profile of sodium mycophenolate (ERL080) and mycophenolate mofetil with and without cyclosporine (Neoral) in the rat. Toxicology 157, 207-215.
73. Parenti, D.M., Simon, G.L., Scheib, R.G., et al., 1988. Effect of lithium carbonate in HIV-infected patients with immune dysfunction. J Acquir Immune Defic Syndr 1, 119-124.
74. Parthasarathy, N.J., Kumar, R.S., Devi, R.S., 2005. Effect of methanol intoxication on rat neutrophil functions. J Immunotoxicol 2, 115-121.
75. Peterson, K.P., Van Hirtum, M., Peterson, C.M., 1997. Dapsone decreases the cumulative incidence of diabetes in non-obese diabetic female mice. Proc Soc Exp Biol Med 215, 264-268.
76. Poluektova, L.Y., Huggler, G.K., Patterson, E.B., et al., 1999. Involvement of protein kinase A in histamine-mediated inhibition of IL-2 mRNA expression in mouse splenocytes. Immunopharmacology 41, 77-87.
77. Quemeneur, L., Flacher, M., Gerland, L.M., et al., 2002. Mycophenolic acid inhibits IL-2-dependent T cell proliferation, but not IL-2-dependent survival and sensitization to apoptosis. J Immunol 169, 2747-2755.
78. Ress, N.B., Hailey, J.R., Maronpot, R.R., et al., 2003. Toxicology and carcinogenesis studies of microencapsulated citral in rats and mice. Toxicol Sci 71, 198-206.
79. Riesbeck, K., 1999. Cisplatin at clinically relevant concentrations enhances interleukin-2 synthesis by human primary blood lymphocytes. Anticancer Drugs 10, 219-227.
80. Ringerike, T., Ulleras, E., Volker, R., et al., 2005. Detection of immunotoxicity using T-cell based cytokine reporter cell lines ("Cell Chip"). Toxicology 206, 257-272.
81. Roche, Y., Fay, M., Gougerot-Pocidalo, M.A., 1988. Enhancement of interleukin 2 production by quinolone-treated human mononuclear leukocytes. Int J Immunopharmacol 10, 161-167.
82. Saito, R., Hirakawa, S., Ohara, H., et al., 2011. Nickel differentially regulates NFAT and NF-kappaB activation in T cell signaling. Toxicol Appl Pharmacol 254, 245-255.
83. Salazar, V., Castillo, C., Ariznavarreta, C., et al., 2004. Effect of oral intake of dibutyl phthalate on reproductive parameters of Long Evans rats and pre-pubertal development of their offspring. Toxicology 205, 131-137.
84. Santarelli, L., Bracci, M., Mocchegiani, E., 2006. In vitro and in vivo effects of mercuric chloride on thymic endocrine activity, NK and NKT cell cytotoxicity, cytokine profiles (IL-2, IFN-gamma, IL-6): role of the nitric oxide-L-arginine pathway. Int Immunopharmacol 6, 376-389.
85. Schleuning, M.J., Duggan, A., Reem, G.H., 1989. Inhibition by chlorpromazine of lymphokine-specific mRNA expression in human thymocytes. Eur J Immunol 19, 1491-1495.
86. Sfikakis, P.P., Souliotis, V.L., Katsilambros, N., et al., 1996. Downregulation of interleukin-2 and apha-chain interleukin-2 receptor biosynthesis by cisplatin in human peripheral lymphocytes. Clin Immunol Immunopathol 79, 43-49.
87. She, Y., Wang, N., Chen, C., et al., 2012. Effects of aluminum on immune functions of cultured splenic T and B lymphocytes in rats. Biol Trace Elem Res 147, 246-250.
88. Sheikhi, A., Jaberi, Y., Esmaeilzadeh, A., et al., 2007. The effect of cardiovascular drugs on pro-inflammatory cytokine secretion and natural killer activity of peripheral blood mononuclear cells of patients with chronic heart failure in vitro. Pak J Biol Sci 10, 1580-1587.
89. Silvestrini, B., Lisciani, R., Barcellona, P.S., 1967. Anti-granuloma and thymolytic activity of certain drugs. Eur J Pharmacol 1, 240-246.
90. Song, Y., Han, S., Kim, H., et al., 2006. Effects of mizoribine on MHC-restricted exogenous antigen presentation in dendritic cells. Arch Pharm Res 29, 1147-1153.
91. Sookoian, S., Castano, G., Flichman, D., et al., 2004. Effects of ribavirin on cytokine production of recall antigens and phytohemaglutinin-stimulated peripheral blood mononuclear cells. (Inhibitory effects of ribavirin on cytokine production). Ann Hepatol 3, 104-107.
92. Sugiyama, K., Ueda, H., Ichio, Y., et al., 1995. Improvement of cisplatin toxicity and lethality by juzen-taiho-to in mice. Biol Pharm Bull 18, 53-58.
93. Synzynys, B.I., Sharetskii, A.N., Kharlamova, O.V., 2004. [Immunotoxicity of aluminum chloride]. Gig Sanit, 70-72.
94. Sztein, M.B., Simon, G.L., Parenti, D.M., et al., 1987. In vitro effects of thymosin and lithium on lymphoproliferative responses of normal donors and HIV seropositive male homosexuals with AIDS-related complex. Clin Immunol Immunopathol 44, 51-62.
95. Takai, K., Jojima, K., Sakatoku, J., et al., 1990. Effects of FK506 on rat thymus: time-course analysis by immunoperoxidase technique and flow cytofluorometry. Clin Exp Immunol 82, 445-449.
96. Tam, R.C., Pai, B., Bard, J., et al., 1999. Ribavirin polarizes human T cell responses towards a Type 1 cytokine profile. J Hepatol 30, 376-382.
97. Tsukue, N., Toda, N., Tsubone, H., et al., 2001. Diesel exhaust (DE) affects the regulation of testicular function in male Fischer 344 rats. J Toxicol Environ Health A 63, 115-126.
98. Turka, L.A., Dayton, J., Sinclair, G., et al., 1991. Guanine ribonucleotide depletion inhibits T cell activation. Mechanism of action of the immunosuppressive drug mizoribine. J Clin Invest 87, 940-948.
99. Van Dijk, H., Bloksma, N., Rademaker, P.M., et al., 1979. Differential potencies of corticosterone and hydrocortisone in immune and immune-related processes in the mouse. Int J Immunopharmacol 1, 285-292.
100. Van Wauwe, J., Aerts, F., Van Genechten, H., et al., 1996. The inhibitory effect of pentamidine on the production of chemotactic cytokines by in vitro stimulated human blood cells. Inflamm Res 45, 357-363.
101. Vandebriel, R.J., Meredith, C., Scott, M.P., et al., 1998. Effects of in vivo exposure to bis(tri-n-butyltin)oxide, hexachlorobenzene, and benzo(a)pyrene on cytokine (receptor) mRNA levels in cultured rat splenocytes and on IL-2 receptor protein levels. Toxicol Appl Pharmacol 148, 126-136.
102. Vargova, M., Wagnerova, J., Liskova, A., et al., 1993. Subacute immunotoxicity study of formaldehyde in male rats. Drug Chem Toxicol 16, 255-275.
103. Vos, J.G., van Logten, M.J., Kreeftenberg, J.G., et al., 1979. Hexachlorobenzene-induced stimulation of the humoral immune response in rats. Ann N Y Acad Sci 320, 535-550.
104. Vos, J.G., Van Loveren, H., 1994. Developments of immunotoxicology methods in the rat and applications to the study of environmental pollutants. Toxicol In Vitro 8, 951-956.
105. Wagner, W., Sachrajda, I., Pulaski, L., et al., 2011. Application of cellular biosensors for analysis of bioactivity associated with airborne particulate matter. Toxicol In Vitro 25, 1132-1142.
106. Wagner, W., Walczak-Drzewiecka, A., Slusarczyk, A., et al., 2006. Fluorescent Cell Chip a new in vitro approach for immunotoxicity screening. Toxicol Lett 162, 55-70.
107. Wang, Y., Walker, C., Stadler, B.M., et al., 1984. Transcription and translation dependent induction of interleukin 2 (IL-2) and IL-2 receptors. Immunol Lett 8, 227-231.
108. Wilson, R., Fraser, W.D., McKillop, J.H., et al., 1989. The "in vitro" effects of lithium on the immune system. Autoimmunity 4, 109-114.
109. Yamamoto, N., Sakai, F., Yamazaki, H., et al., 1996. Effect of FR167653, a cytokine suppressive agent, on endotoxin-induced disseminated intravascular coagulation. Eur J Pharmacol 314, 137-142.
110. Yoshimura, N., Matsui, S., Hamashima, T., et al., 1989. Effect of a new immunosuppressive agent, FK506, on human lymphocyte responses in vitro. II. Inhibition of the production of IL-2 and gamma-IFN, but not B cell-stimulating factor 2. Transplantation 47, 356-359.
111. Zhang, W.Z., Yong, L., Jia, X.D., et al., 2013. Combined subchronic toxicity of bisphenol A and dibutyl phthalate on male rats. Biomed Environ Sci 26, 63-69.
